# Feasibility, Acceptability, and Test Performance of Point-of-Care Nucleic Acid Tests for HIV Testing and Viral Load Monitoring in the United States: Prospective Longitudinal Mixed-Methods Study

**DOI:** 10.2196/84625

**Published:** 2026-07-23

**Authors:** Lisa A Niemann, Lauren R Violette, Andy Cornelius-Hudson, Maggie Murphy, Carl Capili, Mindy Dai, Anjuli Wagner, Sarah Frey, Shireesha Dhanireddy, Shaneil E Taylor, Karen W Hoover, Joanne D Stekler

**Affiliations:** 1 Department of Medicine, University of Washington, 325 Ninth Avenue, Box 359931, Seattle, WA, 98104, United States, 1 206 744 8887, 1 206 744 3693; 2 Harvard Pilgrim Health Care Institute, Boston, MA, United States; 3 Seattle’s LGBTQ+ Center, Seattle, WA, United States; 4 Department of Biomedical Informatics and Medical Education, University of Washington, Seattle, WA, United States; 5 Department of Global Health, University of Washington, Seattle, WA, United States; 6 Oak Ridge Institute for Science and Education, Oak Ridge, TN, United States; 7 Centers for Disease Control and Prevention, Division of HIV/AIDS Prevention, Atlanta, GA, United States; 8 Department of Epidemiology, University of Washington, Seattle, WA, United States

**Keywords:** HIV testing, point-of-care tests, nucleic acid tests, viral load testing, point-of-care nucleic-acid tests, point-of-care viral load tests

## Abstract

**Background:**

With shorter window periods than serologic point-of-care (POC) tests and faster turnaround than laboratory-based nucleic acid tests (NAT), POC NATs could improve early detection of HIV. Furthermore, semiquantitative POC NAT may provide real-time monitoring for persons with HIV. There have been limited evaluations of POC NAT implementation in the United States.

**Objective:**

This paper describes the protocols and procedures used to evaluate the acceptability and feasibility of POC NAT implementation and test performance in clinical and community settings in Seattle, Washington.

**Methods:**

The Greater Access and Impact through POC NAT (GAIN) study was a Centers for Disease Control and Prevention–funded study that enrolled participants at a Ryan White–funded, hospital-based clinic (Madison Clinic) and a community site (Seattle’s LGBTQ+ Center [the Center]). Persons seeking HIV testing, nonoccupational postexposure prophylaxis, or pre-exposure prophylaxis (PrEP) enrolled at either site received the standard of care plus the SAMBA II HIV-1 Qualitative Whole Blood Test (SAMBA Qual) and pooled laboratory HIV NAT, and persons with HIV enrolled at the Center received standard-of-care sexually transmitted infection testing plus the SAMBA II HIV-1 Semiquantitative Whole Blood Test (SAMBA Semi-Q) and laboratory viral load testing. Persons with HIV from Madison Clinic were recruited into a randomized clinical trial (RCT) to compare the clinical standard of care with the addition of the SAMBA Semi-Q (backed up by Food and Drug Administration–approved laboratory NAT). All participants completed a demographic survey during their study visit and contributed prospective data via electronic medical records. Subsets of participants from each group were invited to complete a postvisit acceptability survey and participate in an individual interview. Patient flow was observed at Madison Clinic to assess the impact of POC testing on clinic visit timing.

**Results:**

From January 2022 to December 2024, 733 participants completed 753 GAIN research visits. Of those, 491 visits were completed by 489 participants seeking HIV testing and/or PrEP at the Center, and 61 visits were completed by 43 participants seeking HIV testing, nonoccupational postexposure prophylaxis, and/or PrEP at Madison Clinic. Test result data will assess test performance, with analyses anticipated in 2027. Seven people with HIV were enrolled at the Center, and 194 people with HIV were enrolled in the Madison Clinic RCT. Data from patients with HIV at Madison Clinic will be used to conduct a survival analysis evaluating the impact of POC NAT on time to viral suppression and to assess test performance, with analyses anticipated in 2026. In-depth acceptability surveys were completed by 193 participants, and 41 interviews were conducted. These data will contribute to qualitative and mixed methods analyses, which are anticipated in 2026. Time-and-motion observations conducted in 2021 and 2023 included 47 patients; results are anticipated in 2026.

**Conclusions:**

The GAIN study is an important evaluation of POC NAT implementation in the United States in community and clinical care settings for HIV diagnosis and viral load monitoring. Results will be reported in future publications.

## Introduction

HIV testing is the critical entry point for the status neutral continuum of care. HIV screening allows for early detection of new HIV infections and linkage of individuals to care and initiate treatment quickly, which can prevent further transmission [1-5]. Furthermore, HIV testing is an important entry point for pre-exposure prophylaxis (PrEP) and is an essential PrEP monitoring tool [6]. Laboratory-based HIV testing can be highly sensitive, but results can take days or weeks and are less likely to be returned to a patient compared to point-of-care (POC) tests [7-9]. In contrast, the POC tests currently approved by the Food and Drug Administration (FDA) provide results in minutes, but they have longer window periods and are therefore more likely to misdiagnose persons with acute HIV infection (AHI) [9-20]. POC nucleic acid tests (NATs) have shorter window periods and can provide a result in about 2 hours, which makes them a promising tool to help identify AHI and provide rapid HIV testing for patients receiving PrEP [21]. No POC NAT is currently FDA-approved, although several are approved for use outside of the United States for early infant and adult diagnosis and viral load monitoring [22-24,25,26]. One study has evaluated POC NAT for HIV testing in the United States, finding that the POC NAT had comparable results to standard-of-care laboratory tests, and staff found the test acceptable, while noting that test timing, cost, and other logistical barriers may remain to implementation in the United States [27]. Staff noted in that study that the POC NAT could provide particular benefit for patients who experience barriers to care and follow-up [27].

For people with HIV, viral load monitoring is a critical component of HIV care, yet laboratory-based viral load testing does not provide real-time feedback while a patient is in the clinic. One study in South Africa suggested that providing results of a near-POC NAT along with tailored counseling was associated with improved care outcomes at 12 months [28,29]. Other studies in Africa have also found that POC NAT allowed for more rapid result delivery, more rapid treatment adjustments, was motivating for patient adherence, and supported patient emotional well-being [30-34]. If implemented in the United States, POC NATs could provide a similar opportunity to provide immediate feedback on viral load levels and tailored adherence counseling [35]. No studies on the implementation of POC NAT for viral load monitoring currently exist in the United States, but formative qualitative work by this group found that providers thought POC NAT would be acceptable, although integration into clinic flow could be a challenge [36], given that most patients spend less time in clinic than the POC NAT turnaround time (TAT) [37].

In 2019, the Centers for Disease Control and Prevention awarded the University of Washington (UW) research funding to conduct the Greater Access and Impact through POC NAT (GAIN) study. The aims of the study are to (1) assess the feasibility of POC NAT implementation among HIV testing programs, (2) evaluate POC NAT sensitivity, specificity, and clinical outcomes, (3) evaluate the impact of a semiquantitative POC NAT on time to virologic suppression among people with HIV in a clinical setting and assess the feasibility of viral load testing in a community setting among people with HIV, and (4) to describe the feasibility of POC NAT implementation in a clinical setting and assess the acceptability of the POC NAT among participants and care providers. In this paper, we will describe the protocols designed to achieve each of these aims, describe participants enrolled, and the planned timelines for data analyses and publication of results.

## Methods

### Description of the Protocol for the GAIN Study

**Figure 1.**
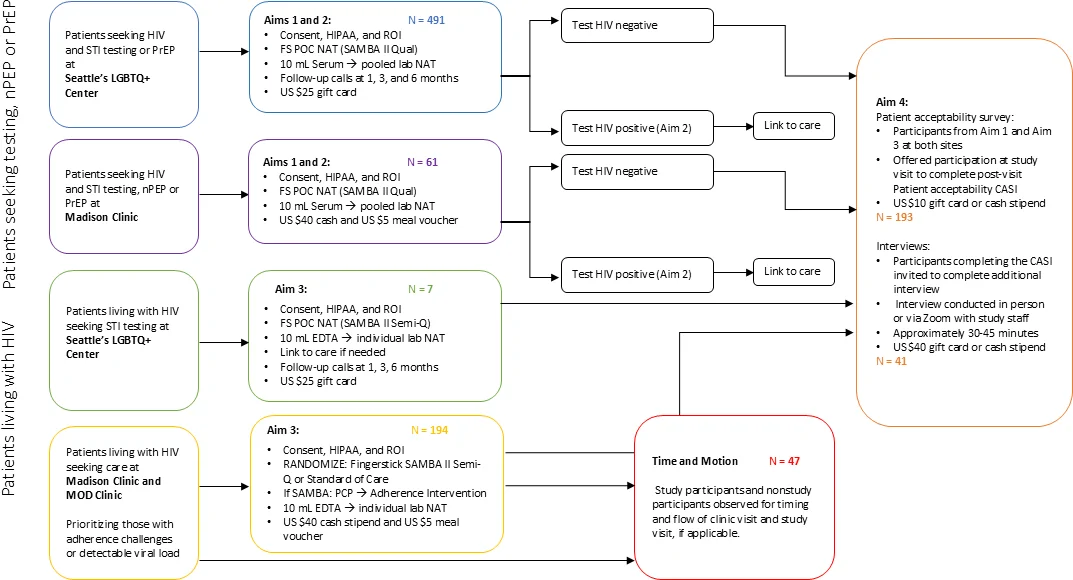
Greater Access and Impact through Point-of-Care Nucleic Acid Test (GAIN) study procedures and total participant visits by aim. CASI (computer-assisted self-interview); EDTA (Ethylenediaminetetraacetic acid); FS (fingerstick); HIPAA (Health Insurance Portability and Accountability); NAT (nucleic acid test); PCP (primary care provider); nPEP (non-occupational postexposure prophylaxis); POC (point of care); PrEP (pre-exposure prophylaxis); ROI (release of information); STI (sexually transmitted infection)

The GAIN study consists of 4 study aims, enrolling study populations at 2 sites (Figure 1). Baseline data obtained from the preenrollment period among nonstudy participants at both sites provided a retrospective preimplementation dataset for comparison with the study participants. Aim 1 evaluated the implementation of the SAMBA II HIV-1 Qual (SAMBA Qual, Diagnostics for the Real World Ltd, Morgan Hill, California, United States) among HIV testing programs via a longitudinal, prospective study of persons without HIV seeking HIV testing, nonoccupational postexposure prophylaxis (nPEP), and PrEP care at Seattle’s LGBTQ+ Center (the Center) and Madison Clinic. Aim 2 evaluated the sensitivity, specificity, and clinical outcomes using the data collected as a part of Aim 1. Aim 3 evaluated the impact of the SAMBA II HIV-1 Semiquantitative (SAMBA Semi-Q, Diagnostics for the Real World Ltd, Morgan Hill, California, United States) on time to virologic suppression via a randomized controlled trial (RCT; NCT04880200), for people with HIV at Madison Clinic and assessed the acceptability of viral load testing outside of a clinical setting among any people with HIV seeking sexually transmitted infection (STI) testing at the Center. Aim 4 examined acceptability and feasibility of the POC NAT using surveys and in-depth interviews with participants and care providers. Finally, a systems engineering component of the study conducted time and motion observations of patient flow through Madison Clinic at one time point preimplementation and 2 time points postimplementation, informed study implementation, and provided information about the impact of POC testing on clinic flow.

### Study Sites

The Center is a community-based organization located in Seattle, Washington, that was established in 1995 and was formerly known as Gay City. The Center’s PrEP program was started by Dr Stekler in 2013 and served as a safety net clinic to link patients to ongoing PrEP care [38].

Madison Clinic is the largest provider of HIV care in Washington State and also offers HIV testing, PEP, and PrEP services. It was established in 1985 and is Ryan White-funded. Madison Clinic also hosts the Moderate Needs Subclinic, or the Moderate Needs (MOD) Clinic. [39] This drop-in subclinic is modeled on the Max Clinic, aimed at engaging the hardest-to-reach people with HIV in need of services [40]. The GAIN study enrolled both patients at Madison Clinic and MOD Clinic. The RCT initially prioritized patients at the MOD Clinic in order to recruit people living with HIV who were more likely to have detectable viral loads but expanded in January 2024 to include additional providers and patients at Madison Clinic.

### POC NAT Selection

During protocol development, the study team considered available POC NATs to identify which test or tests could be used by the GAIN study. Tests considered included the Alere q 1/2 DETECT qualitative [25] and Alere q NAT quantitative [26], the Cepheid Xpert HIV-1 Qualitative [23] and Cepheid Xpert HIV-1 Viral Load XC [41], and the DRW SAMBA Qual [42] and SAMBA Semi-Q [22]. Factors considered included specimen type, volume required, result type, limit of detection, and TAT. The DRW SAMBA tests were selected because they are truly POC, have no requirement for centrifuging (a requirement for the Cepheid Xpert HIV-q Viral Load), and can be performed on fingerstick blood specimens. The SAMBA tests take up to 2 hours to return a result.

### Study Populations and Recruitment

#### GAIN Baseline Data

GAIN baseline datasets were obtained retrospectively to describe the preimplementation populations and outcomes at the Center and Madison Clinic from January 2019 through December 2021. The Center provided a deidentified dataset that linked clients via a dummy identification number across multiple datasets in Access, Google Forms, and InSync electronic medical record (EMR) software. All dates were uniformly time-shifted before transmission to the study team to further deidentify the dataset. Data variables included demographics, HIV and STI testing, and PrEP care.

In order to obtain the Madison Clinic EMR data, we hired the Institute of Translational Health Sciences (ITHS) to pull and deidentify the requested data from the Clinic’s medical record system. ITHS is a collaborative research hub that supports researchers, health care providers, and community organizations working to advance translational science [43]. The study team provided a list of desired variables and worked with ITHS to use these variables to identify visit types for the patients of interest in the time frame of January 2019 through December 2021. These patient types included HIV testing visits, PrEP visits, nPEP visits, patients newly diagnosed with HIV, and patients in HIV care with a detectable viral load. The data were deidentified by creating a dummy identification number, and dates were uniformly time-shifted by an amount unknown to the study team. Data variables included basic demographics, HIV and STI testing, estimated creatinine clearance, viral load testing, and prescribed medications.

Additional data on patients receiving PrEP at Madison Clinic was obtained from the PrEP patient-reported outcomes dataset, a computer-administered self-interview (CASI) on a web-based platform developed for the Center for AIDS Research Network of Integrated Clinical Systems and administered to consenting patients receiving PrEP at 6-month intervals [44]. This dataset included information on adherence and substance use.

#### GAIN Aims 1 and 2 Eligibility (Implement POC NAT Among HIV Testing Programs and Evaluate the Sensitivity, Specificity, and Clinical Outcomes)

Participants were recruited for Aims 1 and 2 if they were at least 18 years old, proficient in English, and seeking HIV testing or PrEP care at the Center or HIV testing, nPEP, or PrEP at Madison Clinic. Participants could not be enrolled if they were testing anonymously. Testing and nPEP participants could be seen only once unless they initiated PrEP. Participants on PrEP could reenroll in the study at any PrEP follow-up visits. We call this group “participants seeking HIV testing.”

#### GAIN Aim 3 Eligibility (Evaluate the Impact of a Semiquantitative POC NAT on Time to Virologic Suppression Among People Living With HIV in a Clinical Setting and Assess Acceptability of Viral Load Testing in a Community Setting Among People Living With HIV)

Participants were recruited for Aim 3 if they were at least 18 years old, proficient in English, living with HIV, and seeking STI testing at the Center or HIV care at the Madison Clinic or MOD Clinic. These patients were only eligible to participate in the study once.

#### GAIN Aim 4 Eligibility (Describe the Feasibility of POC NAT Implementation in a Clinical Setting and Quantify Acceptability and Feasibility of the POC NAT Among Participants and Care Providers)

Participants completing a GAIN study visit were eligible to participate in a postvisit online acceptability survey. Participants who completed this postvisit survey were eligible to participate in a follow-up in-depth interview with study staff. Repeat study participants were invited only once to the acceptability survey and interview. Procedures and recruitment for care provider interviews have previously been described [36].

#### Time and Motion Eligibility

Any Madison Clinic or MOD Clinic patient was eligible to be observed by study staff during their clinic visit to assess pre- and post-study implementation clinic flow.

#### Recruitment Strategies

Participants were screened and approached regarding participation by study staff or provider referral. Patients at Madison Clinic could be called via phone in advance by study staff after chart review for eligibility, and patients at both sites were approached in the waiting room about study participation. A “priority score” was also developed using demographic and behavioral questions from a Center intake form for use in prioritizing clients at the Center for PrEP care and the GAIN study. Development and implementation of this priority score will be described separately.

For participants seeking HIV testing from the Center, we prioritized clients with a reactive POC antibody HIV test, those with any symptoms of AHI, and those who scored high on the priority score. For participants seeking HIV at Madison Clinic, we prioritized enrollment of patients seeking HIV testing and those seeking nPEP. Our recruitment target for these groups was 700 clients from the Center and 150 patients at Madison Clinic.

We aimed to recruit all people with HIV seeking STI testing at the Center. For people with HIV at Madison Clinic, we prioritized patients with low adherence and/or detectable viremia. Study staff attempted to conduct the study visit for these participants before their regular clinic visit, to initiate the POC NAT as soon as possible and maximize the chance that the participant might still be in clinic to receive their result in person. Our recruitment target for these groups was 50 clients at the Center and 212 patients at Madison Clinic.

Participants were invited during their study visit to participate in the extra survey and interview immediately after the study visit or on a later date. Our recruitment target for survey participation was 50 participants seeking HIV testing at each site and 50 people with HIV at each site. Our recruitment target for interview participation was 20 total participants seeking testing (at either site) and 20 total participants with HIV (at either site).

### Study Procedures

#### GAIN Participants Seeking HIV Testing at Both Sites and Participants With HIV at the Center

Enrollment began in January 2022 for patients seeking HIV testing and March 2022 for people with HIV to give study staff time to achieve proficiency in one set of study procedures before adding another group. Participants signed a bi-directional release of information allowing the study team to obtain follow-up data for the year following the study visit and document research test results in the patient EMR. Study staff then completed a linking survey on a secure server including patient identifiable information and the study record of the participant. Then the participant completed a brief study visit survey in REDCap (Research Electronic Data Capture; Vanderbilt University) with demographic questions linked only to the participant’s study record.

After completing study paperwork, study staff collected fingerstick whole blood for the POC NAT. For participants seeking HIV testing, we used the SAMBA Qual, which provides a negative or positive result. European Union–approved materials state that the SAMBA Qual can detect as low as 400 copies of virus as early as 10 days after exposure [42,45]. For participants with HIV, we used the SAMBA Semi-Q, which provides a result of greater or less than 1000 copies per milliliter of blood [22]. Both versions of the test return a result in approximately 2 hours, and neither test is FDA approved. In June 2023, after a series of false-positive test results, we added a second SAMBA Qual using venipuncture whole blood for the participants seeking HIV testing at the Center to provide an additional backup to the fingerstick test.

Study staff collected specimens for all research tests and, if possible, any clinically indicated laboratory tests. HIV testing participants completed standard-of-care HIV testing as part of their visit at both sites, which could include the INSTI HIV-1/HIV-2 Antibody Test (bioLytical Laboratories, Inc) on a fingerstick specimen and/or the laboratory-based HIV-1/HIV-2 Combo enzyme immunoassay (Bio-Rad Laboratories, Inc). Reactive samples were confirmed using the Geenius HIV-1/2 Supplemental Assay (Bio-Rad Laboratories) with reflex testing via the RealTime HIV-1 viral load assay (Abbott Laboratories; for participants seen before November 2023) or the Cobas HIV-1 viral load assay (Roche Diagnostics; for participants seen in November 2023 and after), as appropriate. Laboratory testing was done at the Public Health–Seattle & King County Laboratory and the UW Virology Laboratory.

Initially, a 10 mL serum tube was collected for research HIV testing participants; in June 2023, this changed for participants at the Center to a 4 mL EDTA tube and a 6 mL serum tube to allow for running the SAMBA Qual on venipuncture blood and the laboratory testing on the serum tube. If all screening tests were negative, research laboratory testing also included a laboratory NAT pooled into a single 10-member pool and tested using the RealTime HIV-1 viral load assay (Abbott Laboratories; for participants seen before November 2023) or the Cobas HIV-1 viral load assay (Roche Diagnostics; for participants seen in November 2023 and after). If any screening HIV test was positive, a quantitative HIV-1 RNA test was performed. HIV-positive research participants at the Center had a 10 mL EDTA tube drawn for a laboratory NAT. For all research participants, pooled NAT or individual NAT was performed as the laboratory gold standard for backup of the non-FDA approved SAMBA test [46].

Research and clinic procedures took approximately 1 hour, except for the POC NAT result, and participants were given the option to remain in the clinic to await these results. If the participant was still in the clinic when their POC NAT result was ready, study staff delivered the result to the participant in person. If the participant had left the clinic, they were only notified of their result via a phone call from study staff for unexpected results (ie, a positive SAMBA Qual or a SAMBA Semi-Q result greater than 1000 copies in someone who was expecting to be undetectable). Otherwise, the research results were noted in the EMR, along with clinic laboratory HIV test results. Laboratory results were usually available via the EMR in 5 to 10 business days. Results of the laboratory NAT obtained by the research team were communicated to participants only if test results were discordant. Study staff provided counseling and referrals as appropriate.

Participants at the Center received follow-up phone calls at 1, 3, and 6 months after their study visit in order to check whether they had initiated PrEP (if they tested HIV negative) or linked to HIV care, initiated antiretroviral therapy (ART), and whether they knew their viral load status (if they tested positive for HIV). Staff stopped calling participants at subsequent follow-up time points if they indicated they had initiated PrEP or linked to HIV care. Study staff also obtained follow-up data from Center participants’ EMRs by providing the Center with a list of participants’ medical record numbers, which Center staff used to provide participant follow-up data back to the study team.

Madison Clinic participant follow-up data were captured through patient medical records obtained and transferred monthly to the study staff by ITHS via REDCap. The study team also obtained data on PrEP study participants from the PrEP patient-reported outcomes dataset.

#### GAIN Participants With HIV in the Madison Clinic RCT

Participants in the RCT signed a release of information and completed the linking survey and study visit survey as described above [36]. Then participants in this group were randomized by 1:1, variable-block-size randomization to either receive either the clinic standard of care or the standard of care plus the SAMBA Semi-Q and tailored counseling.

Beginning in April 2023, study staff also performed venipuncture for participants in both arms and drew a 10 mL EDTA tube for a study laboratory NAT and, if possible, for the convenience of the participant, any additional tubes for clinical purposes. This change was made to ensure that all persons had an FDA-approved NAT that could be used as the primary way to guide clinical decision-making.

For participants in both arms, study staff obtained follow-up data from ITHS to monitor patient outcomes for 1 year after the study visit. Viral loads were obtained from the standard-of-care group at the time of the study visit and through up to 1 year of follow-up so that time to virologic suppression could be compared between the 2 arms.

For participants randomized to the intervention arm, study staff also conducted a fingerstick and initiated the SAMBA Semi-Q. After the study procedures were completed, study staff connected the participant with their care team to complete their clinic visit. When the SAMBA Semi-Q result was ready, study staff delivered the result to the participant’s physician. When participants were still in the clinic, the physician delivered the result along with the brief adherence counseling intervention developed for the study in collaboration with clinic providers, the process for which has already been described (Multimedia Appendices 1 and 2) [36]. For participants who had left the clinic, the physician followed up with the patient either via phone or by EMR message, whichever was considered most appropriate. For each participant randomized to the POC NAT arm, the physician reported to the research study whether they discussed the POC NAT result with the participant in person, via phone, or not at all. SAMBA Semi-Q and research laboratory NAT results were manually entered into the EMR by the research team.

#### GAIN Acceptability Survey Participants

An additional acceptability survey was offered to a subgroup of participants beginning in May 2023. A small number of participants were not offered participation for a variety of reasons (eg, study staff forgot, the participant had a complicated visit, and it was logistically best to not offer an additional component). We delayed the offer of acceptability surveys until after the study had been enrolling for approximately 1 year.

At the study visit, study staff offered participants the optional extra survey. Participants who expressed willingness to take the extra survey were emailed a link to the REDCap CASI within 3 days of their study visit. For people with HIV at Madison Clinic, we also began offering the survey immediately after the study visit on a study tablet or laptop to increase access for participants with limited email or internet.

The CASI asked questions about the acceptability of the POC NAT and result delivery. Participants testing for HIV received a survey that included questions on PrEP use and sexual history, and a set of 12 discrete-choice questions about HIV testing preferences (Multimedia Appendix 3). People with HIV received questions on POC NAT acceptability, ART use, viral load testing and cutoff levels, and undetectable = untransmittable (Multimedia Appendix 4). People with HIV at Madison Clinic also received questions about their provider and the delivery of the adherence counseling intervention (Multimedia Appendix 5). Because of the sensitive nature of some of the questions, the survey instructions recommended that participants take the survey in a private setting. The CASI was designed to take about 10 minutes, and participants were sent a US $10 gift card via email or received US $10 cash following survey completion.

#### GAIN Participant Interviews

From June through November 2023, all participants who completed the postvisit acceptability survey were invited to complete a semistructured interview with study staff either in person, via phone, or via Zoom (Zoom Video Communications, Inc.). Participants were contacted via email, and study staff attempted to schedule interviews within 1 month of the participant’s study visit to maximize recall. The conversations were audio-recorded and transcribed by HIPAA (Health Insurance Portability and Accountability Act)–compliant Global Marketing Resources (GMR) Transcription. Once the transcripts were reviewed for accuracy and any identifying information was removed, the original recordings were deleted.

The topics covered in the interviews included more detailed, open-ended questions about the acceptability of the POC NAT and result delivery and, for participants who tested HIV-negative, a discussion of their HIV testing preferences (Multimedia Appendix 6). People with HIV were asked about viral load monitoring and their care preferences, viral load cutoff levels, and undetectable = untransmittable, and the adherence intervention conversation with their provider (Multimedia Appendices 7 and 8).

When thematic saturation appeared to have been reached for participants who tested HIV-negative, recruitment continued until February 2024 only for participants who tested HIV-negative who were persons of color or transgender, in order to increase the diversity of the sample. These interviews continued with people with HIV through the duration of the study.

#### GAIN Provider Interviews

We also interviewed providers at Madison Clinic to obtain input on the acceptability and feasibility of POC NAT use in the clinical setting from the provider perspective. In a first round of preimplementation key informant interviews with providers at Madison Clinic, we explored perspectives on the possible utility of POC NAT and integration into clinic flow. We also solicited provider input on the development of an ultra-brief adherence counseling intervention using motivational interviewing and problem-solving counseling techniques, the results of which have been described elsewhere [36]. Brief, postimplementation interviews were conducted with providers who successfully delivered a POC NAT result to a participant to gain insight into the process of test result delivery and adherence counseling. More formal interviews are planned at the completion of the project to solicit feedback on the utility of the SAMBA Semi-Q.

#### GAIN Time and Motion Observations

Time-and-motion studies were conducted at three times: February-July 2021 before study enrollment began to inform study implementation, March-April 2023 for a post-COVID pandemic repeat assessment (during a regulatory study pause in enrollment), and November 2023 to October 2024 during study enrollment to assess the impact of the POC NAT on clinic flow [37]. Patients were observed by study staff from the time they arrived in the waiting room until they departed the clinic, with staff noting the type of visit and the time of each subsequent segment of the patient visit (eg, front desk check-in, waiting room time, and time the patient spent with each provider type). The final time-and-motion dataset conducted during study enrollment also noted participant time spent on research procedures. That final dataset evaluating the impact of POC NAT implementation will be reported elsewhere.

### Data Collection and Statistical Analysis

GAIN study data sources are shown in Table 1. We present demographic data for client visits in the baseline dataset and GAIN participants in Tables 2-4 as descriptive statistics. When data from the study visit survey were missing, we filled in missing values whenever possible with EMR data. Demographic data are presented at the participant level and include gender, race, insurance, and PrEP and ART status. Planned statistical analyses are described with anticipated timelines for completion.

**Table 1.** Data sources for the Greater Access and Impact through Point-of-Care Nucleic Acid Test study.

Dataset	Source	Participants	Data included
Baseline data from Seattle’s LGBTQ+^a^ Center	Seattle’s LGBTQ+ Center InSync EMR^b^, Google Forms intake	Preimplementation, nonstudy participants	Demographics, HIV POC^c^ and laboratory test results, PrEP^d^ prescriptions
Baseline data from Madison Clinic	Madison Clinic Epic EMR, PrEP PRO^e^	Preimplementation, nonstudy participants	Demographics, HIV POC and laboratory test results, PrEP visits, viral load results, and AR^f^T medications
Linking survey	REDCap^g^, study staff completed at the study visit	All participants	Patient EMR, Study ID, name, date of birth, and contact information
Study visit survey	REDCap, participant completed at the study visit	All participants	Patient demographics, substance use, and ART use
Test results	REDCap, study staff completed at the study visit and after the study visit	All participants	POC and laboratory HIV test results
Follow-up phone calls	REDCap, study staff completed after the study visit	All participants at Seattle’s LGBTQ+ Center	PrEP initiation, ART use, viral load results, and connection to HIV care
Follow-up acceptability survey	REDCap, participant completed after the study visit	Subset of all participants	POC NAT^h^ perspectives, PrEP preferences, and viral load testing preferences
Follow-up in-depth interview	Qualitative interview conducted after the study visit	Subset of participants completing the acceptability survey	POC NAT perspectives, PrEP preferences, and viral load testing preferences
Prospective data from Seattle’s LGBTQ+ Center	Seattle’s LGBTQ+ Center InSync EMR, Google Forms intake	All participants at Seattle’s LGBTQ+ Center	Demographics, HIV POC and laboratory test results, and PrEP prescriptions
Prospective data from Madison Clinic	Madison Clinic Epic EMR, PrEP PRO	All participants at Madison Clinic	Demographics, HIV POC and laboratory test results, PrEP visits, viral load results, and ART medications
Time and motion data	Observational data on patient flow through Madison Clinic collected by study staff	Nonparticipants, Madison participants with HIV	Timing of clinic and study visit components

a LGBTQ+: lesbian, gay, bisexual, transgender, queer, and others.

b EMR: electronic medical record.

c POC: point-of-care.

d PrEP: pre-exposure prophylaxis.

e PRO: patient-reported outcomes.

f ART: antiretroviral therapy

g REDCap: Research Electronic Data Capture

h NAT: nucleic acid test.

**Table 2.** Characteristics of baseline visits by clients at Seattle’s LGBTQ+^a^ Center (January 2019-December 2021) and participants in the GAIN^b^ study who were seeking HIV and STI^c^ testing and PrEP^d^ (January 2022-August 2024).

Characteristic	Baseline client visits (Jan 2019-Dec 2021)			GAIN^b^ study participant visits	
	HIV and STI^c^ testing visits	PrEP^d^ visits	Total visits	Visits by participants seeking HIV testing and PrEP (Jan 2022- Aug 2024)	Visits by participants with HIV seeking STI testing (Mar 2022-Aug 2024)
Total, n (%)	6906 (96.2)	272 (3.8)	7178 (100)	489 (100)	7 (100)
Age (years), median (IQR)	30 (26-38)	28 (24-33)	30 (26-37)	30 (25-35)	57 (41-61)
Gender, n (%)					
Cisgender man	5487 (79.5)	216 (79.4)	5703 (79.5)	333 (68.1)	7 (100)
Cisgender woman	430 (6.2)	0 (0.0)	430 (6.0)	35 (7.2)	0 (0)
Transgender man	99 (1.4)	2 (0.7)	101 (1.4)	13 (2.7)	0 (0)
Transgender woman	190 (2.8)	13 (4.8)	203 (2.8)	17 (3.5)	0 (0)
Nonbinary or genderqueer (AMAB^e^)	307 (4.4)	14 (5.1)	321 (4.5)	51 (10.4)	0 (0)
Nonbinary or genderqueer (AFAB)^f^	206 (3.0)	1 (0.4)	207 (2.9)	30 (6.1)	0 (0)
Nonbinary or genderqueer (another gender or intersex at birth, missing sex at birth)	18 (0.3)	0 (0.0)	18 (0.3)	6 (1.2)	0 (0)
Missing	169 (2.4)	26 (9.6)	195 (2.7)	4 (0.8)	0 (0)
Race or ethnicity, n (%)					
American Indian or Alaska Native	40 (0.6)	2 (0.7)	42 (0.6)	1 (0.2)	0 (0)
Asian	1015 (14.7)	26 (9.6)	1041 (14.5)	69 (14.1)	0 (0)
Black or African American	628 (9.1)	34 (12.5)	662 (9.2)	28 (5.7)	0 (0)
Native Hawaiian or Other Pacific Islander	75 (1.1)	10 (3.7)	85 (1.2)	2 (0.4)	1 (14.3)
White	3375 (48.9)	77 (28.3)	3452 (48.1)	278 (56.9)	5 (71.4)
Latinx^g^	1194 (17.3)	77 (28.3)	1271 (17.7)	72 (14.7)	1 (14.3)
Multiracial^h^	451 (6.5)	16 (5.9)	467 (6.5)	36 (7.4)	0 (0)
Middle Eastern or South Asian	37 (0.5)	2 (0.7)	39 (0.5)	ND^i^	ND
Missing or no response	91 (1.3)	28 (10.3)	119 (1.7)	3 (0.6)	0 (0)
Insurance, n (%)					
Public insurance	391 (5.7)	27 (9.9)	418 (5.8)	87 (17.8)	2 (28.6)
Private insurance	1663 (24.1)	46 (16.9)	1709 (23.8)	250 (51.1)	5 (71.4)
Unspecified type	2648 (38.3)	0 (0.0)	2648 (36.9)	11 (2.2)	0 (0)
No insurance	2138 (31.0)	47 (17.3)	2185 (30.4)	121 (24.7)	0 (0)
Not sure or missing	66 (1.0)	152 (55.9)	218 (3.0)	20 (4.3)	0 (0)
Substance use, n (%)					
IDU^j^ (past year)^k^	224 (3.4)	12 (11.1)	236 (3.5)	ND^i^	ND
IDU (past 3 mo)	ND	ND	ND	4 (0.8)	0 (0)
Meth (past 3 mo)^l^	65 (2.1)	6 (5.2)	71 (2.2)	13 (2.7)	0 (0)
HIV status and PrEP or ART^m^ use, n (%)					
HIV negative	5937 (86.0)	N/A^n^	N/A	489 (100)	0 (0.0)
Currently on PrEP	929 (15.6)	N/A	N/A	123 (25.5)	N/A
HIV positive	175 (2.5)	N/A	N/A	0 (0)	7 (100)
Currently on ART	55 (31.4)	N/A	N/A	N/A	6 (85.7)
Not currently on ART	15 (8.6)	N/A	N/A	N/A	1 (14.3)
ART status unknown or missing	105 (60.0)	N/A	N/A	N/A	0 (0)
HIV status unknown or missing	794 (11.5)	N/A	N/A	0 (0)	0 (0)

a LGBTQ+: lesbian, gay, bisexual, transgender, queer, and others.

b GAIN: Greater Access and Impact through Point-of-Care Nucleic Acid Test.

c STI: sexually transmitted infection.

d PrEP: pre-exposure prophylaxis.

e AMAB: assigned male at birth

f AFAB: assigned female at birth.

g Participants who selected Latinx and any other race were categorized as Latinx.

h Participants who selected any 2 or more races but not Latinx were categorized as multiracial.

i ND: no data available; data that was not collected for the group listed.

j IDU:

k Asked of 6575 test participants and 108 PrEP participants.

l Asked of 3107 test participants and 108 PrEP participants.

m ART: antiretroviral therapy

n N/A: not applicable; variable does not apply to the group listed.

**Table 3.** Characteristics of baseline visits from patients at Madison Clinic (Jan 2019-Dec 2021) and participants in the GAIN^a^ study seeking HIV and STI^b^ testing, nPEP^c^, or PrEP^d^ (January 2022-August 2024).

Characteristic	Baseline patient visits (January 2019-December 2021)				GAIN^a^ study participant visits (Jan 2022-Aug 2024)
	nPEP visits	HIV testing visits	PrEP visits	Total visits	HIV and STI testing, nPEP, and PrEP visits
Total, n (%)	117 (13.8)	328 (38.8)	400 (47.3)	845 (100)	43 (100)
Age (years), median (IQR)	31 (26-39)	32 (27-42)	33 (27-44.5)	33 (27-43)	35 (31-44)
Gender, n (%)					
Cisgender man	117 (100)	177 (54)	348 (87)	642 (76)	37 (86)
Cisgender woman	0 (0)	150 (45.7)	51 (12.8)	201 (23.8)	1 (2.3)
Transgender man	0 (0)	0 (0)	0 (0)	0 (0)	0 (0)
Transgender woman	0 (0)	0 (0)	0 (0)	0 (0)	2 (4.7)
Nonbinary or genderqueer (AMAB^e^)	0 (0)	0 (0)	0 (0)	0 (0)	1 (2.3)
Nonbinary or genderqueer (AFAB^f^)	0 (0)	0 (0)	0 (0)	0 (0)	0 (0)
Nonbinary (unknown sex at birth)	0 (0.0)	1 (0.3)	1 (0.3)	2 (0.2)	0 (0.0)
Another gender identity	0 (0.0)	0 (0.0)	0 (0.0)	0 (0.0)	2 (4.7)
Missing	0 (0.0)	0 (0.0)	0 (0.0)	0 (0.0)	0 (0.0)
Race or ethnicity, n (%)					
American Indian or Alaska Native	1 (0.9)	3 (0.9)	10 (2.5)	14 (1.7)	0 (0.0)
Asian	20 (17.1)	33 (10.1)	36 (9.0)	89 (10.5)	2 (4.7)
Black or African American	12 (10.3)	44 (13.4)	25 (6.3)	81 (9.6)	2 (4.7)
Hispanic or Latinx^g^	20 (17.1)	13 (4.0)	40 (10.0)	73 (8.6)	11 (25.6)
Native Hawaiian or Other Pacific Islander	1 (0.9)	1 (0.3)	2 (0.5)	4 (0.5)	1 (2.3)
White	51 (43.6)	178 (54.3)	198 (49.5)	427 (50.5)	27 (62.8)
Multiracial^h^	1 (0.9)	7 (2.1)	11 (2.8)	19 (2.2)	0 (0.0)
Missing or no response	11 (9.4)	49 (14.9)	78 (19.5)	138 (16.3)	0 (0.0)
Insurance, n (%)					
Public insurance	39 (33.3)	94 (28.7)	121 (30.3)	329 (38.9)	19 (44.2)
Private insurance	55 (47.0)	125 (38.1)	149 (37.3)	254 (30.1)	16 (37.2)
Another insurance	5 (4.3)	62 (18.9)	75 (18.8)	142 (16.8)	0 (0.0)
Missing	18 (15.4)	47 (14.3)	55 (13.8)	120 (14.2)	9 (20.9)
PrEP use (reported by participant), n (%)					
Never	ND^i^	ND	ND	ND	7 (16.3)
Previously	ND	ND	ND	ND	6 (14.0)
Currently	ND	ND	ND	ND	30 (69.8)
Missing	ND	ND	ND	ND	0 (0.0)
Reason for visit (reported by study staff), n (%)					
HIV testing	ND	ND	ND	ND	6 (14.0)
PEP	ND	ND	ND	ND	6 (14.0)
PrEP start	ND	ND	ND	ND	5 (11.6)
PrEP persistence	ND	ND	ND	ND	26 (60.5)

a GAIN: Greater Access and Impact through Point-of-Care Nucleic Acid Test.

b STI: sexually transmitted infection.

c nPEP: nonoccupational postexposure prophylaxis.

d PrEP: pre-exposure prophylaxis.

e AMAB: assigned male at birth.

f AFAB: assigned female at birth

g Participants who selected Latinx and any other race were categorized as Latinx.

h Participants who selected any 2 or more races but not Latinx were categorized as multiracial.

i ND: no data available; data that wasn’t collected for this variable for this group

**Table 4.** Characteristics of baseline visits by patients at Madison Clinic (Jan 2019-Dec 2021) and GAIN^a^ study participants living with HIV (Jan 2022-Aug 2024).

Characteristics	Baseline visits by patients at Madison Clinic (Jan 2019-Dec 2021)			GAIN^a^ study visits (Jan 2022-Aug 2024)
	Patients with first reactive HIV result	Patients with detectable HIV viral load	Total patients	Participants with HIV
Total, n (%)	146 (22.2)	511 (77.8)	657 (100)	194 (100)
Age (years), median (IQR)	34 (28‐47)	43 (32‐52)	41 (31‐51)	44.5 (35-57)
Gender, n (%)				
Cisgender man	117 (80.1)	406 (79.5)	523 (79.6)	144 (74.2)
Cisgender woman	29 (19.9)	104 (20.4)	133 (20.2)	19 (9.8)
Transgender man	0 (0.0)	0 (0.0)	0 (0.0)	0 (0.0)
Transgender woman	0 (0.0)	0 (0.0)	0 (0.0)	10 (5.2)
Nonbinary or genderqueer (AMAB)^b^	0 (0.0)	0 (0.0)	0 (0.0)	15 (7.7)
Nonbinary or genderqueer (AFAB)^c^	0 (0.0)	0 (0.0)	0 (0.0)	0 (0.0)
Nonbinary or genderqueer (unknown sex at birth)	0 (0.0)	1 (0.2)	1 (0.2)	0 (0.0)
Another gender identity	0 (0.0)	0 (0.0)	0 (0.0)	6 (3.1)
Missing	0 (0.0)	0 (0.0)	0 (0.0)	0 (0.0)
Race or ethnicity, n (%)				
American Indian or Alaska Native	0 (0)	14 (2.7)	14 (2.1)	3 (1.6)
Asian	10 (6.8)	20 (3.9)	30 (4.6)	4 (2.1)
Black or African American	43 (29.5)	152 (29.7)	195 (29.7)	53 (27.3)
Hispanic or Latinx^d^	12 (8.2)	21 (4.1)	33 (5.0)	27 (13.9)
Native Hawaiian or Other Pacific Islander	1 (0.7)	8 (1.6)	9 (1.4)	5 (2.6)
White	56 (38.4)	239 (46.8)	295 (44.9)	84 (43.3)
Multiracial^e^	4 (2.7)	4 (0.8)	8 (1.2)	17 (8.8)
Missing or no response	20 (13.7)	20 (3.9)	40 (6.1)	1 (0.5)
Insurance, n (%)				
Public insurance	55 (37.7)	325 (63.6)	380 (29.8)	162 (83.5)
Private insurance	61 (41.8)	135 (26.4)	196 (57.8)	28 (14.4)
Another insurance	12 (8.2)	17 (3.3)	29 (4.4)	1 (0.5)
Missing	18 (12.3)	34 (6.7)	52 (7.9)	3 (1.6)
ART^f^ use, n (%)				
Never	ND^g^	ND	ND	38 (19.6)
Previously	ND	ND	ND	19 (29.4)
Currently	ND	ND	ND	134 (69.1)
missing	ND	ND	ND	3 (1.6)
Laboratory viral load, n (%)^h^				
Detectable (≥40 copies)	N/A^i^	N/A	N/A	63 (32.5)
Undetectable (<40 copies)	N/A	N/A	N/A	113 (58.2)
Laboratory viral load not done	N/A	N/A	N/A	18 (9.3)^j^
Randomization arm, n (%)				
Standard of care	N/A	N/A	N/A	97 (50)
Point-of-care NAT^k^	N/A	N/A	N/A	97 (50)

a GAIN: Greater Access and Impact through Point-of-Care Nucleic Acid Test.

b AMAB: assigned male at birth.

c AFAB: assigned female at birth.

d Participants who selected Latinx and any other race were categorized as Latinx.

e Participants who selected any 2 or more races but not Latinx were categorized as multiracial.

f ART: antiretroviral therapy

g ND: no data available; data that was not collected for the group listed.

h Filled in with clinic electronic medical record if available within 15 days of visit date when study VL not available.

i N/A: not applicable; variable does not apply to the group listed

j Laboratory viral load test was not done for participants enrolled before October 2022 (n=7) and for participants from whom no blood could be drawn due to hard stick (n=8). When available, viral load values were obtained from the clinic electronic medical record.

k NAT: nucleic acid test.

### Ethical Considerations

The study received ethical approval from the UW Human Subjects Division (STUDY 00010387). A waiver of informed consent was granted by the UW Human Subjects Division for baseline data collection and time and motion procedures, as no identifiers were obtained. Study staff performed consent procedures and obtained informed written consent for study participants in Aims 1‐4. To protect participant data, participants from Aims 1‐4 were assigned a study ID, and their study data was stored in connection with that ID. A separate linking survey connected their study ID to their medical record number, which was used to obtain prospective data. A Human Subjects Division–approved Data Safety and Monitoring Plan was used to ensure protective measures to safeguard participant information, including appropriate levels of training for staff, limited data access, secure digital and physical storage places, and data transmission. Madison Clinic participants initially received a US $20 cash incentive and a US $5 meal voucher. Participants at the Center initially did not receive an incentive, but this was amended in April 2023 to provide a US $25 online gift card for consistency between sites. To help boost enrollment, the participation incentive was increased to US $40 in cash and a US $5 meal voucher for participants at Madison Clinic in August 2023. Participants who completed the additional acceptability survey were provided a US $10 electronic gift card upon survey completion. Participants who participated in the interview were provided a US $40 electronic gift card. In August 2024, we began offering incentives in cash as participants noted lack of email access as a barrier for survey and interview participation.

## Results

During the baseline data period of January 2019 to December 2021 at the Center, 6906 unique clients were seen for 8492 HIV and STI testing visits, and 272 clients had 612 PrEP visits (Table 2). Most clients (n=5703, 79.5%) were cisgender men. Almost half were White (n=3452, 48.1%). Nearly one-third (n=2185, 30.4%) reported no insurance. Among testing visit clients, about 86% (n=5937) were HIV negative (though HIV status was missing or unknown for 11.5% [n=794]), of whom, 15.6% (n=929) reported being on PrEP. About 2.5% (n=175) of testing visit patients were living with HIV, and about one-third of those clients reported that they were on ART (n=55, 31.4%), though data were missing for most clients (n=105, 60%).

At Madison Clinic during the same baseline period (Table 3), there were 117 unique patients seen for nPEP, 328 unique patients seen for HIV testing visits, and 400 unique patients seen for PrEP visits. There were 146 patients who tested positive for HIV for the first time and 511 patients with HIV whose viral load was detectable (>40 copies/mL) at some point during that time (Table 4). Among patients seen, 48.1% were White (n=722), 18.4% were Black or African American (n=276), 7.9% were Asian (n=119), and 7.1% were Hispanic or Latinx (n=106). Most (77.6%) were male (n=1065), 22.2% were female (n=334), and only 0.2% were transgender or nonbinary (n=3).

During study enrollment from January 2022 to December 2024, 733 unique people completed 753 GAIN study research visits (Tables 2-4). Of those visits, 491 visits were completed by 489 participants seeking HIV testing and/or PrEP at the Center. Most of those participants were seeking HIV testing (96%), were cisgender men (333/489, 68.1%), were White (278/489, 56.9%), with a median age of 30 (IQR 25‐35) years. About one-quarter (123/489, 25.2%) were currently on PrEP. Seven people with HIV seeking STI testing were enrolled from the Center. These participants were all cisgender men, most were White (n=7, 71.4%), with a median age of 57 (IQR 41‐61) years. Of those participants, 1 was not taking ART but had previously, the other 6 were currently taking ART.

At Madison Clinic, 61 visits were completed by 43 participants seeking HIV testing, nPEP, and/or PrEP. The majority of participants were there for a PrEP persistence visit (n=26, 60.5%). Most of these participants were cisgender men (n=37, 86%) and White (n=27, 62.8%), with a median age of 35 (IQR 31‐44) years, and most (n=30, 69.8%) were on PrEP. At Madison Clinic and MOD Clinic, 194 people with HIV seeking care were enrolled during the same time period. These participants were mostly cisgender men (n=144, 74.2%); 43.3% (n=84) were White, 27.3% (n=53) were Black or African American, and their median age was 44.5 (IQR 35‐57) years. Among Madison and MOD Clinic participants with HIV, 69.1% (n=134) were currently taking ART and 32.5% (n=63) had a detectable viral load (>40 copies/mL) at the time of their study visit. Differences in the populations of participants at the Center and Madison Clinic reflect the different populations seen at the 2 sites.

As part of Aim 4 study activities (Table 5), during a recruitment period that included 228 study visits completed by participants seeking HIV and STI testing at the Center, staff invited 198 to take the postvisit acceptability survey, of whom 133 (67.2%) participants completed. During 6 study visits by PWH at the Center, staff sent participants 4 survey invitations, and 3 participants completed them (75% completion). Among 25 Madison Clinic participant visits for HIV and STI testing, nPEP, and PrEP, 10 of 21 invited participants completed the survey (47.6% completion). During 161 study visits for PWH at Madison and MOD Clinics, 47 of 77 (61%) invited participants completed the survey. Of those who completed the additional surveys, 21 participants seeking HIV and STI testing, nPEP, or PrEP from both sites and 20 people with HIV from both sites also participated in an in-depth interview. Only participants who completed the survey were invited to participate in interviews, and participation rates were at or below 50% for all groups. However, we stopped inviting eligible participants who were seeking HIV and STI testing, nPEP, or PrEP to the interview after we achieved saturation of themes.

**Table 5.** Acceptability survey and interview completions for participants in the GAIN^a^ study from Seattle’s LGBTQ+^b^ Center and Madison Clinic.

Characteristic	Seattle’s LGBTQ+ Center		Madison Clinic		Total, n (%)
	Participants seeking HIV and STI^c^ testing, n (%)	Participants with HIV, n (%)	Participants seeking HIV and STI testing, nPEP^d^, and PrEP^e^, n (%)	Participants with HIV, n (%)	
Total study visits^f^	228 (47)	6 (86)	25 (58)	161 (83)	420 (57)
Survey invitations sent^g^	198 (86.8)	4 (66.7)	21 (84)	77 (47.8)	300 (71.4)
Surveys completed	133 (67.2)	3 (75)	10 (47.6)	47 (61)	193 (64.3)
Interviews completed	16 (12)^h^	1 (33.3)	5 (50)	19 (40.4)	41 (21.2)

a GAIN: Greater Access and Impact through Point-of-Care Nucleic Acid Test.

b LGBTQ+: lesbian, gay, bisexual, transgender, queer, and others.

c STI: sexually transmitted infection.

d nPEP: nonoccupational postexposure prophylaxis.

e PrEP: pre-exposure prophylaxis.

f Includes study visits during which participation in the acceptability survey was being offered

g Includes participants who were offered, agreed, and were eligible to be sent the acceptability survey at their study visit.

h Study staff stopped offering enrollment to all participants after thematic saturation was attained; therefore, not all eligible participants were invited.

Time-and-motion observations were made in 2021 for 25 total patients at Madison Clinic, 13 of whom were from the MOD Clinic (Table 6). We found that the median clinic visit time was 73 (IQR 54‐92) minutes. An additional set of time-and-motion observations, made in 2023 during a pause in study enrollment, included 22 total patients at Madison Clinic (10 of whom were from the MOD Clinic) and revealed a median clinic visit time of 64 (IQR 45‐88) minutes. This demonstrated that most patient visits were shorter than the amount of time the POC NAT used for the study would take to return a result (120 min). This finding led the study team to develop a protocol prioritizing recruitment of participants before their regular clinic visit to initiate the SAMBA test as soon as possible.

**Table 6.** Total time observed in clinic (minutes), including waiting room time, during 1 observation period preenrollment (2021) and 1 period while enrollment was paused (2023).

Year and statistics	MOD^a^ Clinic	Madison Clinic	Total
2021, n	13	12	25
Range	46-301	19-155	19-301
Mean (SD)	93.5 (66.9)	66.8 (36.2)	80.7 (55.0)
Median (IQR)	74 (55-97)	64 (42-85)	73 (54-92)
2023, n	10	12	22
Range	32‐185	45‐95	32‐185
Mean (SD)	75.2 (43.6)	65.3 (17.2)	69.8 (31.6)
Median (IQR)	68 (61-74)	61 (51-80)	64 (45-88)

a MOD: Moderate Needs.

Test results data from Aim 1 participants at both the Center and Madison Clinic will contribute to a performance assessment of the SAMBA Qual, anticipated in 2027. For this analysis, we will calculate the overall specificity and sensitivity of the SAMBA II Qual whole blood test across all tested samples and at HIV-1 RNA thresholds of detectable (>40), 200, 400, 1000, 2000, and 3000 copies/mL. SAMBA performance will be compared to laboratory RNA test results. We hypothesize that the sensitivity and specificity of the SAMBA II Qual will be similar to those of laboratory-based pooled NAT.

Test results and longitudinal follow-up from Aim 3 participants at Madison Clinic will contribute to a survival analysis of time to undetectable viral load, as well as a performance assessment of the SAMBA Semi-Q, anticipated in 2026. We will report the limit of detection using the plasma HIV RNA level as the gold standard, and the agreement (kappa) between SAMBA and plasma HIV RNA levels, dichotomized at 1000 copies/mL and within the range of 1000 copies/mL ± 0.3 log.

For the survival analysis, we will report the time to an undetectable viral load by study arm. We calculated a sample size of 106 per arm, assuming a baseline of 40% of participants with an undetectable viral load, to detect a 20% increase in the proportion of participants with an undetectable viral among people with HIV at Madison Clinic, assuming a 20% loss to follow-up. We hypothesize that the SAMBA Semi-Q will have high agreement with the laboratory-based RNA testing, and that time to virologic suppression will be shorter among participants randomized to the POC NAT-tailored intervention compared with standard of care.

Data from Aim 4 will contribute to a paper on the acceptability of POC NAT among persons testing for HIV, anticipated in 2026 (Table 7). Another paper will address the acceptability of POC NAT among people with HIV using both qualitative data from interviews and participant survey responses, anticipated in 2026. Another paper using data collected as part of Aim 4 activities will explore HIV testing preferences using both the acceptability survey and interview data. We will use a convergent parallel mixed methods design for integration of qualitative and quantitative data. For the interview sample size, we planned to conduct 20 interviews each among the HIV testing group and people with HIV, or until saturation of themes. For surveys, a sample size of 100 HIV testing participants and 100 people with HIV was determined based on feasibility, the literature, and consultation with a choice-modeling expert. We hypothesize that more than 80% of participants surveyed will report a positive experience with POC NAT and that SAMBA implementation will be acceptable in community- and clinic-based settings. However, we hypothesize that the 2-hour TAT will be a limitation to the feasible implementation of POC NAT and that the semiquantitative nature of the test and the limit of detection of 1000 copies/mL may cause some hesitation in the use of the SAMBA Semi-Q.

**Table 7.** Planned publication topics, analyses, and timelines.

Anticipated publication topics	Contributing aims and sites	Planned analysis	Target sample size	Attained sample size	Anticipated publication date
Performance of SAMBA Qual for HIV Testing	Aim 1, Seattle’s LGBTQ+ Center and Madison Clinic	Laboratory and POC^a^ NAT^b^ concordance	850 study visits	550 study visits	2027
Impact of POC NAT on time to undetectable viral load and SAMBA Semi-Q performance	Aim 3, Madison Clinic	Survival analysis, laboratory, and POC NAT concordance	212 study visits with up to 1-year longitudinal follow-up	194 study visits	2026
Acceptability of POC NAT among persons testing for HIV	Aim 4 interviews, Seattle’s LGBTQ+ Center and Madison Clinic	Qualitative	20 or until saturation of themes	21 interviews	2026
HIV testing preferences	Aim 4 interviews and surveys, Seattle’s LGBTQ+ Center and Madison Clinic	Qualitative, discrete choice analysis	20 or until saturation of themes, 100 surveys	21 interviews, 143 surveys	2026
Acceptability of POC NAT among patients with HIV	Aim 4 interviews and surveys, Madison Clinic and Seattle’s LGBTQ+ Center	Qualitative and descriptive statistics	20 or until saturation of themes,100 surveys	20 interviews, 50 surveys	2026
Time and motion	Patients at Madison Clinic and MOD Clinic	Rank-sum tests and flow analysis	10 visits per clinic per observation period	47 patient visits	2026

a POC: point-of-care.

b NAT: nucleic acid test.

Data collected as part of the time-and-motion component of the study will be the subject of a paper anticipated in 2026. We calculated a sample size of 20 participants during each observation period, assuming a mean visit duration without SAMBA of 66 minutes, an SD of 29, α=.05, 80% power, and that 50% of participants would be randomized to receive SAMBA Semi-Q testing. We hypothesize that the average clinic visit will be less than the 2-hour TAT of the POC NAT and that the impact of study procedures on clinic flow will be minimal.

## Discussion

### Principal Findings

The GAIN study was designed to assess the acceptability and feasibility of implementing POC NAT in community and clinical settings. This paper describes the protocol, the populations recruited into the study, and planned analyses. During 3 years of enrollment, the GAIN study conducted 552 visits with HIV testing participants at the 2 study sites. Enrollment of persons seeking HIV testing occurred predominantly at the Center due to the focus of its HIV/STI testing programs. During the same period, the study enrolled 201 people with HIV from both sites. Conversely, recruitment of people with HIV was predominantly from Madison Clinic. Recruitment for the qualitative components of Aim 4 included 193 total poststudy visit surveys and 41 in-depth interviews. At the Center, we found that transgender, nonbinary, and genderqueer people represented a greater proportion of our study population (23.9% of Aim 1 participants) compared with the baseline visits at the Center (11.9%). This may reflect changing demographics of clients at the Center due to shifting priorities of the organization combined with the prioritization of study recruitment among clients at highest likelihood of having HIV [47,48]. Among participants seeking HIV testing at the Center, 25.2% said they were currently on PrEP, which is significantly higher than in the baseline group of participants seeking HIV testing, of whom 15.6% reported being on PrEP. This could also be due to the study focus on recruiting clients who were high priority for PrEP, and could also be influenced by baseline data missingness, as 11.5% of the Center baseline participants did not have a recorded HIV status or PrEP status.

In the baseline population at the Center, 2.5% of clients were people with HIV. Based on that rate, we would expect approximately 50 clients with HIV to visit the Center annually. The GAIN study enrolled only 7 clients with HIV at the Center during the nearly 3 years of enrollment. This low enrollment could be due to some participants not identifying themselves as living with HIV when completing the Center’s intake form, preventing study staff from identifying all eligible clients for recruitment. Future studies may wish to further examine how POC NAT could be used among people with HIV seeking STI testing in a community setting.

At Madison Clinic, the GAIN study enrolled fewer HIV-negative cisgender women (2.3%) than were observed in the Madison Clinic baseline dataset among patients seeking HIV testing (45.7% women) and PrEP (12.8% women). We saw a much higher number of participants identifying as transgender, genderqueer, or persons with another gender identity (11.6%) than were identified in our baseline dataset (0.2% among those seeking HIV testing, nPEP, and PrEP). This is because the study data collection instruments had greater capacity for capturing these gender identities than the EMR, which supplied the baseline data. The GAIN study also enrolled far more patients receiving PrEP (69.8%) than patients seeking HIV testing or nPEP. Future studies could focus more on the utility of the POC NAT among broader clinical patient populations.

Among Madison Clinic patients with HIV, we also enrolled more patients who identified as transgender, genderqueer, or persons with another gender identity (16%) than the patients seen at Madison Clinic during baseline (0.2% among those with a first reactive HIV result and patients with a detectable viral load). Again, this is likely due to constraints of the EMR that was the source of the baseline data. We also saw fewer cisgender women (9.8%) than were seen during the baseline data period (20.4%). The GAIN study aimed to prioritize the enrollment of participants with detectable viral loads to assess the impact of POC NAT on time to viral suppression, and 32.5% of people with HIV enrolled at Madison Clinic had detectable viral loads at their study visit (however, for 9.3% of participants, viral load data were missing). In 2024, about 10% of patients at Madison Clinic had a detectable viral load (>200 copies/mL), so the study was able to oversample this population (data not published). There is a high percentage of patients in the region who have achieved viral suppression in King County; the 2022 HIV Epidemiology Report and Community Profile estimated that 85% of people living with HIV in the region were virally suppressed [49]. Having a sample with a sufficient number of participants with a detectable viral load will strengthen the planned time-to-event analyses for participants achieving an undetectable VL. We also hypothesized that a POC NAT result would likely have a greater impact on patient visits where real-time counseling can be provided for the adherence counseling intervention component.

At both sites, the baseline data were affected by COVID-19 pandemic-related changes to patient flow. In order to address this, we expanded the baseline dataset to include 2021 as well as 2020, when clinic procedures were better adjusted to the impact of the pandemic. The study also experienced other challenges due to the pandemic, including delays in laboratory testing due to overloaded laboratory staff, shifts to the provision of PrEP services via telemedicine, and clinical and laboratory supply shortages. The study also experienced delays due to the initial Office of Management and Budget approval process, which extended more than 2 years beyond the local Institutional Review Board approval process.

The GAIN study is among the first studies to evaluate the implementation of a POC NAT in the United States for screening among participants who were HIV-negative (including patients receiving PrEP) and POC viral load monitoring among PWH. The study team is aware of only 1 other such study in the United States, which assessed a different POC NAT platform for performance in comparison to laboratory NAT for HIV testing and staff acceptability [27]. Participating staff found the test acceptable, and agreement with laboratory NAT was high; however, the authors noted that workflow challenges remained to be resolved, particularly with regards to returning results to participants with the 90-minute TAT. Staff speculated that this would be longer than most patients would want to wait; however, the study did not attempt to return results to participants. The GAIN study will report on efforts to return results to participants. Future studies could focus on the logistics of delivery of POC NAT results. As this technology develops and availability increases in the United States, this protocol can serve as a model for future studies.

### Limitations

The GAIN study has limitations that should be noted. Baseline data and prospective data collected from Madison Clinic and Center datasets could be incomplete because data were deidentified when transferred to the study team and might not perfectly reflect the participants recruited into the study. However, the study team worked closely with data managers from both sites to achieve as complete a dataset as possible. For study visits and follow-up interviews, only participants who spoke English were enrolled, excluding individuals with limited English proficiency. Poststudy survey and interview contact occurred primarily via email, which led to lower uptake and likely excluded participants without access to email. We modified our process in 2024 to allow these activities to be completed in person immediately after the study visit and provided cash reimbursement, which enabled some inclusion of participants without internet access. Though the original proposed protocol called for a comparison of multiple POC NATs, because of challenges with suppliers, the study team was not able to procure any other POC NATs for comparison. Future studies of POC NATs could assess other testing platforms.

Additional analyses based on the GAIN study protocol described here are planned and in progress. Future reports will include papers on the acceptability of the POC NAT among participants seeking HIV testing and people with HIV based on survey and interview results, an assessment of participant HIV testing preferences based on survey and interview results, an assessment of the impact of POC NAT on time to viral suppression, evaluations of the performance of POC NAT in comparison with laboratory NAT, and a final set of Madison Clinic time-and-motion data. Additional POC NAT implementation studies in US settings are needed to assess the acceptability and feasibility of this technology.

## Supplementary material

10.2196/84625 Multimedia Appendix 1 GAIN study presentation for providers: brief counseling skills to improve ART adherence, motivational interviewing, and problem-solving therapy.

10.2196/84625 Multimedia Appendix 2 “A taste of MI” exercise.

10.2196/84625 Multimedia Appendix 3 GAIN study patient acceptability computer-administered self-interview for HIV testing participants.

10.2196/84625 Multimedia Appendix 4 GAIN study patient acceptability computer-administered self-interview for persons with HIV at the Center.

10.2196/84625 Multimedia Appendix 5 GAIN study patient acceptability computer-administered self-interview for persons with HIV at Madison Clinic.

10.2196/84625 Multimedia Appendix 6 GAIN study interview guide for HIV testing participants.

10.2196/84625 Multimedia Appendix 7 GAIN study interview guide for persons with HIV at the Center.

10.2196/84625 Multimedia Appendix 8 GAIN study interview guide for persons with HIV at Madison Clinic.

## Abbreviations

AHI: acute HIV infection
ART: antiretroviral therapy
CASI: computer-administered self-interview
EMR: electronic medical record
FDA: US Food and Drug Administration
GAIN: Greater Access and Impact through Point-of-Care Nucleic Acid Test
GMR: Global Marketing Resources
ITHS: Institute of Translational Health Sciences
MOD: moderate needs
NAT: nucleic acid test
nPEP: nonoccupational postexposure prophylaxis
POC: point-of-care
PrEP: preexposure prophylaxis
RCT: randomized controlled trial
STI: sexually transmitted infection
TAT: turnaround time
UW: University of Washington
