# Correction: Feasibility, Acceptability, and Test Performance of Point-of-Care Nucleic Acid Tests for HIV Testing and Viral Load Monitoring in the United States: Prospective Longitudinal Mixed-Methods Study

**DOI:** 10.2196/114628

**Published:** 2026-11-13

**Authors:** Lisa A Niemann, Lauren R Violette, Andy Cornelius-Hudson, Maggie Murphy, Carl Capili, Mindy Dai, Anjuli Wagner, Sarah Frey, Shireesha Dhanireddy, Shaneil E Taylor, Karen W Hoover, Joanne D Stekler

**Affiliations:** 1 Department of Medicine, University of Washington, 325 Ninth Avenue, Box 359931, Seattle, WA, 98104, United States, 1 206 744 8887, 1 206 744 3693; 2 Harvard Pilgrim Health Care Institute, Boston, MA, United States; 3 Seattle’s LGBTQ+ Center, Seattle, WA, United States; 4 Department of Biomedical Informatics and Medical Education, University of Washington, Seattle, WA, United States; 5 Department of Global Health, University of Washington, Seattle, WA, United States; 6 Oak Ridge Institute for Science and Education, Oak Ridge, TN, United States; 7 Centers for Disease Control and Prevention, Division of HIV/AIDS Prevention, Atlanta, GA, United States; 8 Department of Epidemiology, University of Washington, Seattle, WA, United States

In “Feasibility, Acceptability, and Test Performance of Point-of-Care Nucleic Acid Tests for HIV Testing and Viral Load Monitoring in the United States: Prospective Longitudinal Mixed-Methods Study” [1], the authors noted one error.

In the Funding section, the following has been added:

*The findings and conclusions in this manuscript are those of the authors and do not necessarily represent the views of the Centers for Disease Control and Prevention.*

The correction will appear in the online version of the paper on the JMIR Publications website together with the publication of this correction notice. Because this was made after submission to PubMed, PubMed Central, and other full-text repositories, the corrected article has also been resubmitted to those repositories.
