# Supplementary material for: Feasibility, Acceptability, and Test Performance of Point-of-Care Nucleic Acid Tests for HIV Testing and Viral Load Monitoring in the United States: Prospective Longitudinal Mixed-Methods Study
Source: JMIR Res Protoc. 2026 Jul 23;15:e84625. doi: 10.2196/84625 (PMC13395423; doi:10.2196/84625)
Supplement: Multimedia Appendix 1 [file resprot-v15-e84625-s001.pptx]

## Slide 1
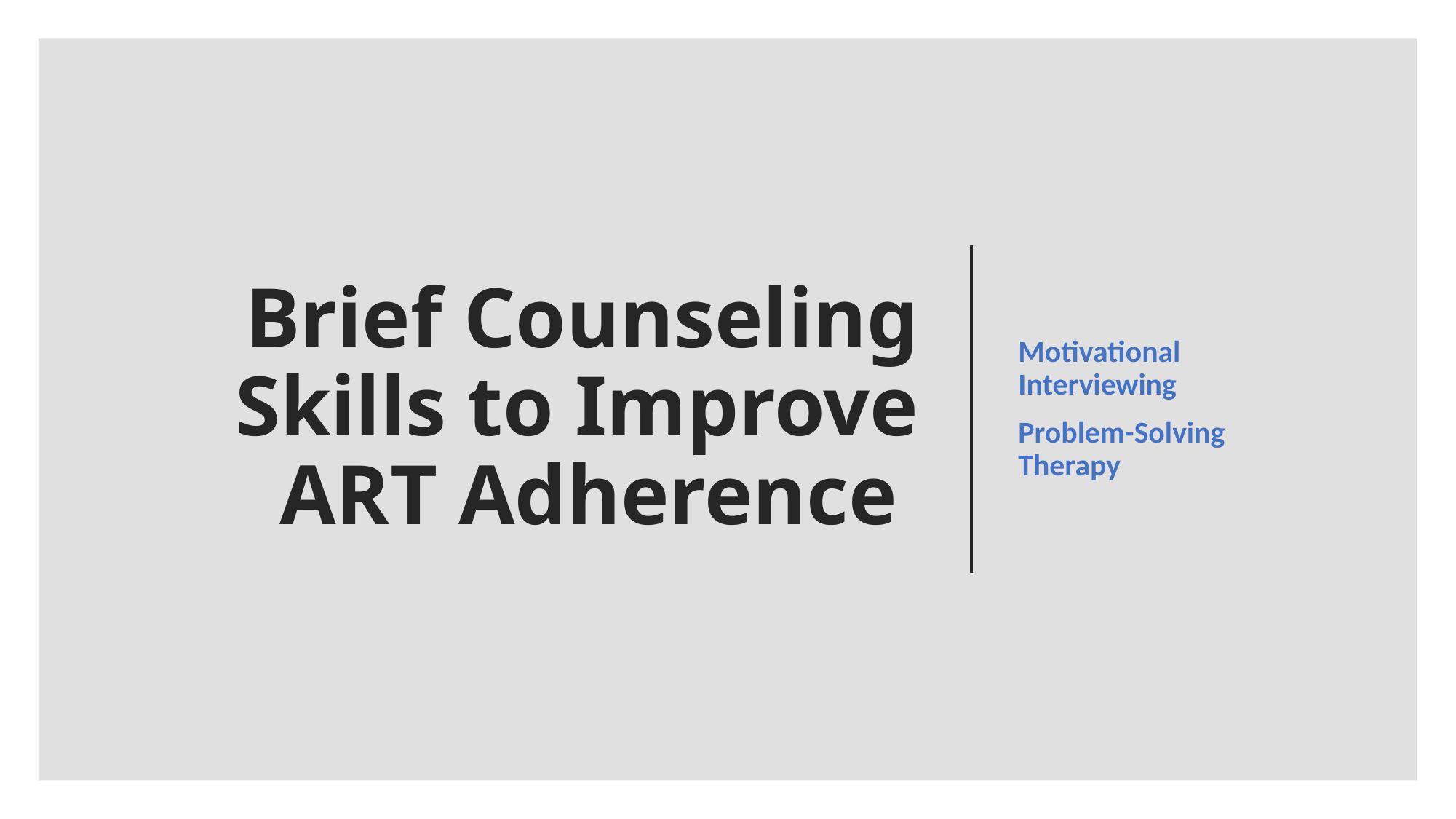

Motivational Interviewing
Problem-Solving Therapy
# Brief Counseling Skills to Improve ART Adherence

## Slide 2
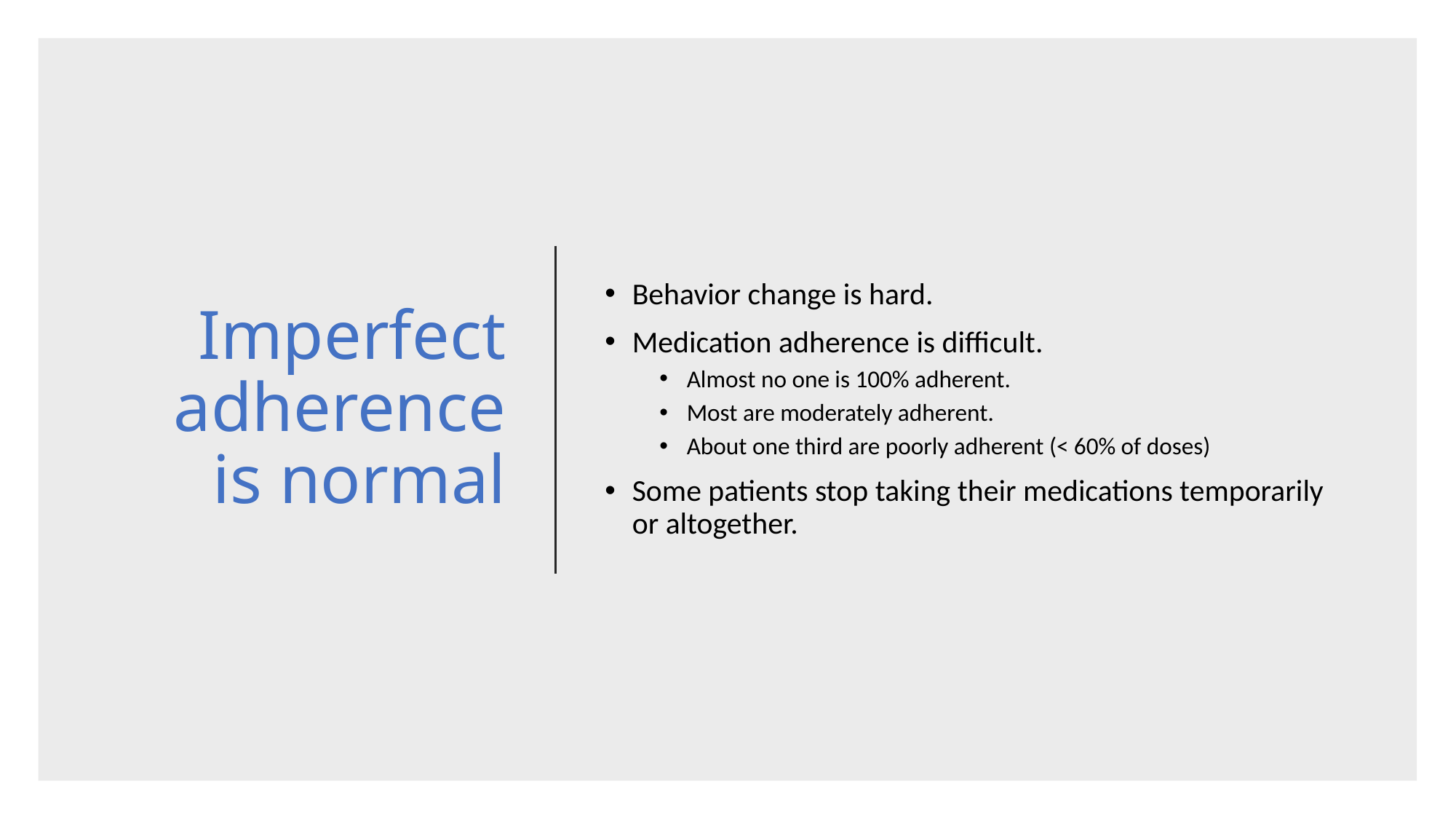

# Imperfect adherence is normal
Behavior change is hard.
Medication adherence is difficult.
Almost no one is 100% adherent.
Most are moderately adherent.
About one third are poorly adherent (< 60% of doses)
Some patients stop taking their medications temporarily or altogether.

## Slide 3
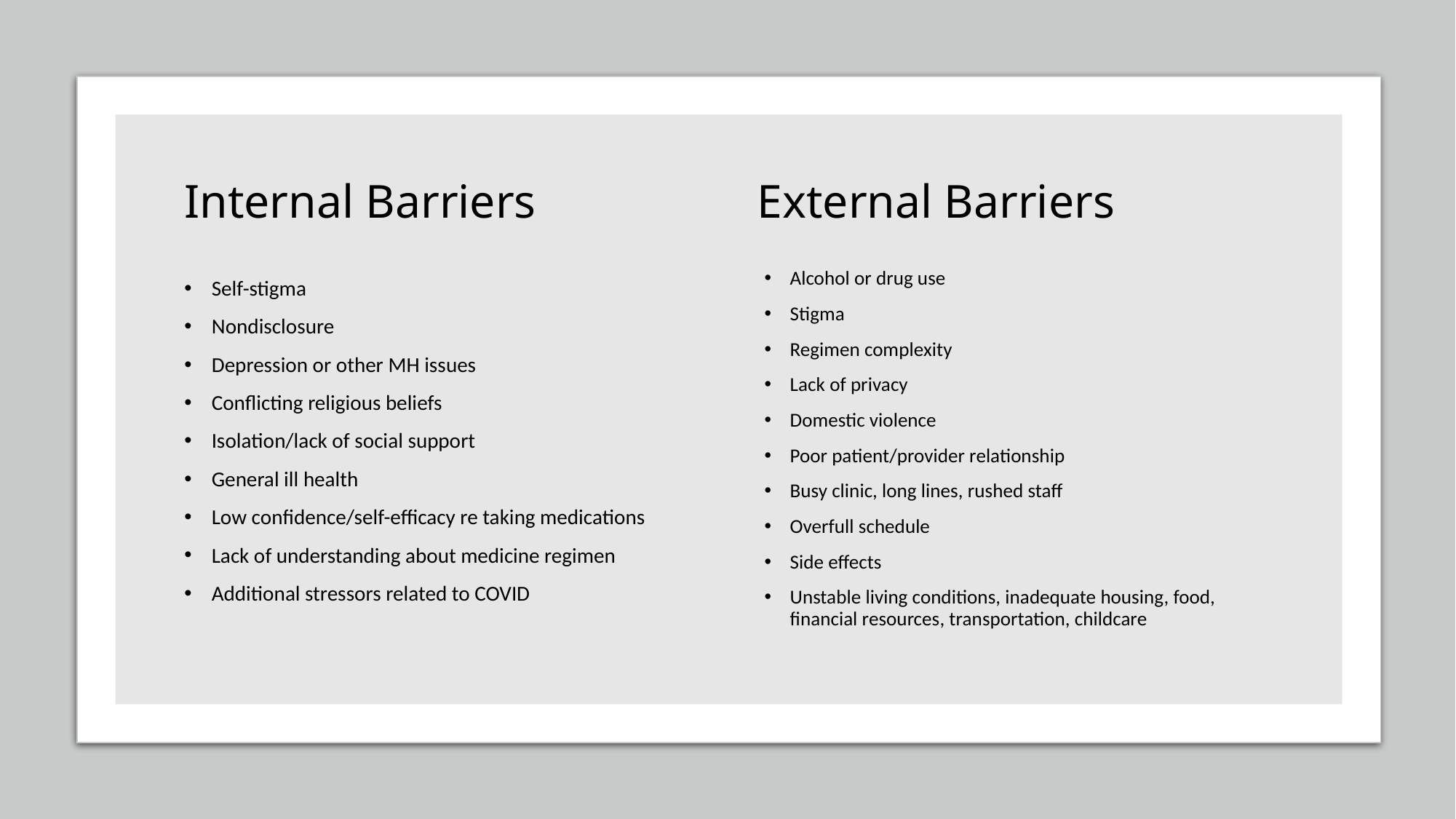

# Internal Barriers			External Barriers
Alcohol or drug use
Stigma
Regimen complexity
Lack of privacy
Domestic violence
Poor patient/provider relationship
Busy clinic, long lines, rushed staff
Overfull schedule
Side effects
Unstable living conditions, inadequate housing, food, financial resources, transportation, childcare
Self-stigma
Nondisclosure
Depression or other MH issues
Conflicting religious beliefs
Isolation/lack of social support
General ill health
Low confidence/self-efficacy re taking medications
Lack of understanding about medicine regimen
Additional stressors related to COVID

## Slide 4
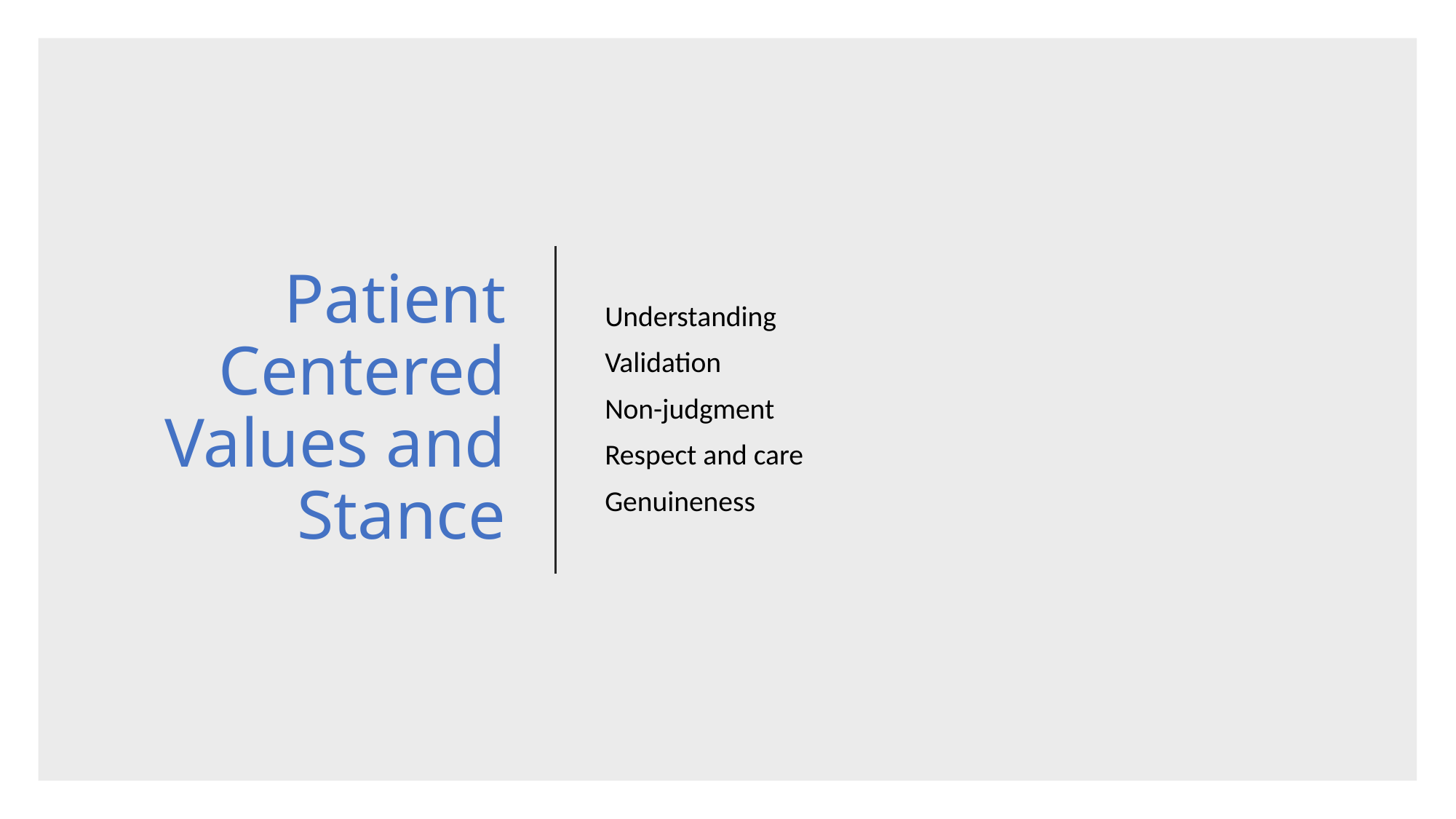

# Patient Centered Values and Stance
Understanding
Validation
Non-judgment
Respect and care
Genuineness

## Slide 5
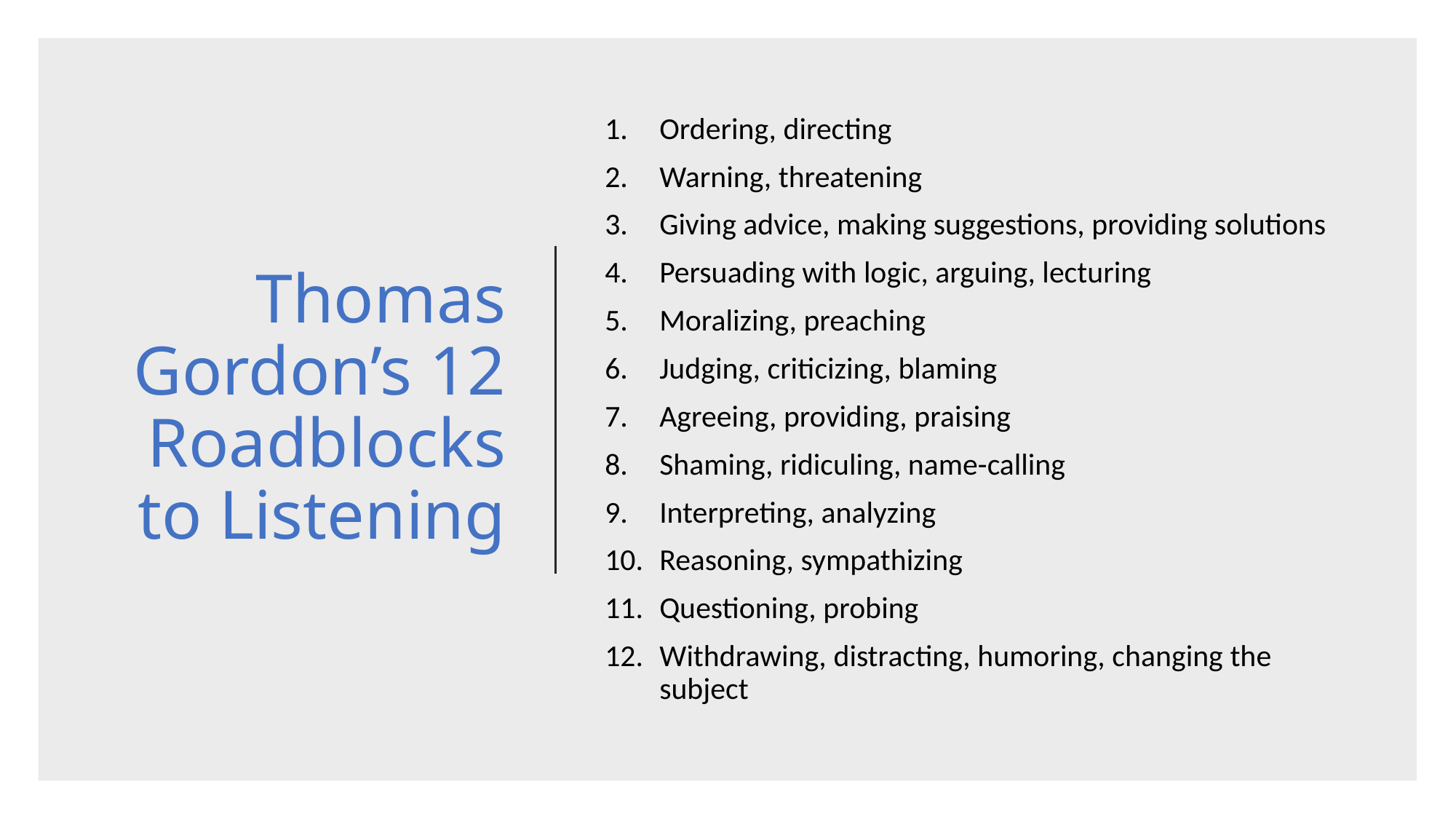

# Thomas Gordon’s 12 Roadblocks to Listening
Ordering, directing
Warning, threatening
Giving advice, making suggestions, providing solutions
Persuading with logic, arguing, lecturing
Moralizing, preaching
Judging, criticizing, blaming
Agreeing, providing, praising
Shaming, ridiculing, name-calling
Interpreting, analyzing
Reasoning, sympathizing
Questioning, probing
Withdrawing, distracting, humoring, changing the subject

## Slide 6
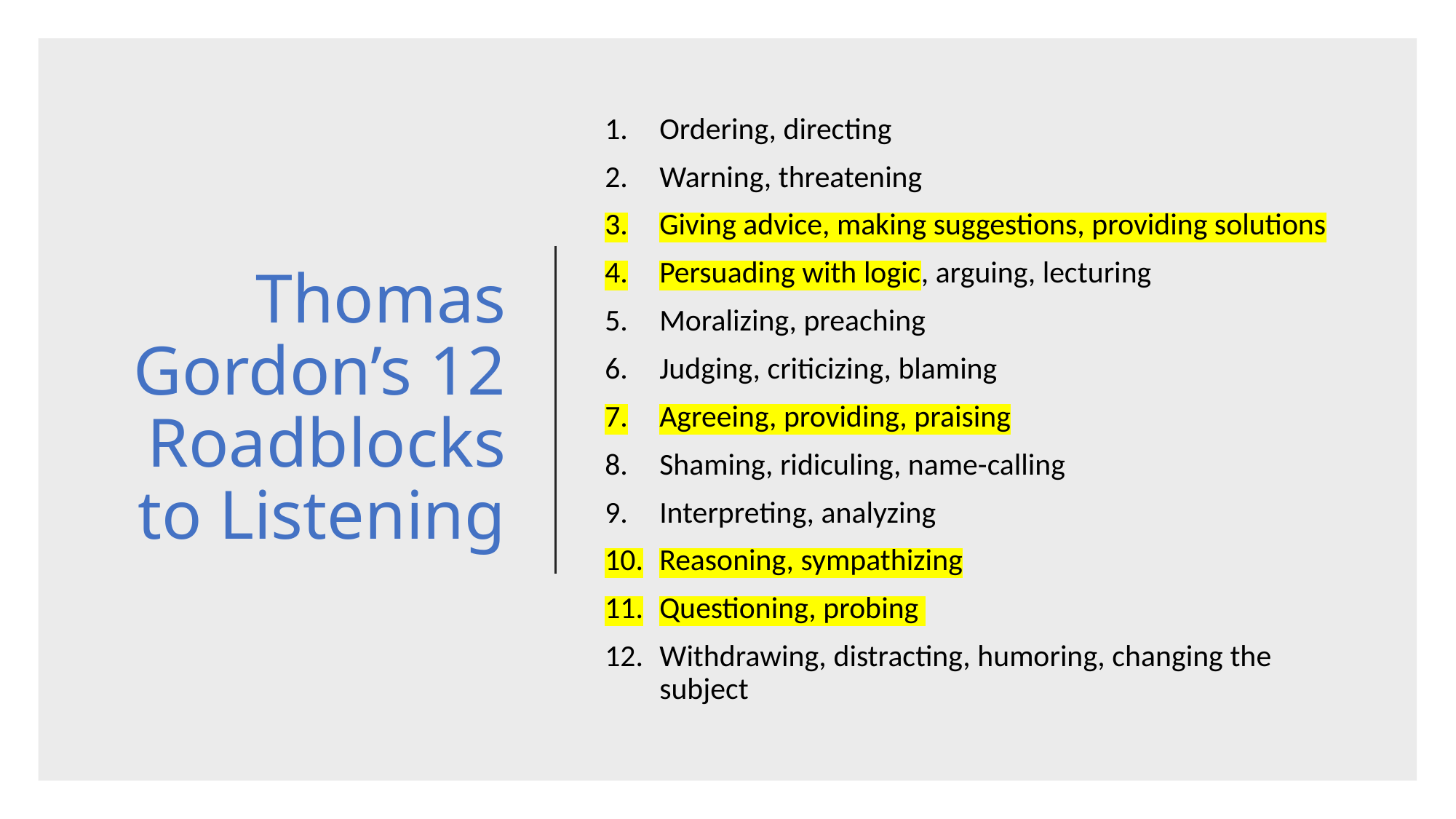

# Thomas Gordon’s 12 Roadblocks to Listening
Ordering, directing
Warning, threatening
Giving advice, making suggestions, providing solutions
Persuading with logic, arguing, lecturing
Moralizing, preaching
Judging, criticizing, blaming
Agreeing, providing, praising
Shaming, ridiculing, name-calling
Interpreting, analyzing
Reasoning, sympathizing
Questioning, probing
Withdrawing, distracting, humoring, changing the subject

## Slide 7
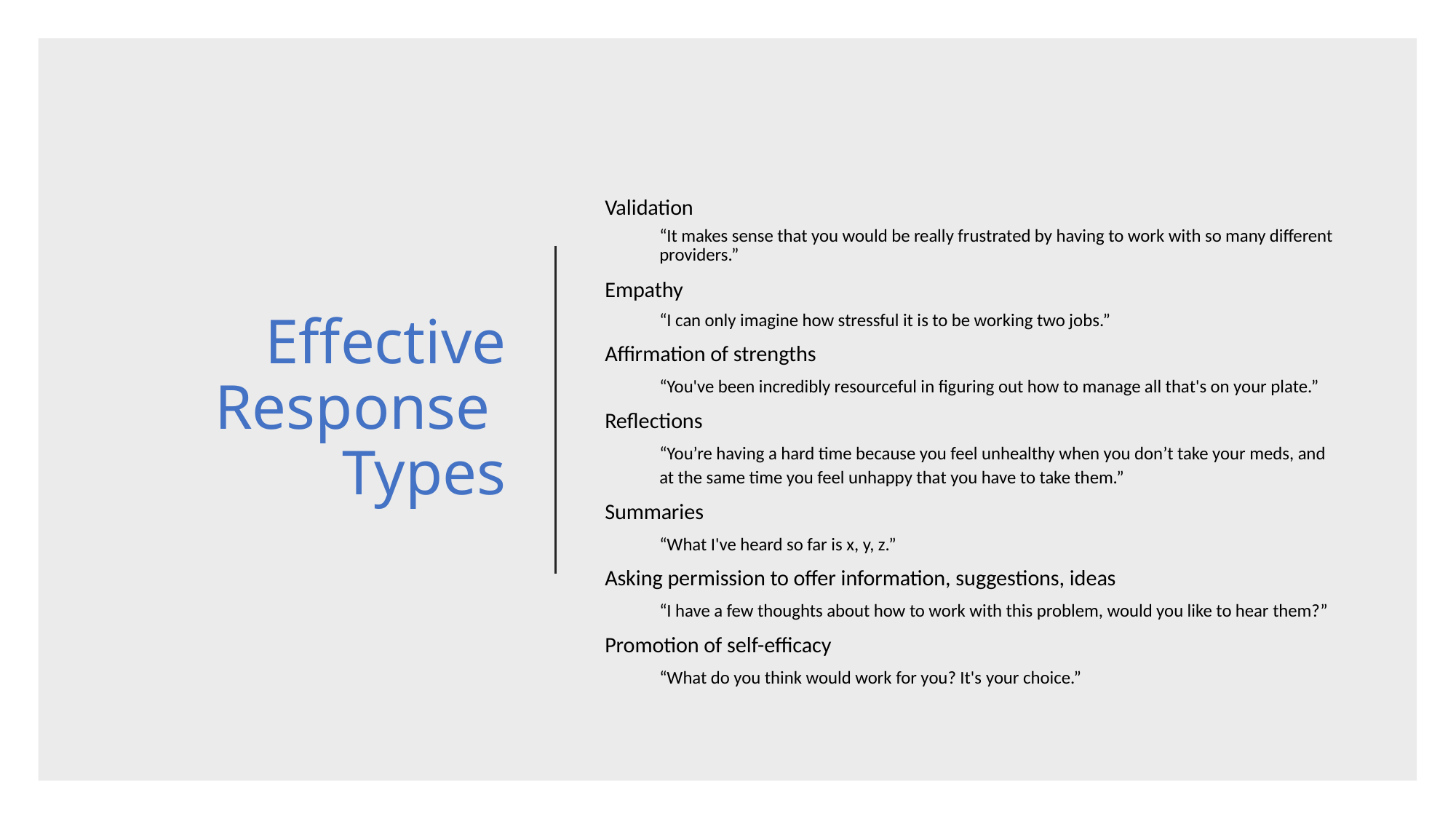

# Effective Response Types
Validation
“It makes sense that you would be really frustrated by having to work with so many different providers.”
Empathy
“I can only imagine how stressful it is to be working two jobs.”
Affirmation of strengths
“You've been incredibly resourceful in figuring out how to manage all that's on your plate.”
Reflections
“You’re having a hard time because you feel unhealthy when you don’t take your meds, and at the same time you feel unhappy that you have to take them.”
Summaries
“What I've heard so far is x, y, z.”
Asking permission to offer information, suggestions, ideas
“I have a few thoughts about how to work with this problem, would you like to hear them?”
Promotion of self-efficacy
“What do you think would work for you? It's your choice.”

## Slide 8
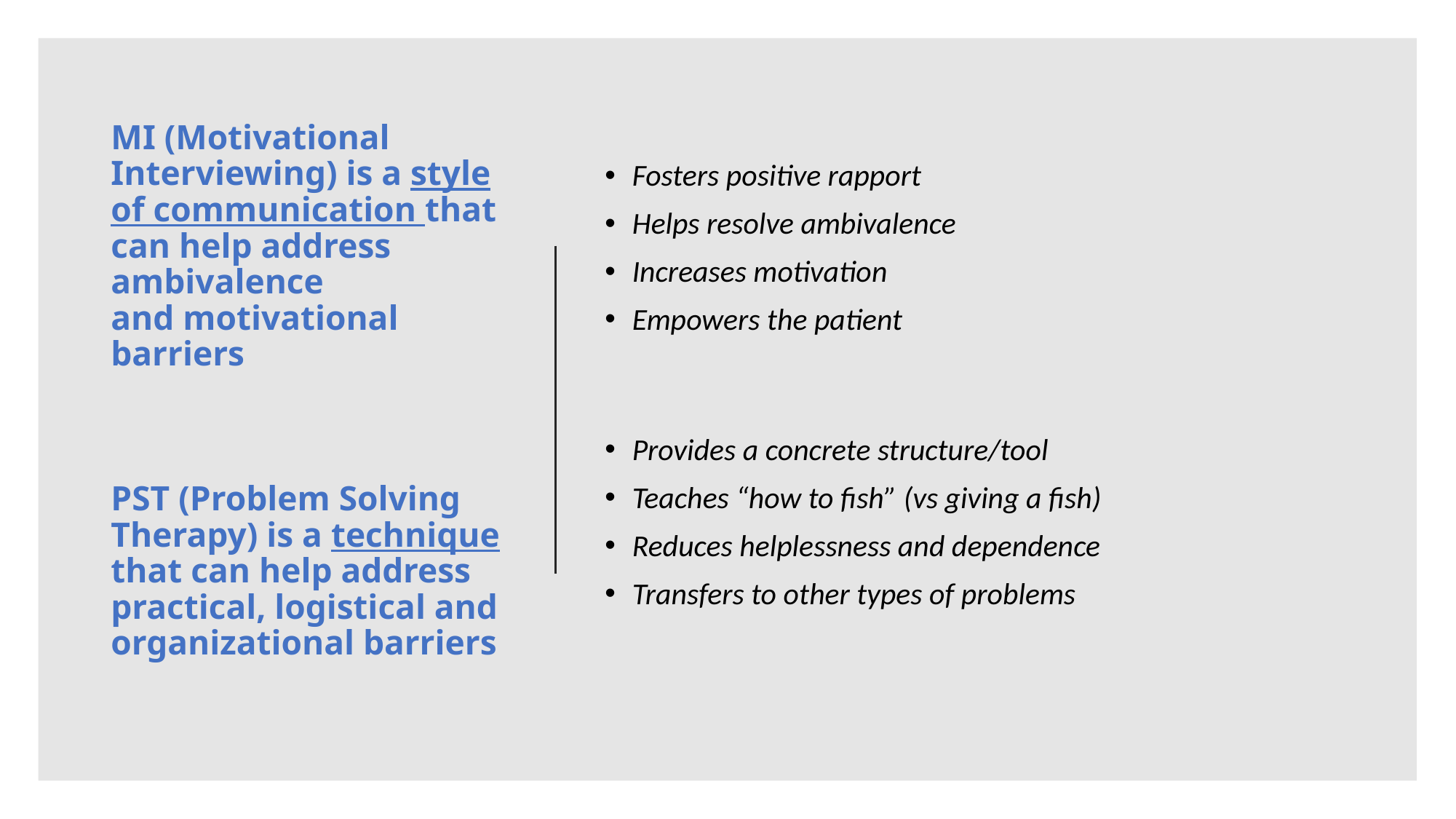

# MI (Motivational Interviewing) is a style of communication that can help address ambivalence and motivational barriers PST (Problem Solving Therapy) is a technique that can help address practical, logistical and organizational barriers
Fosters positive rapport
Helps resolve ambivalence
Increases motivation
Empowers the patient
Provides a concrete structure/tool
Teaches “how to fish” (vs giving a fish)
Reduces helplessness and dependence
Transfers to other types of problems

## Slide 9
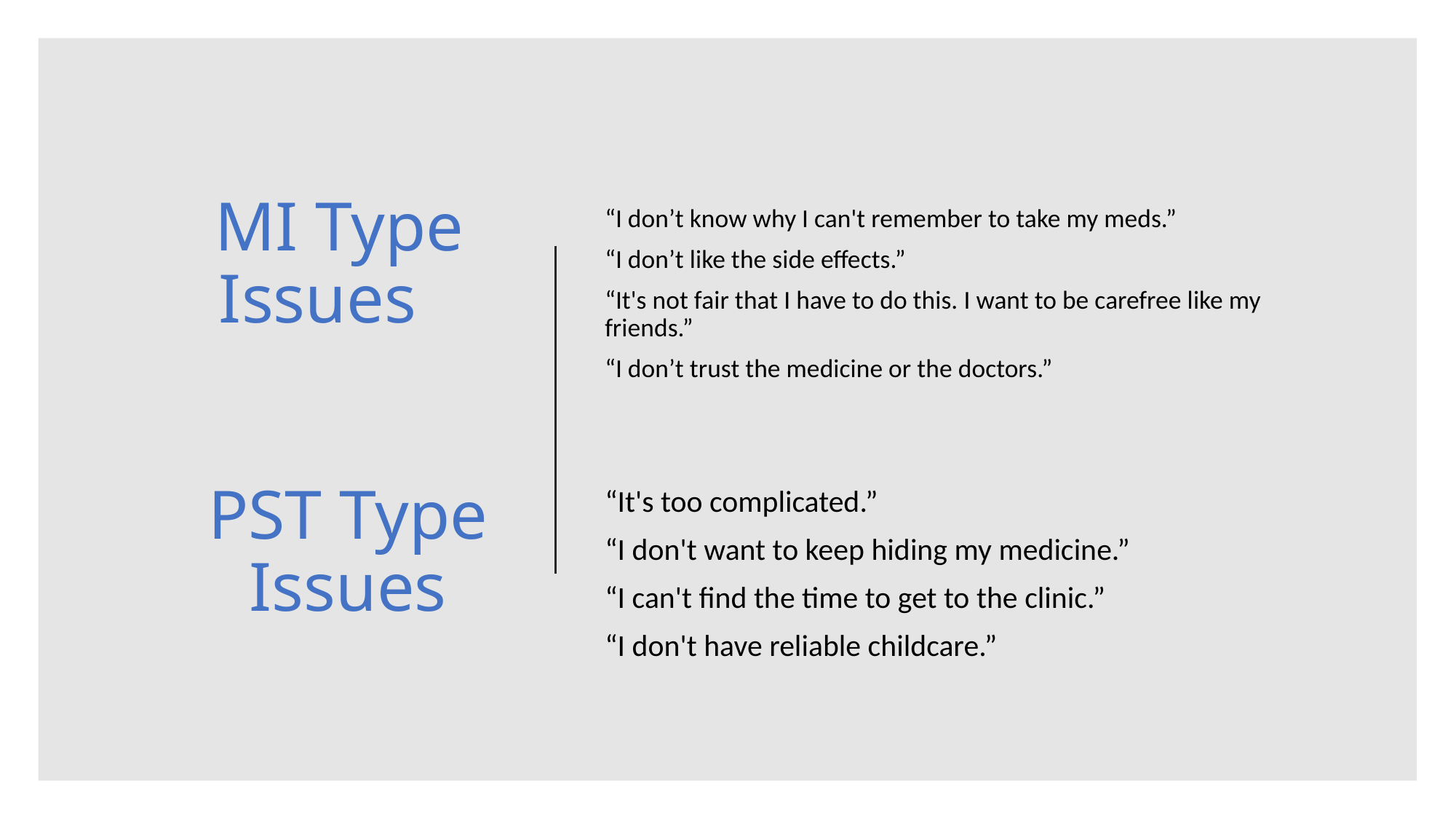

# MI Type Issues PST Type Issues
“I don’t know why I can't remember to take my meds.”
“I don’t like the side effects.”
“It's not fair that I have to do this. I want to be carefree like my friends.”
“I don’t trust the medicine or the doctors.”
“It's too complicated.”
“I don't want to keep hiding my medicine.”
“I can't find the time to get to the clinic.”
“I don't have reliable childcare.”

## Slide 10
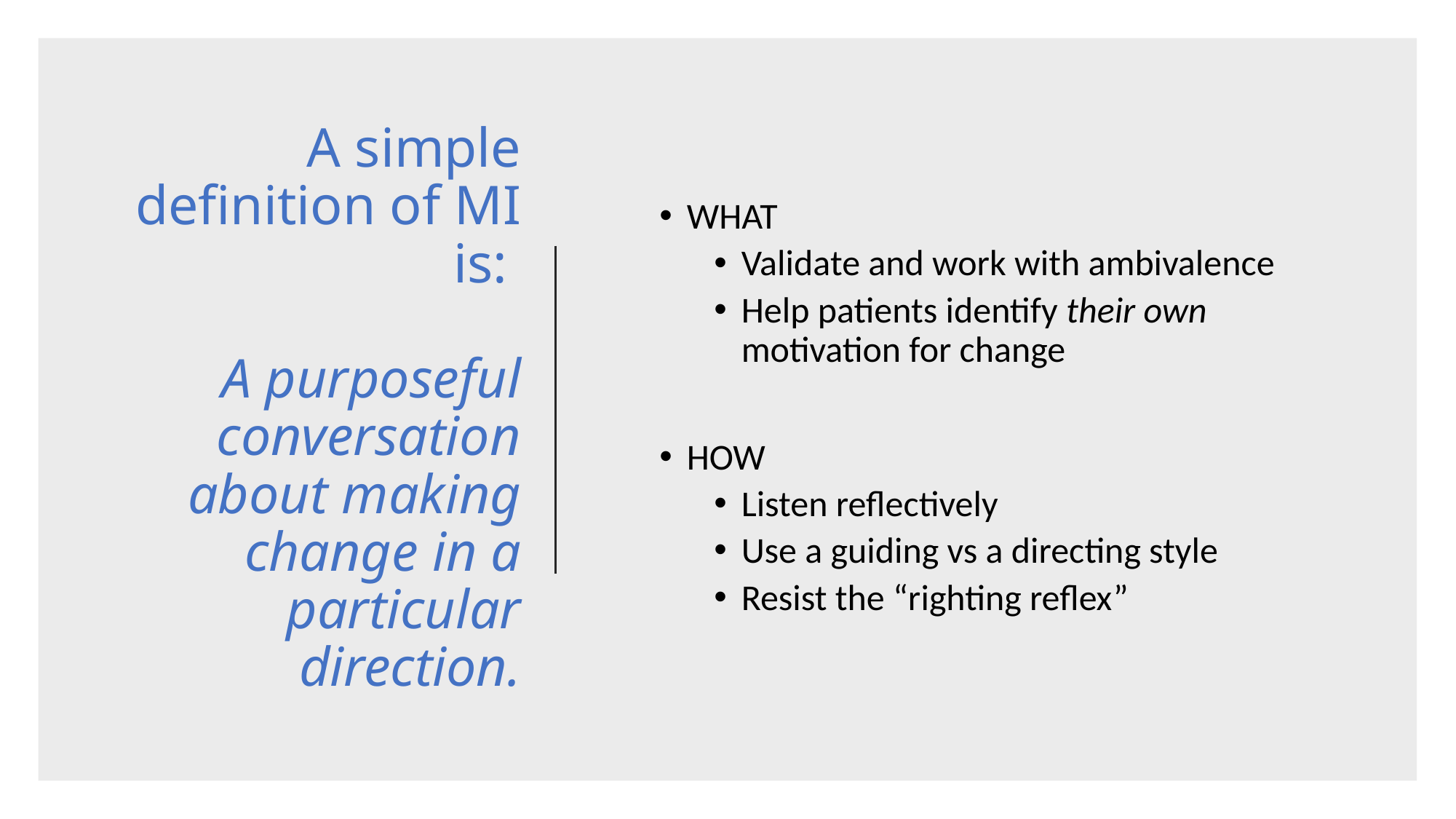

# A simple definition of MI is: A purposeful conversation about making change in a particular direction.
WHAT
Validate and work with ambivalence
Help patients identify their own motivation for change
HOW
Listen reflectively
Use a guiding vs a directing style
Resist the “righting reflex”

## Slide 11
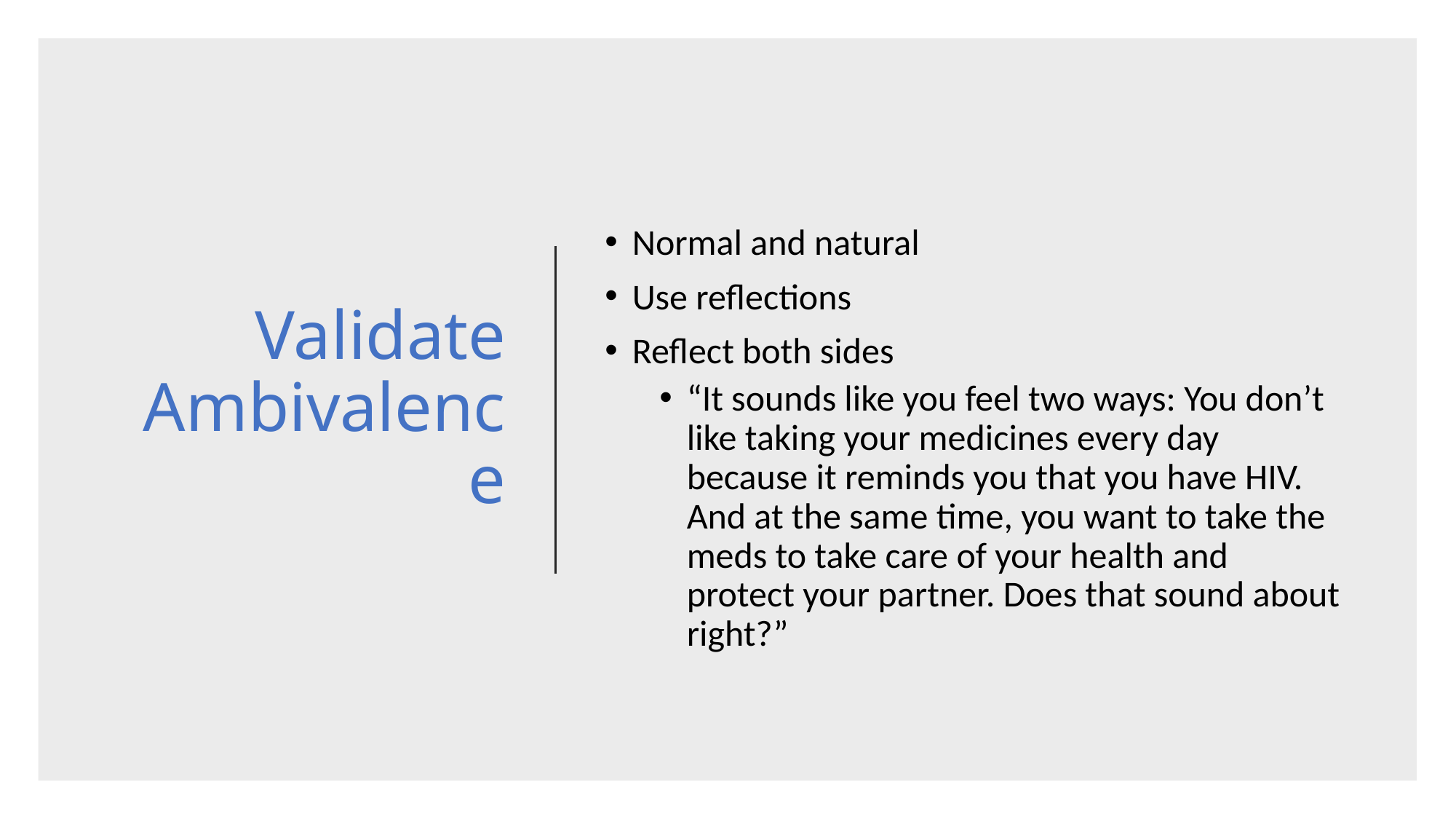

# Validate Ambivalence
Normal and natural
Use reflections
Reflect both sides
“It sounds like you feel two ways: You don’t like taking your medicines every day because it reminds you that you have HIV. And at the same time, you want to take the meds to take care of your health and protect your partner. Does that sound about right?”

## Slide 12
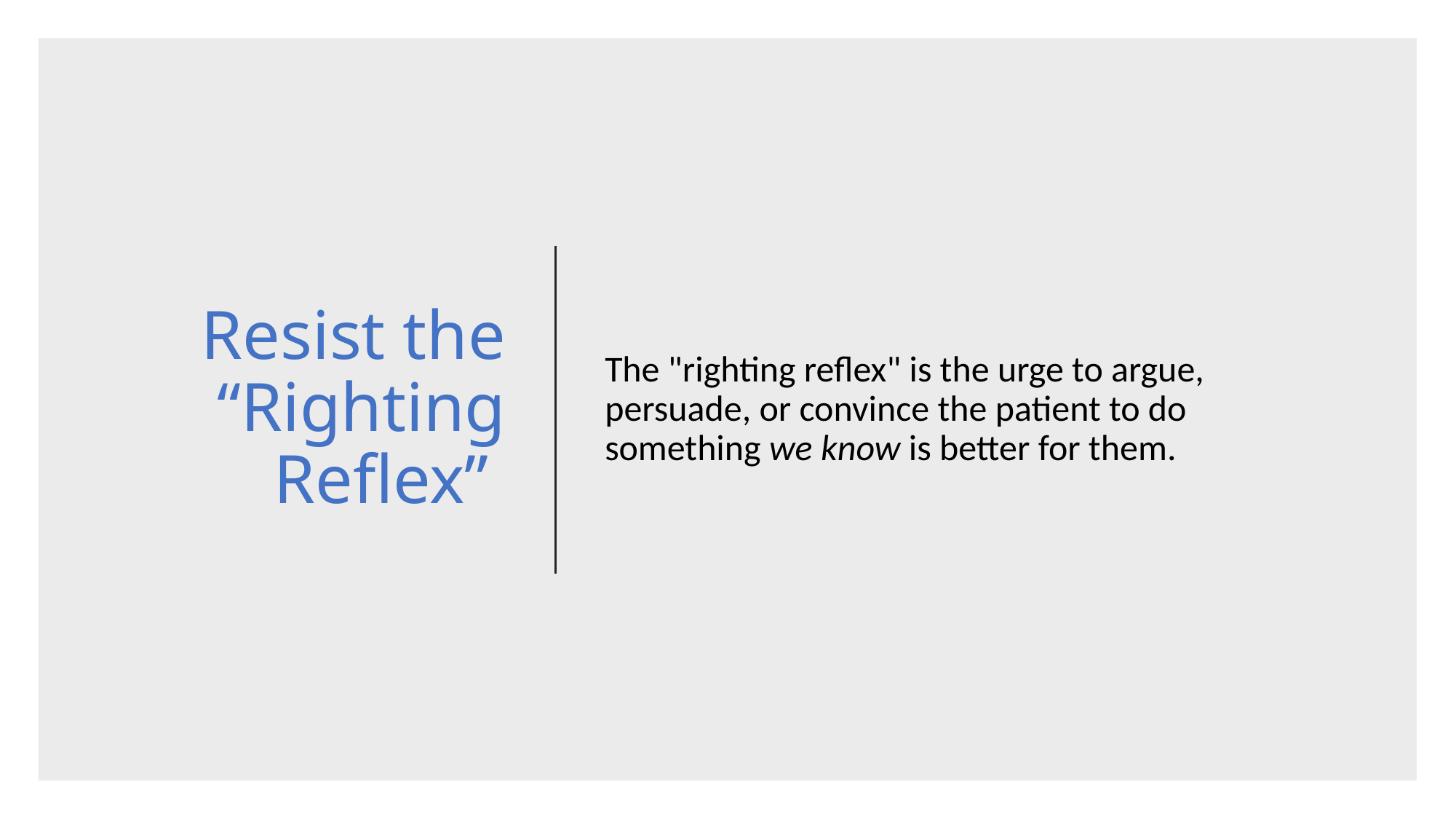

# Resist the “Righting Reflex”
The "righting reflex" is the urge to argue, persuade, or convince the patient to do something we know is better for them.

## Slide 13
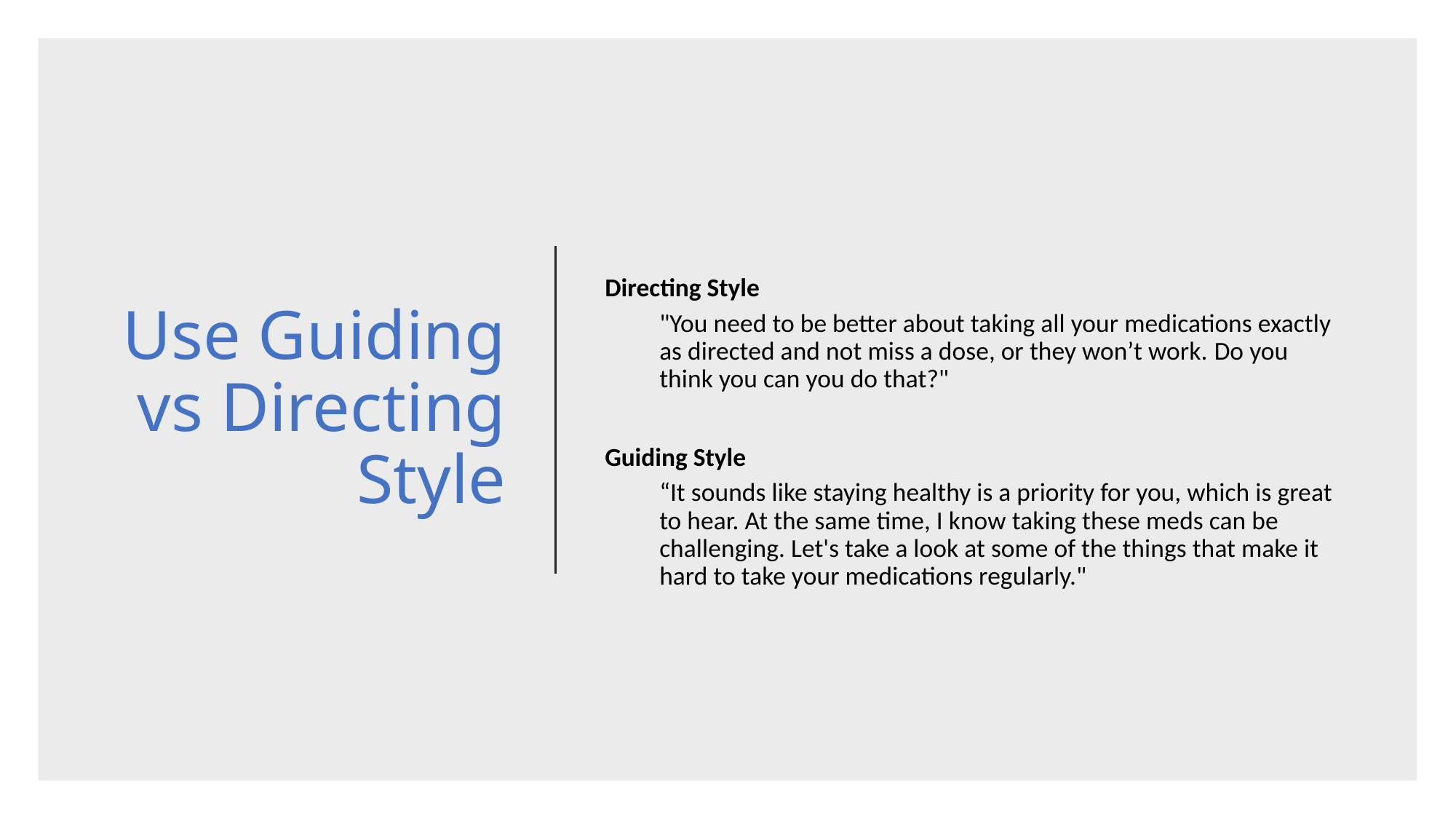

# Use Guiding vs Directing Style
Directing Style
"You need to be better about taking all your medications exactly as directed and not miss a dose, or they won’t work. Do you think you can you do that?"
Guiding Style
“It sounds like staying healthy is a priority for you, which is great to hear. At the same time, I know taking these meds can be challenging. Let's take a look at some of the things that make it hard to take your medications regularly."

## Slide 14
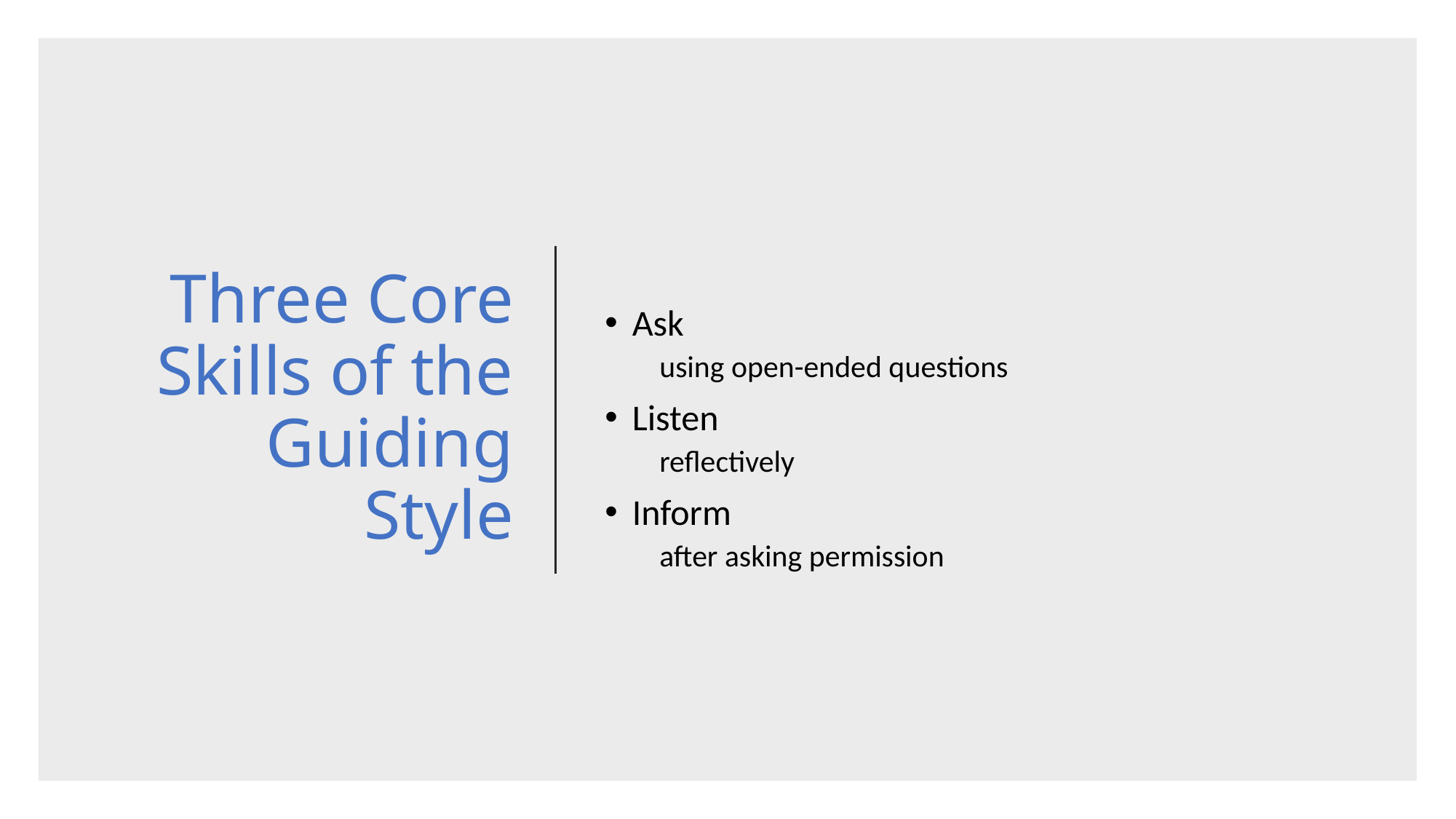

# Three Core Skills of the Guiding Style
Ask
using open-ended questions
Listen
reflectively
Inform
after asking permission

## Slide 15
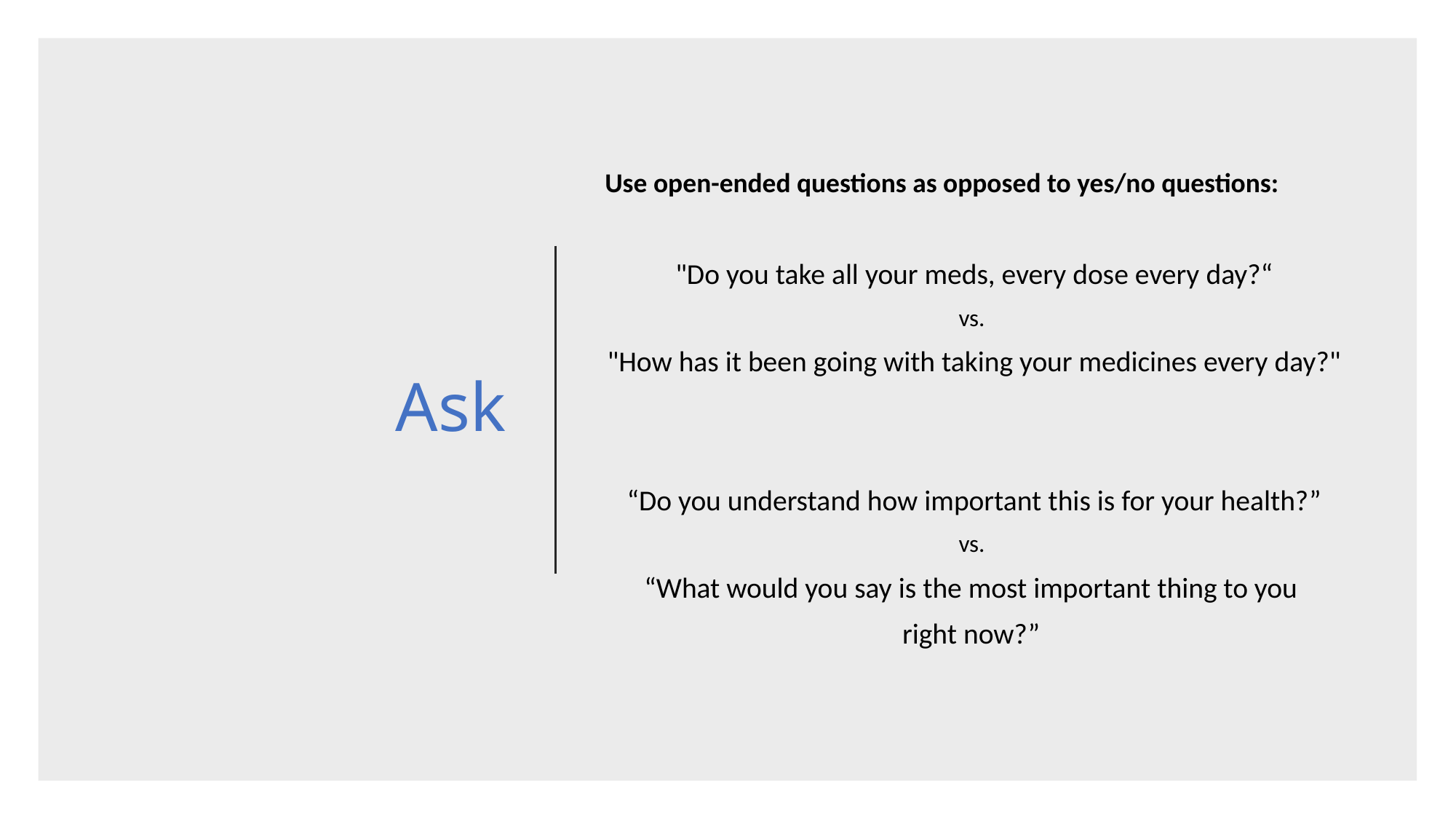

# Ask
Use open-ended questions as opposed to yes/no questions:
"Do you take all your meds, every dose every day?“
vs.
"How has it been going with taking your medicines every day?"
“Do you understand how important this is for your health?”
vs.
“What would you say is the most important thing to you
right now?”

## Slide 16
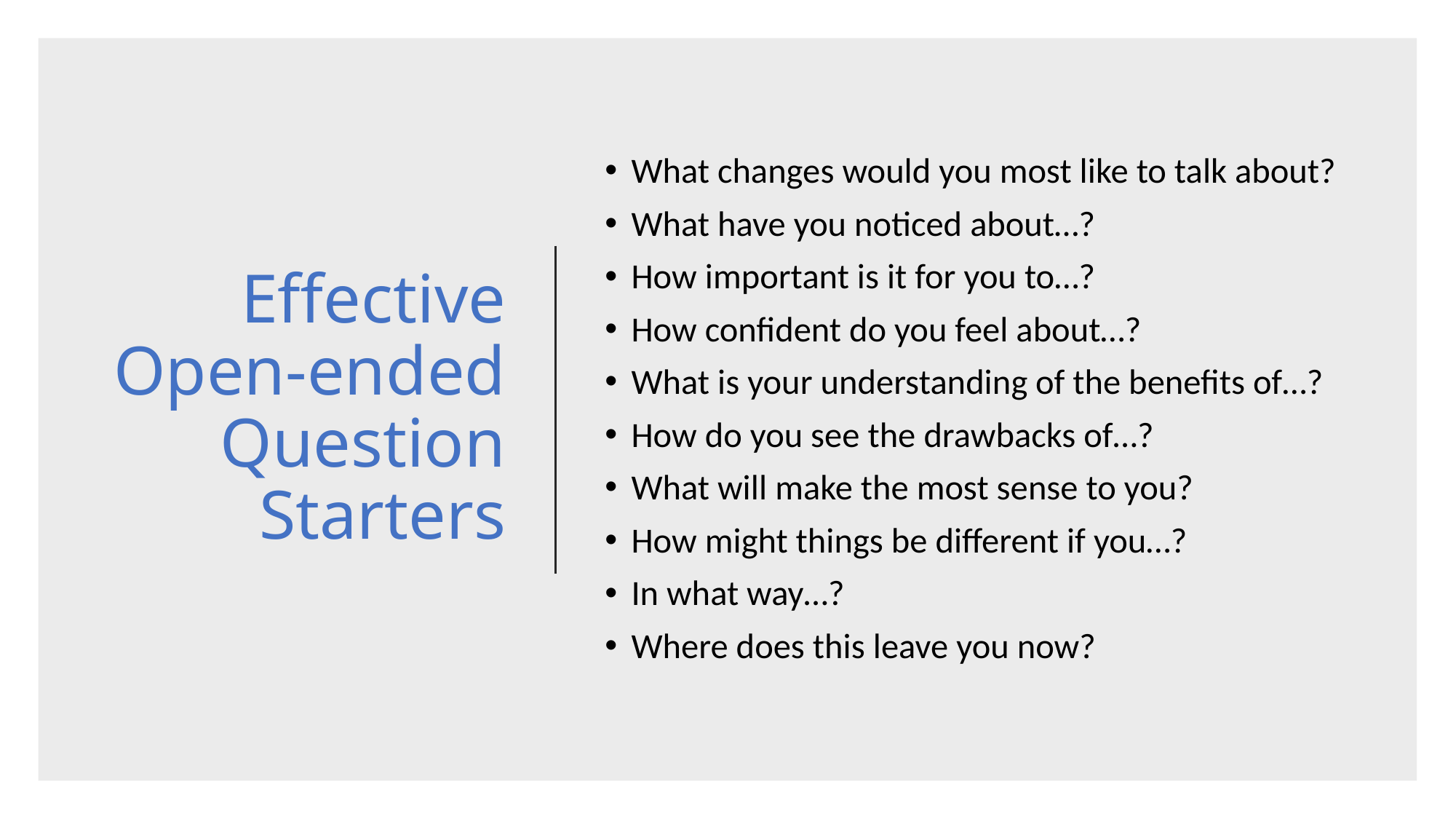

# Effective Open-ended Question Starters
What changes would you most like to talk about?
What have you noticed about…?
How important is it for you to…?
How confident do you feel about…?
What is your understanding of the benefits of…?
How do you see the drawbacks of…?
What will make the most sense to you?
How might things be different if you…?
In what way…?
Where does this leave you now?

## Slide 17
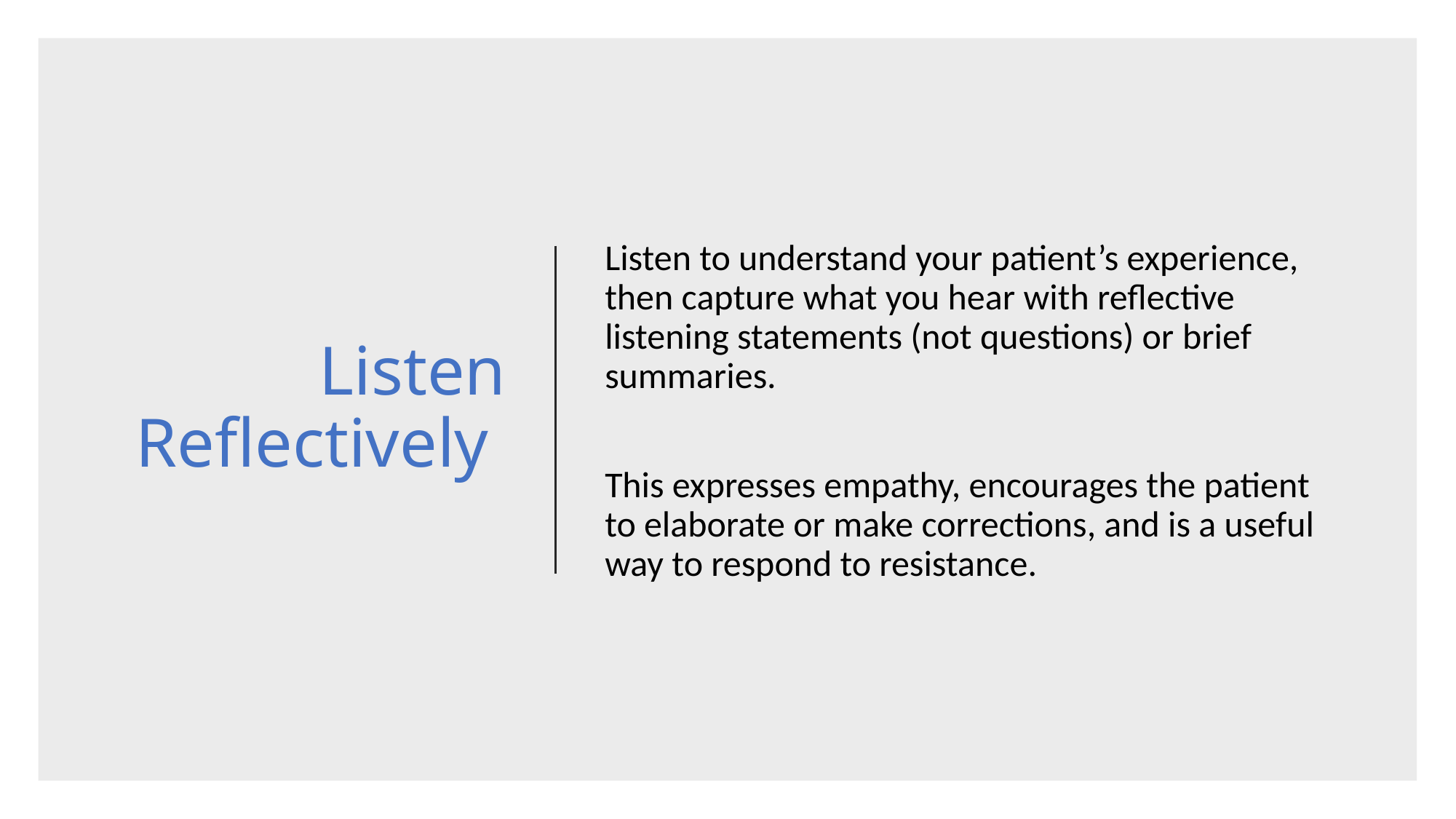

# Listen Reflectively
Listen to understand your patient’s experience, then capture what you hear with reflective listening statements (not questions) or brief summaries.
This expresses empathy, encourages the patient to elaborate or make corrections, and is a useful way to respond to resistance.

## Slide 18
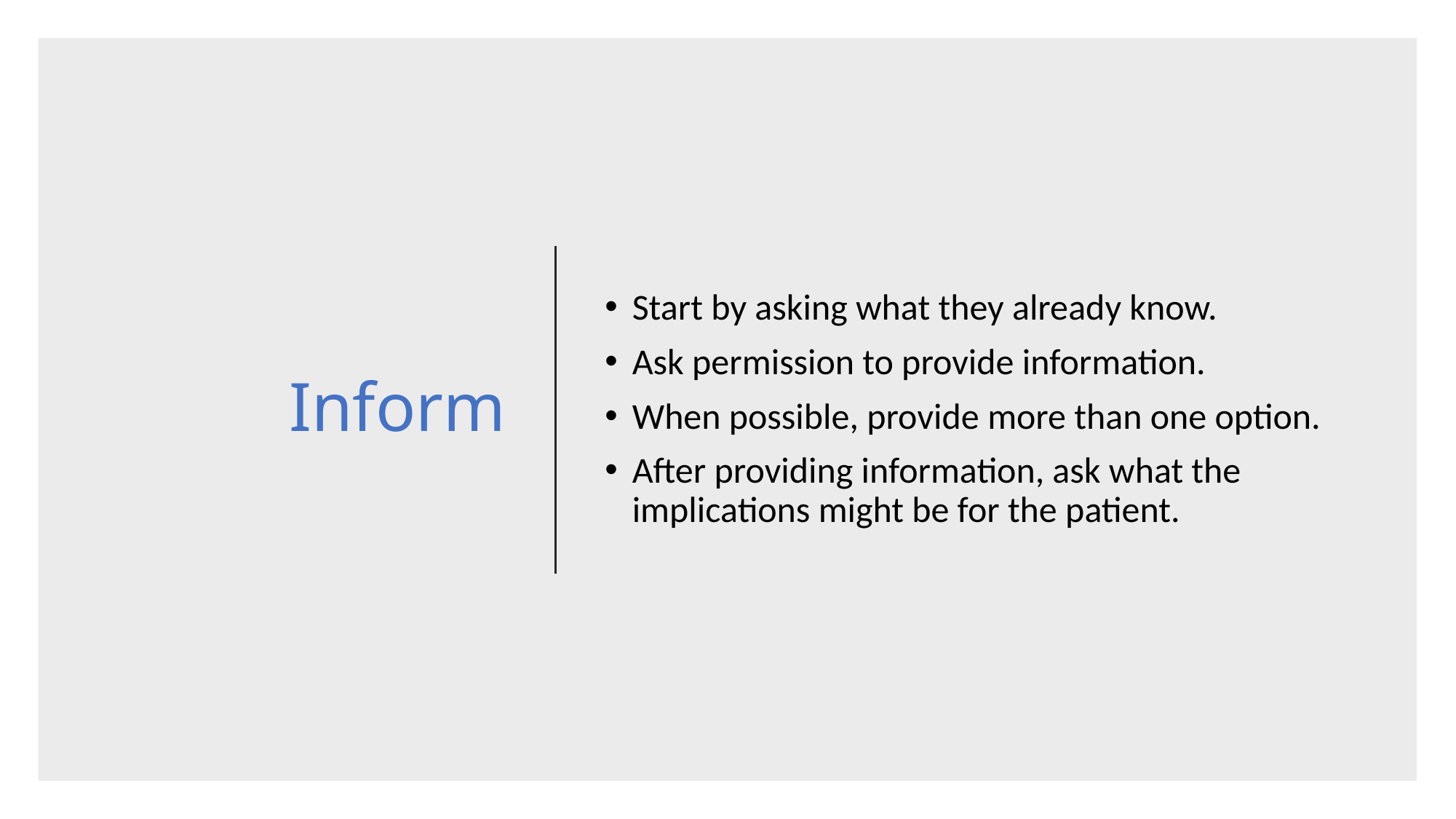

# Inform
Start by asking what they already know.
Ask permission to provide information.
When possible, provide more than one option.
After providing information, ask what the implications might be for the patient.

## Slide 19
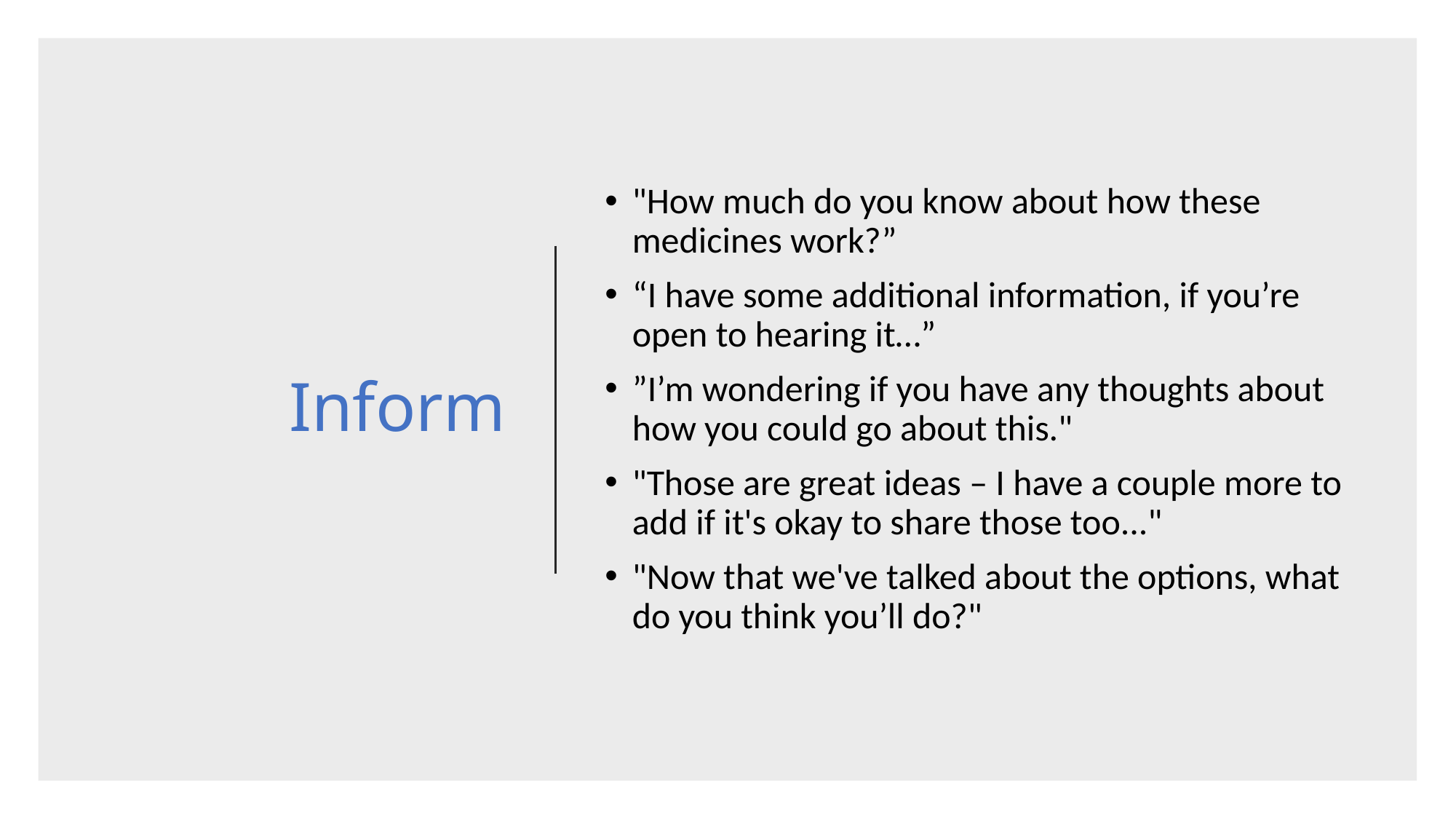

# Inform
"How much do you know about how these medicines work?”
“I have some additional information, if you’re open to hearing it…”
”I’m wondering if you have any thoughts about how you could go about this."
"Those are great ideas – I have a couple more to add if it's okay to share those too..."
"Now that we've talked about the options, what do you think you’ll do?"

## Slide 20
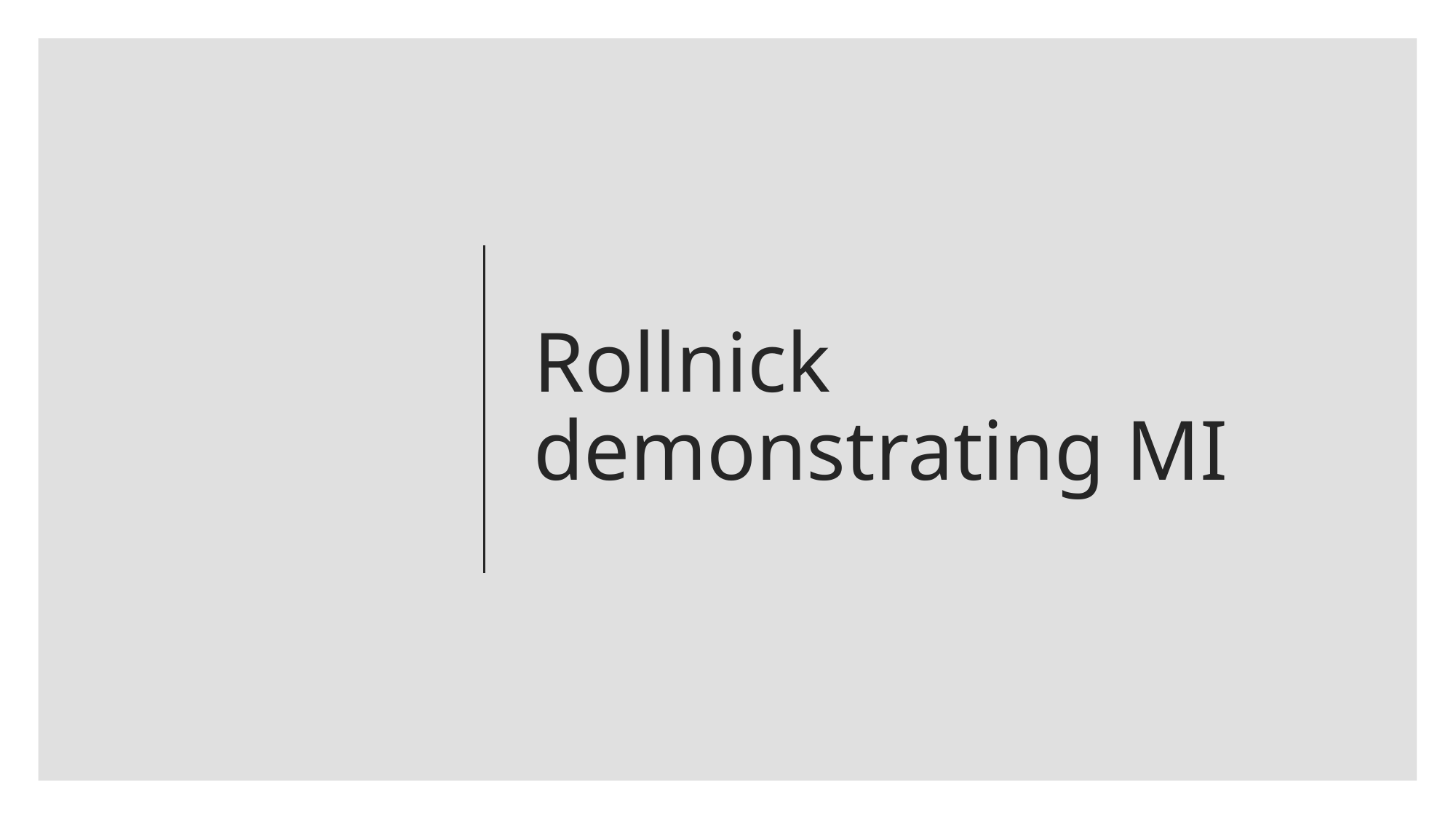

Rollnick demonstrating MI

## Slide 21
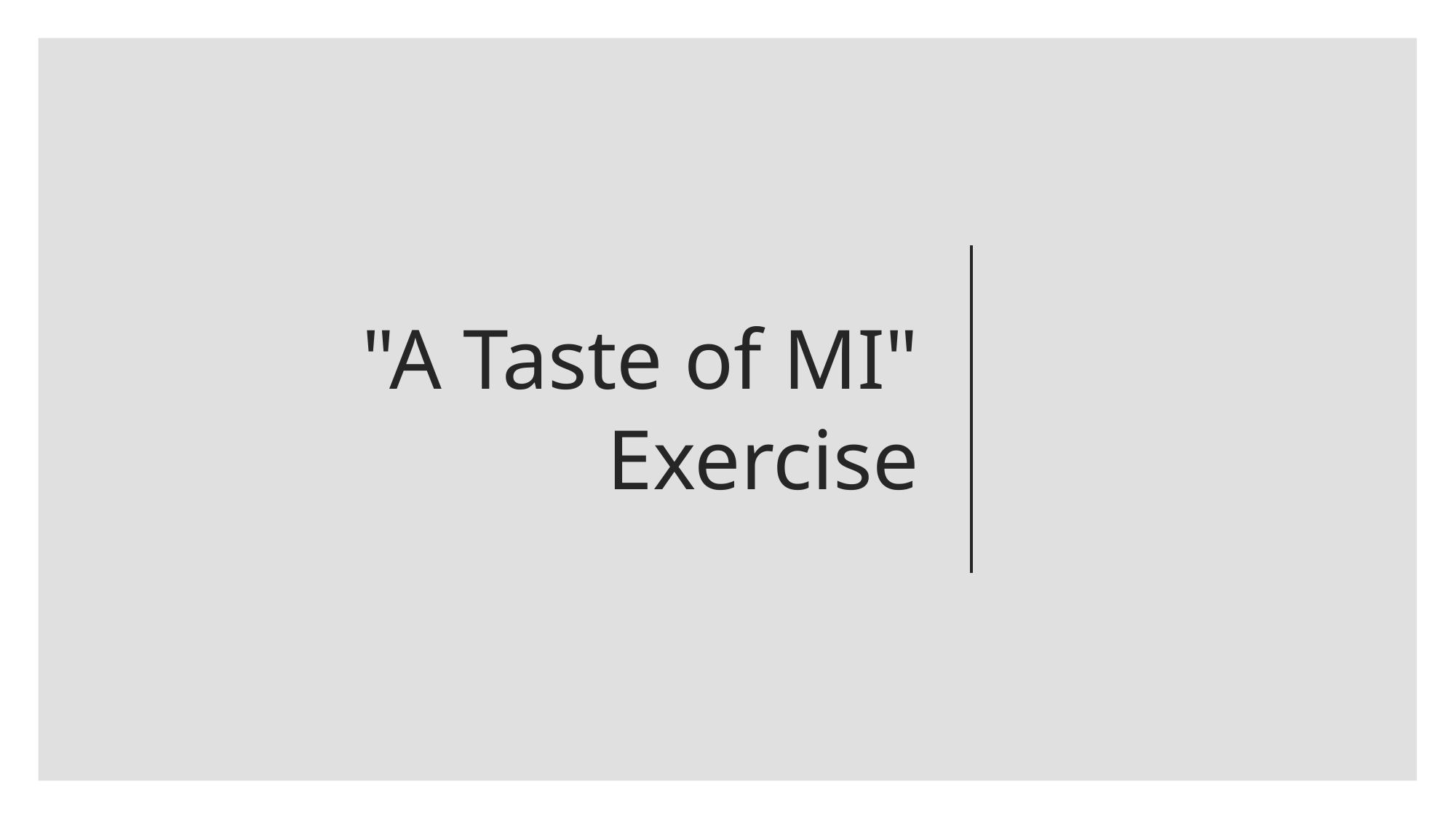

# "A Taste of MI" Exercise

## Slide 22
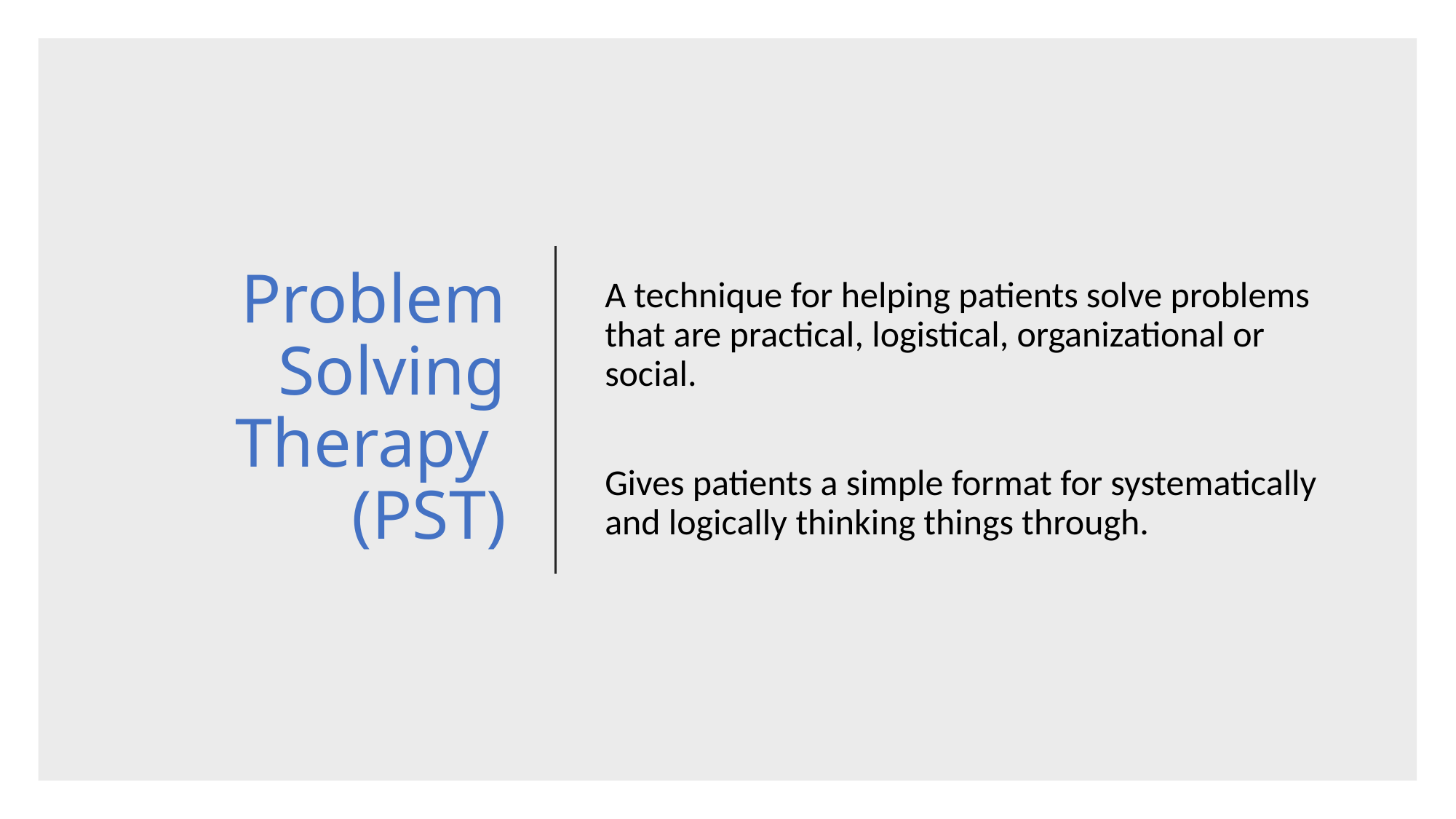

# Problem Solving Therapy (PST)
A technique for helping patients solve problems that are practical, logistical, organizational or social.
Gives patients a simple format for systematically and logically thinking things through.

## Slide 23
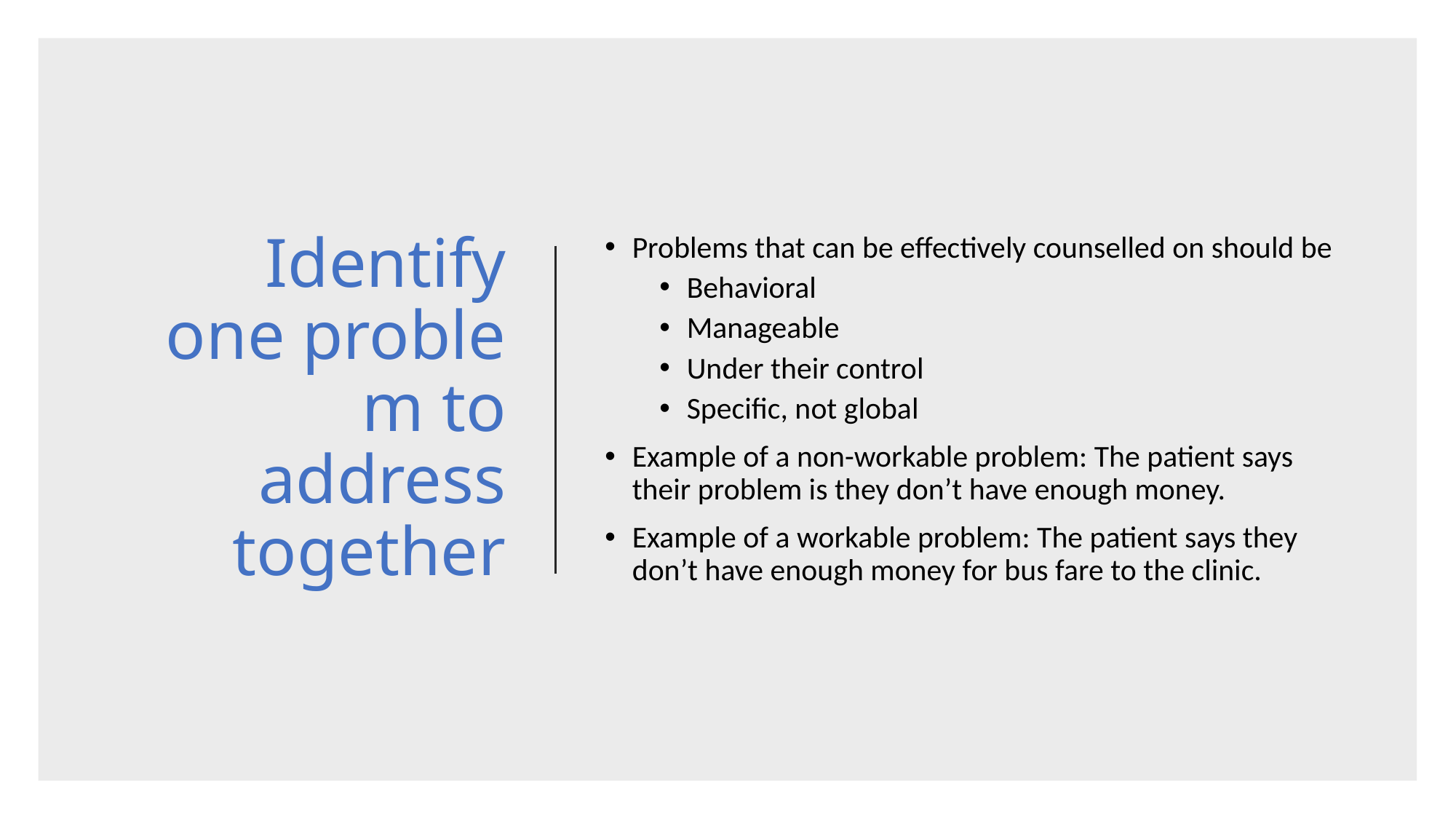

# Identify one problem to address together
Problems that can be effectively counselled on should be
Behavioral
Manageable
Under their control
Specific, not global
Example of a non-workable problem: The patient says their problem is they don’t have enough money.
Example of a workable problem: The patient says they don’t have enough money for bus fare to the clinic.

## Slide 24
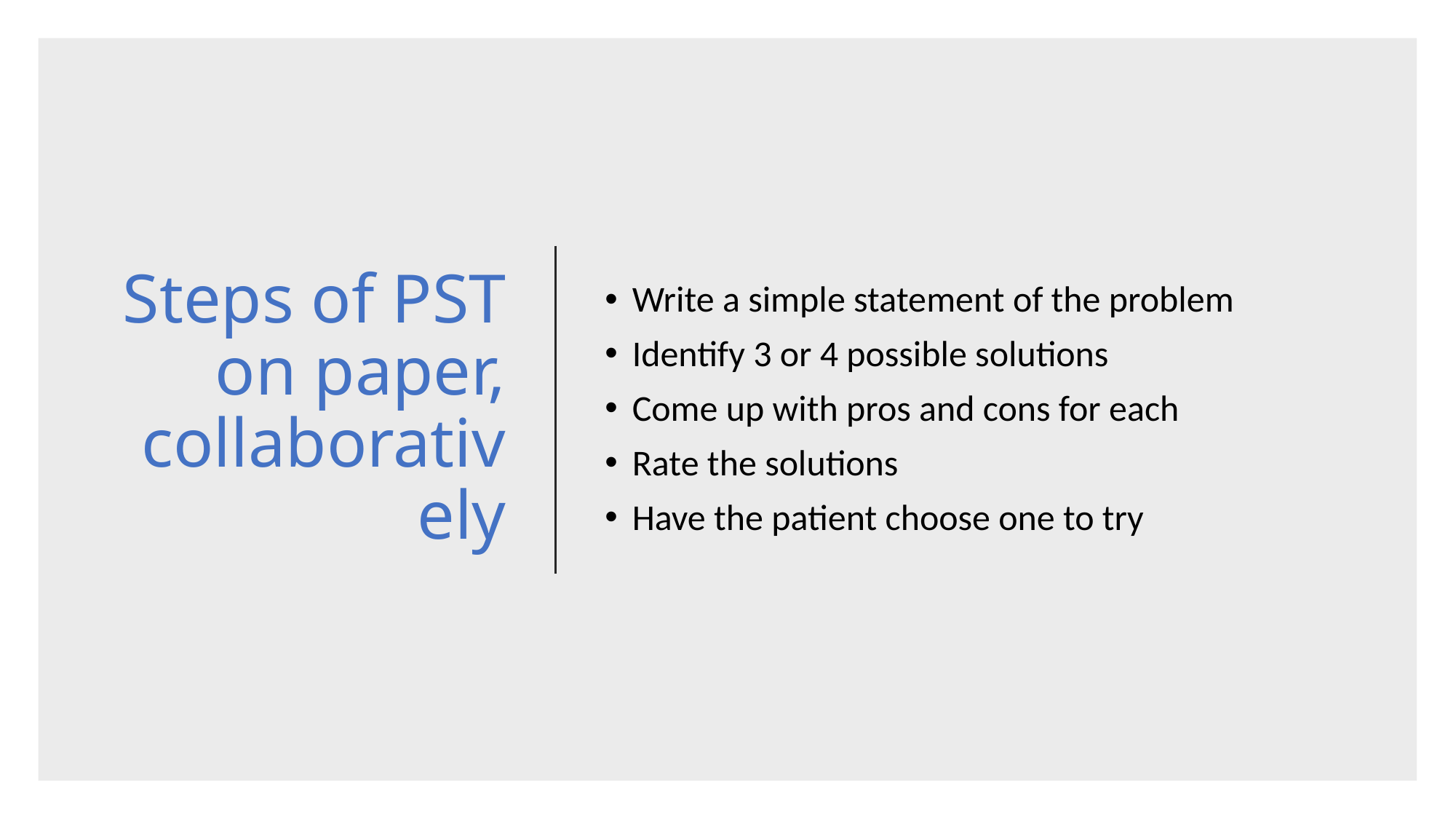

# Steps of PST on paper, collaboratively
Write a simple statement of the problem
Identify 3 or 4 possible solutions
Come up with pros and cons for each
Rate the solutions
Have the patient choose one to try

## Slide 25
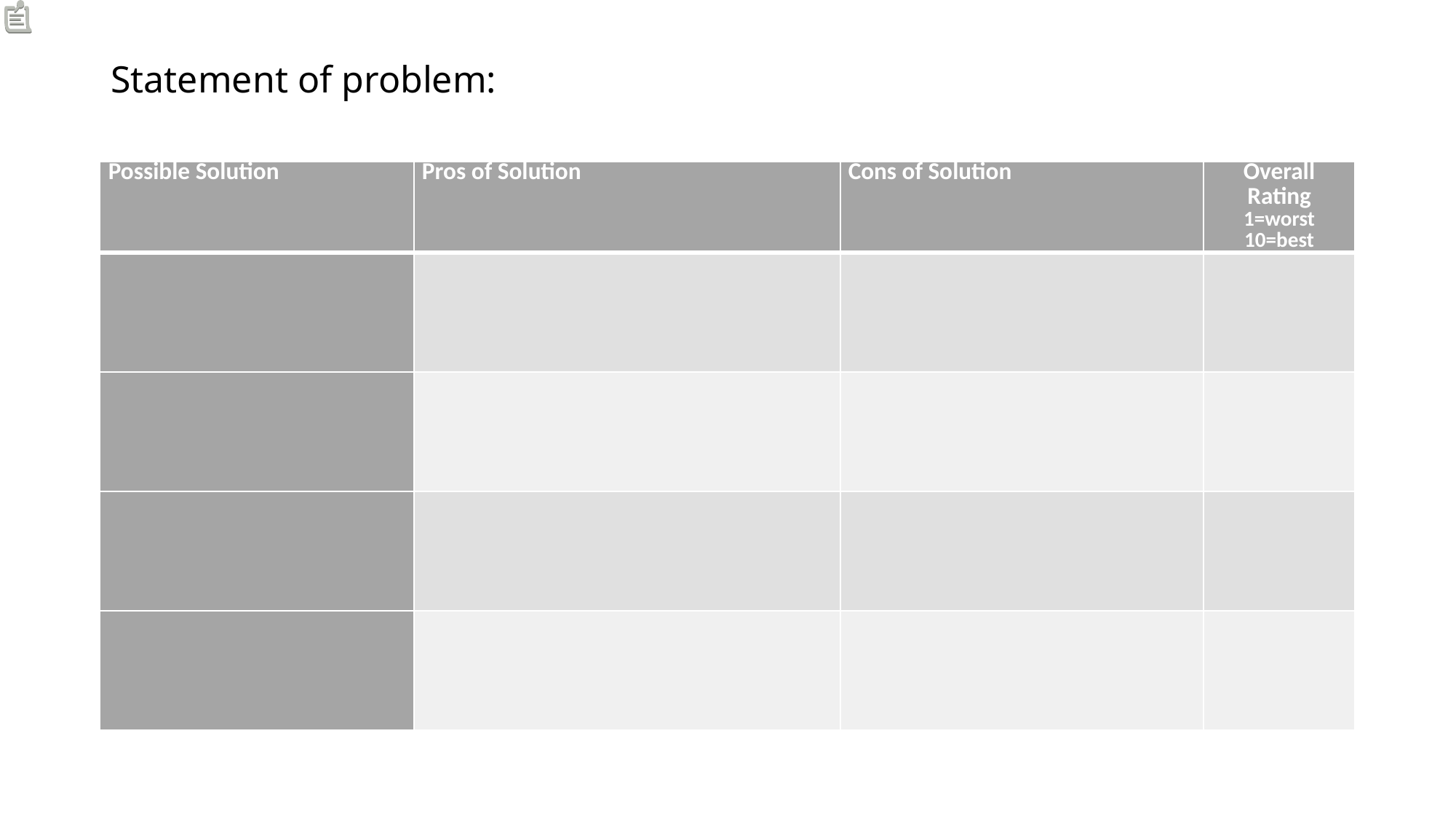

# Statement of problem:
| Possible Solution | Pros of Solution | Cons of Solution | Overall Rating 1=worst 10=best |
| --- | --- | --- | --- |
| | | | |
| | | | |
| | | | |
| | | | |

## Slide 26
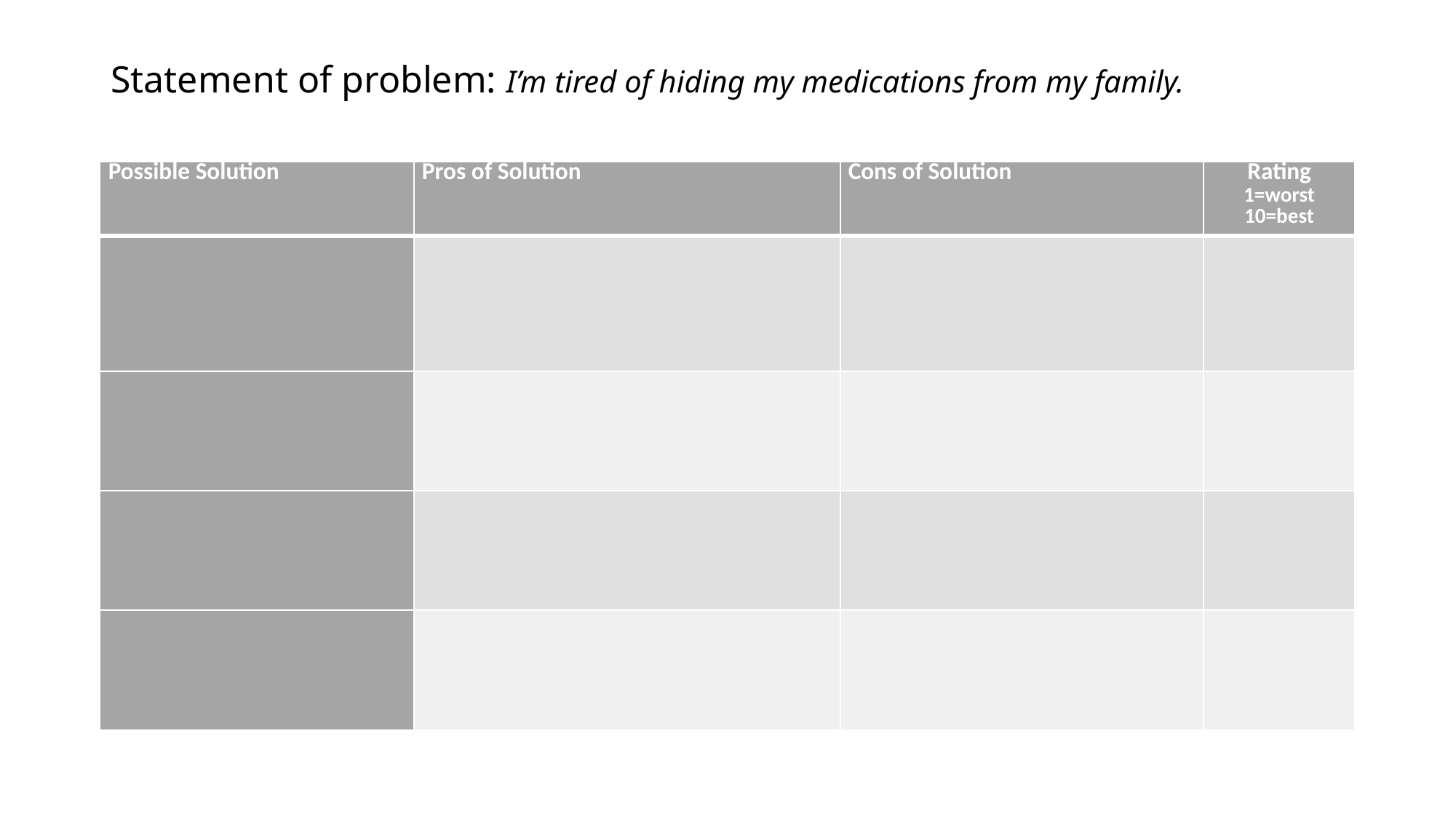

# Statement of problem: I’m tired of hiding my medications from my family.
| Possible Solution | Pros of Solution | Cons of Solution | Rating 1=worst 10=best |
| --- | --- | --- | --- |
| | | | |
| | | | |
| | | | |
| | | | |

## Slide 27
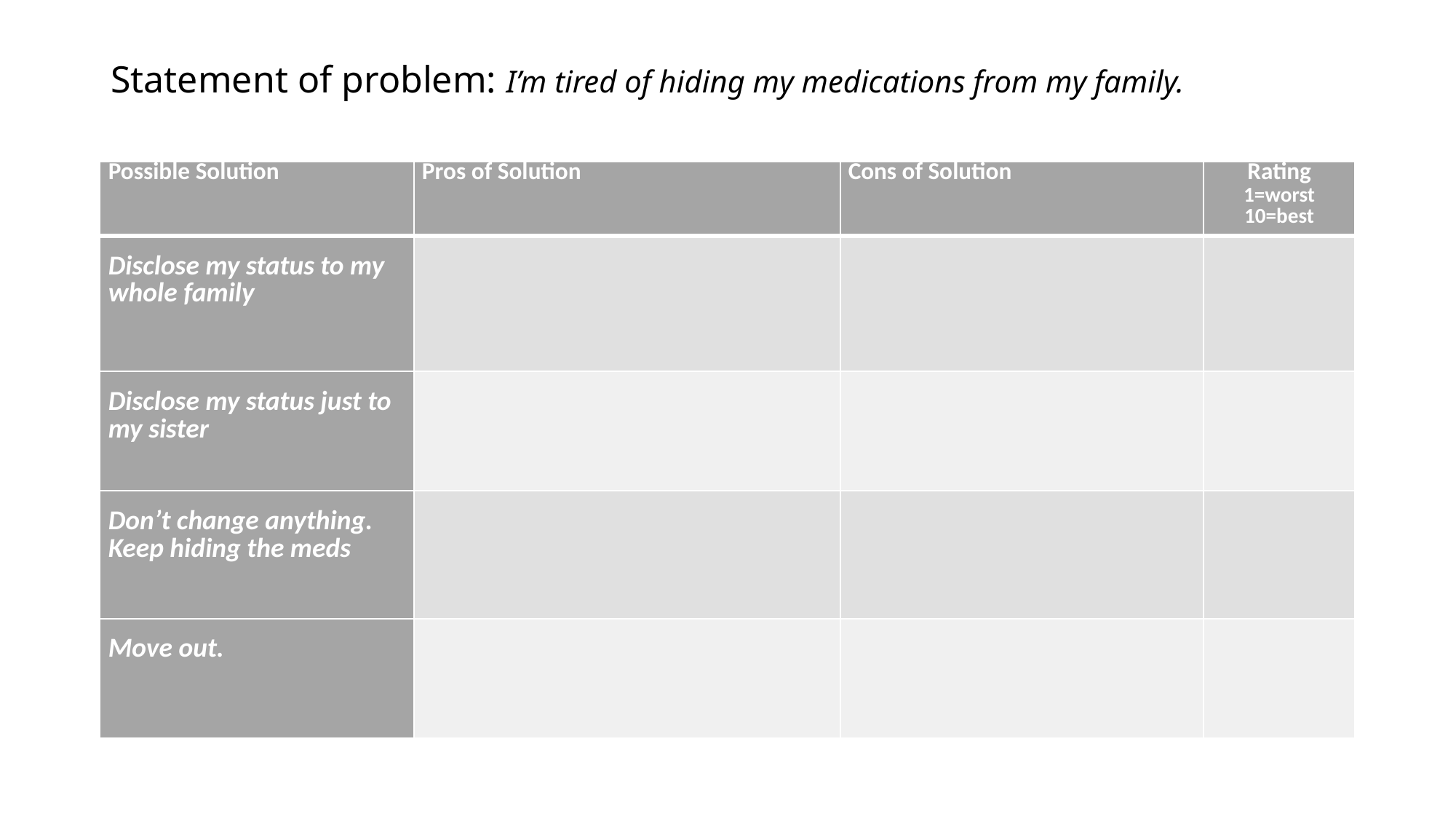

# Statement of problem: I’m tired of hiding my medications from my family.
| Possible Solution | Pros of Solution | Cons of Solution | Rating 1=worst 10=best |
| --- | --- | --- | --- |
| Disclose my status to my whole family | | | |
| Disclose my status just to my sister | | | |
| Don’t change anything. Keep hiding the meds | | | |
| Move out. | | | |

## Slide 28
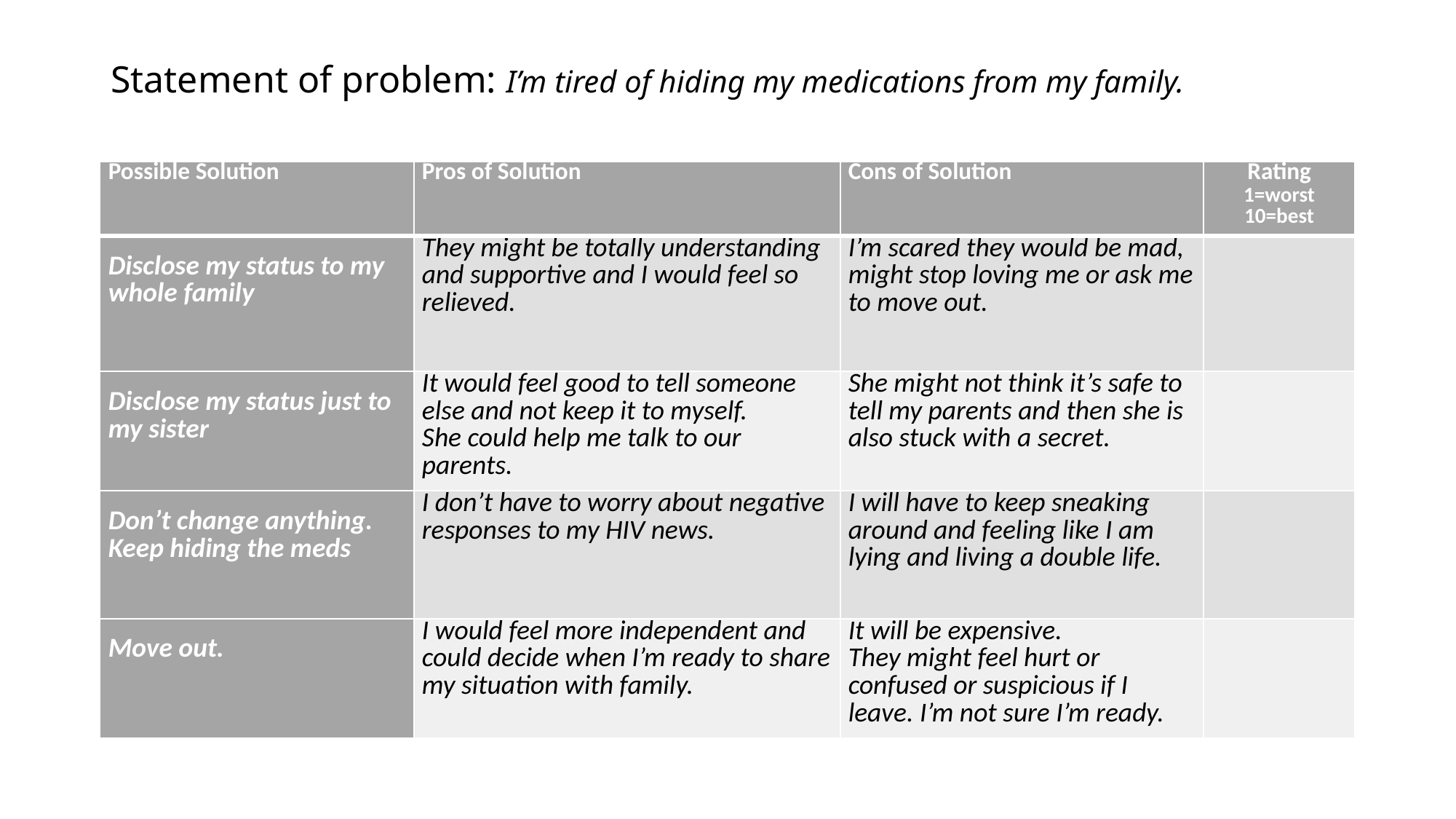

# Statement of problem: I’m tired of hiding my medications from my family.
| Possible Solution | Pros of Solution | Cons of Solution | Rating 1=worst 10=best |
| --- | --- | --- | --- |
| Disclose my status to my whole family | They might be totally understanding and supportive and I would feel so relieved. | I’m scared they would be mad, might stop loving me or ask me to move out. | |
| Disclose my status just to my sister | It would feel good to tell someone else and not keep it to myself. She could help me talk to our parents. | She might not think it’s safe to tell my parents and then she is also stuck with a secret. | |
| Don’t change anything. Keep hiding the meds | I don’t have to worry about negative responses to my HIV news. | I will have to keep sneaking around and feeling like I am lying and living a double life. | |
| Move out. | I would feel more independent and could decide when I’m ready to share my situation with family. | It will be expensive. They might feel hurt or confused or suspicious if I leave. I’m not sure I’m ready. | |

## Slide 29
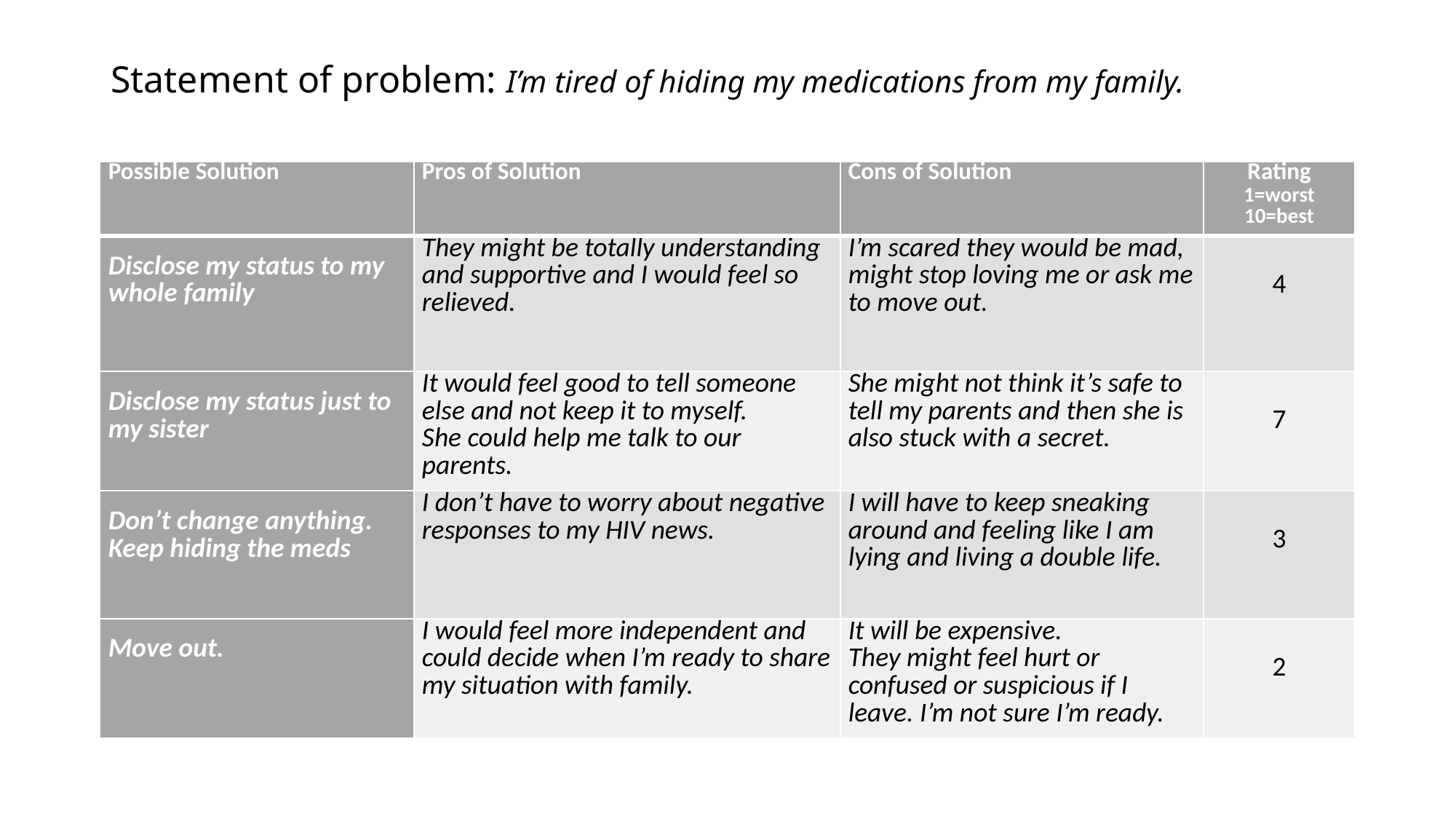

# Statement of problem: I’m tired of hiding my medications from my family.
| Possible Solution | Pros of Solution | Cons of Solution | Rating 1=worst 10=best |
| --- | --- | --- | --- |
| Disclose my status to my whole family | They might be totally understanding and supportive and I would feel so relieved. | I’m scared they would be mad, might stop loving me or ask me to move out. | 4 |
| Disclose my status just to my sister | It would feel good to tell someone else and not keep it to myself. She could help me talk to our parents. | She might not think it’s safe to tell my parents and then she is also stuck with a secret. | 7 |
| Don’t change anything. Keep hiding the meds | I don’t have to worry about negative responses to my HIV news. | I will have to keep sneaking around and feeling like I am lying and living a double life. | 3 |
| Move out. | I would feel more independent and could decide when I’m ready to share my situation with family. | It will be expensive. They might feel hurt or confused or suspicious if I leave. I’m not sure I’m ready. | 2 |

## Slide 30
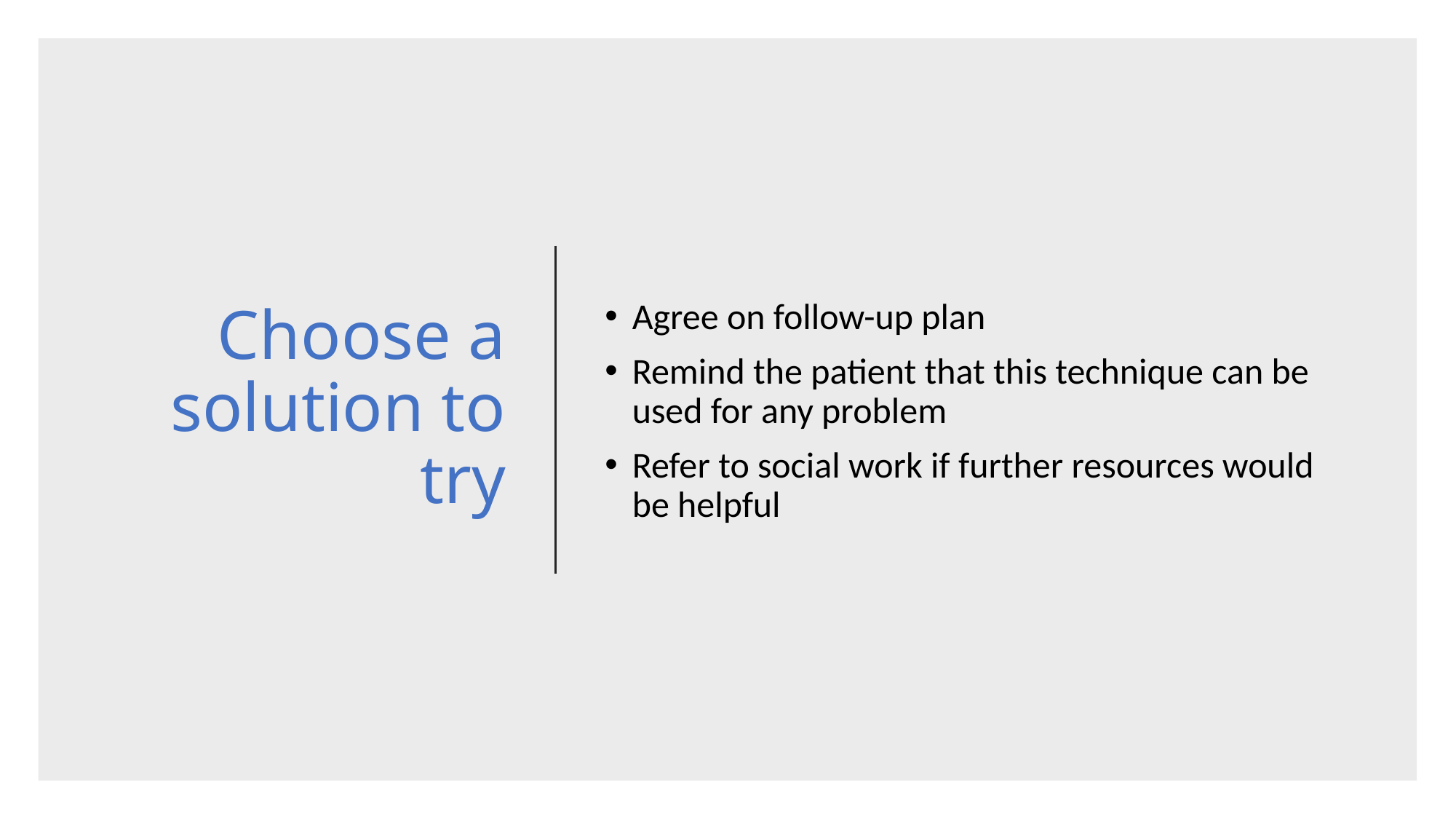

# Choose a solution to try
Agree on follow-up plan
Remind the patient that this technique can be used for any problem
Refer to social work if further resources would be helpful

## Slide 31
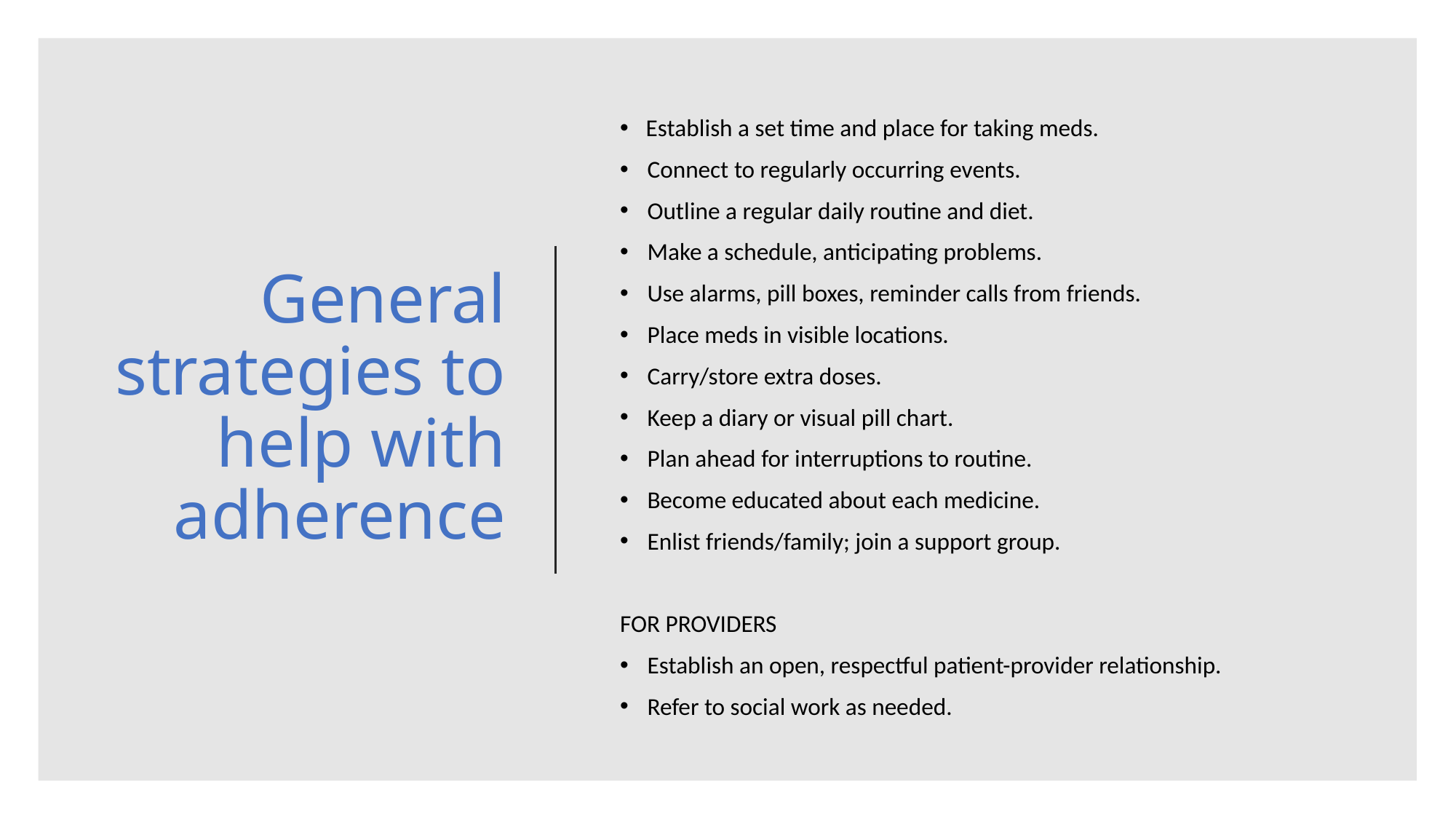

# General strategies to help with adherence
 Establish a set time and place for taking meds.
Connect to regularly occurring events.
Outline a regular daily routine and diet.
Make a schedule, anticipating problems.
Use alarms, pill boxes, reminder calls from friends.
Place meds in visible locations.
Carry/store extra doses.
Keep a diary or visual pill chart.
Plan ahead for interruptions to routine.
Become educated about each medicine.
Enlist friends/family; join a support group.
FOR PROVIDERS
Establish an open, respectful patient-provider relationship.
Refer to social work as needed.

## Slide 32
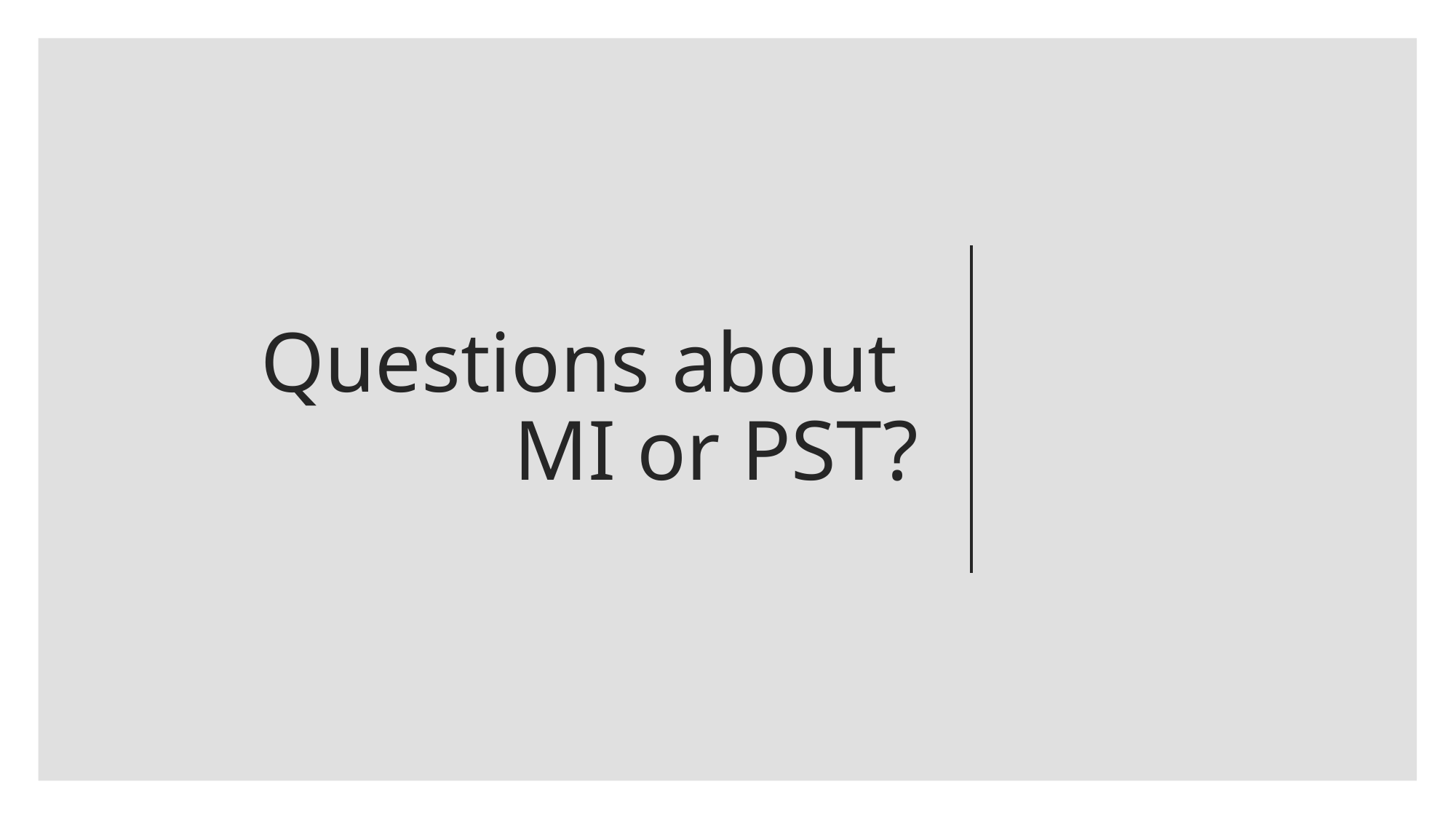

# Questions about MI or PST?

## Slide 33
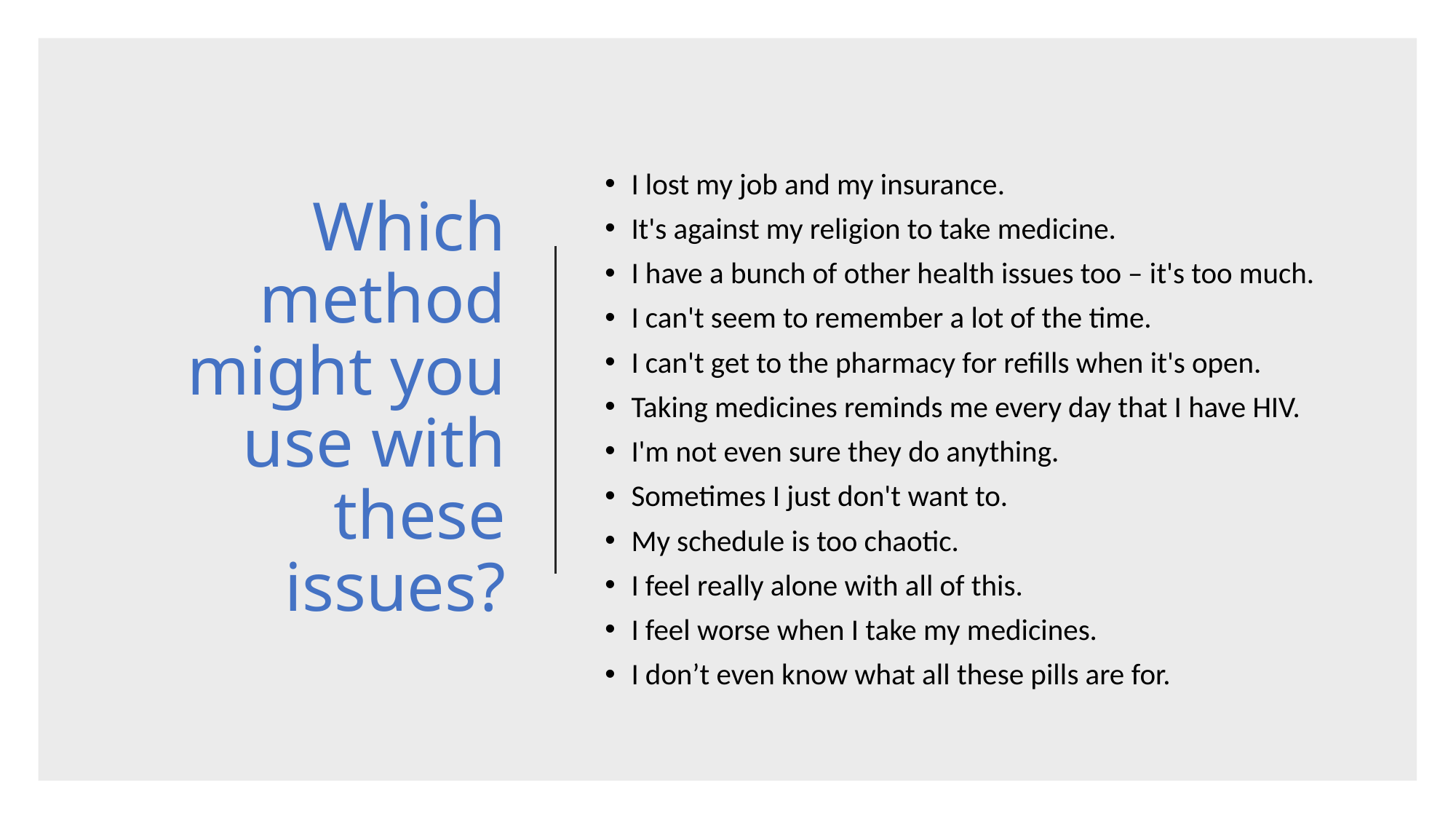

# Which method might you use with these issues?
I lost my job and my insurance.
It's against my religion to take medicine.
I have a bunch of other health issues too – it's too much.
I can't seem to remember a lot of the time.
I can't get to the pharmacy for refills when it's open.
Taking medicines reminds me every day that I have HIV.
I'm not even sure they do anything.
Sometimes I just don't want to.
My schedule is too chaotic.
I feel really alone with all of this.
I feel worse when I take my medicines.
I don’t even know what all these pills are for.

## Slide 34
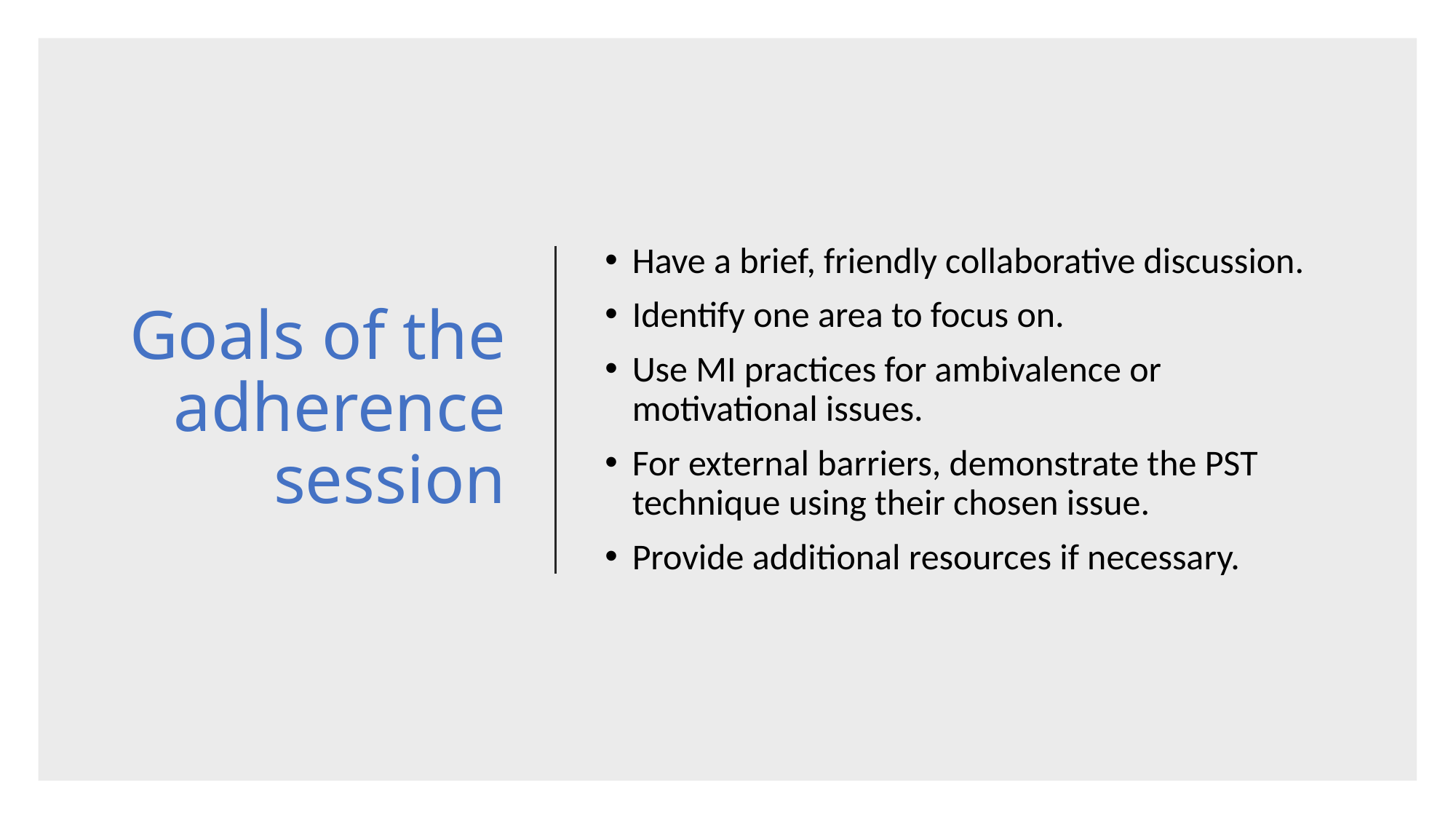

# Goals of the adherence session
Have a brief, friendly collaborative discussion.
Identify one area to focus on.
Use MI practices for ambivalence or motivational issues.
For external barriers, demonstrate the PST technique using their chosen issue.
Provide additional resources if necessary.

## Slide 35
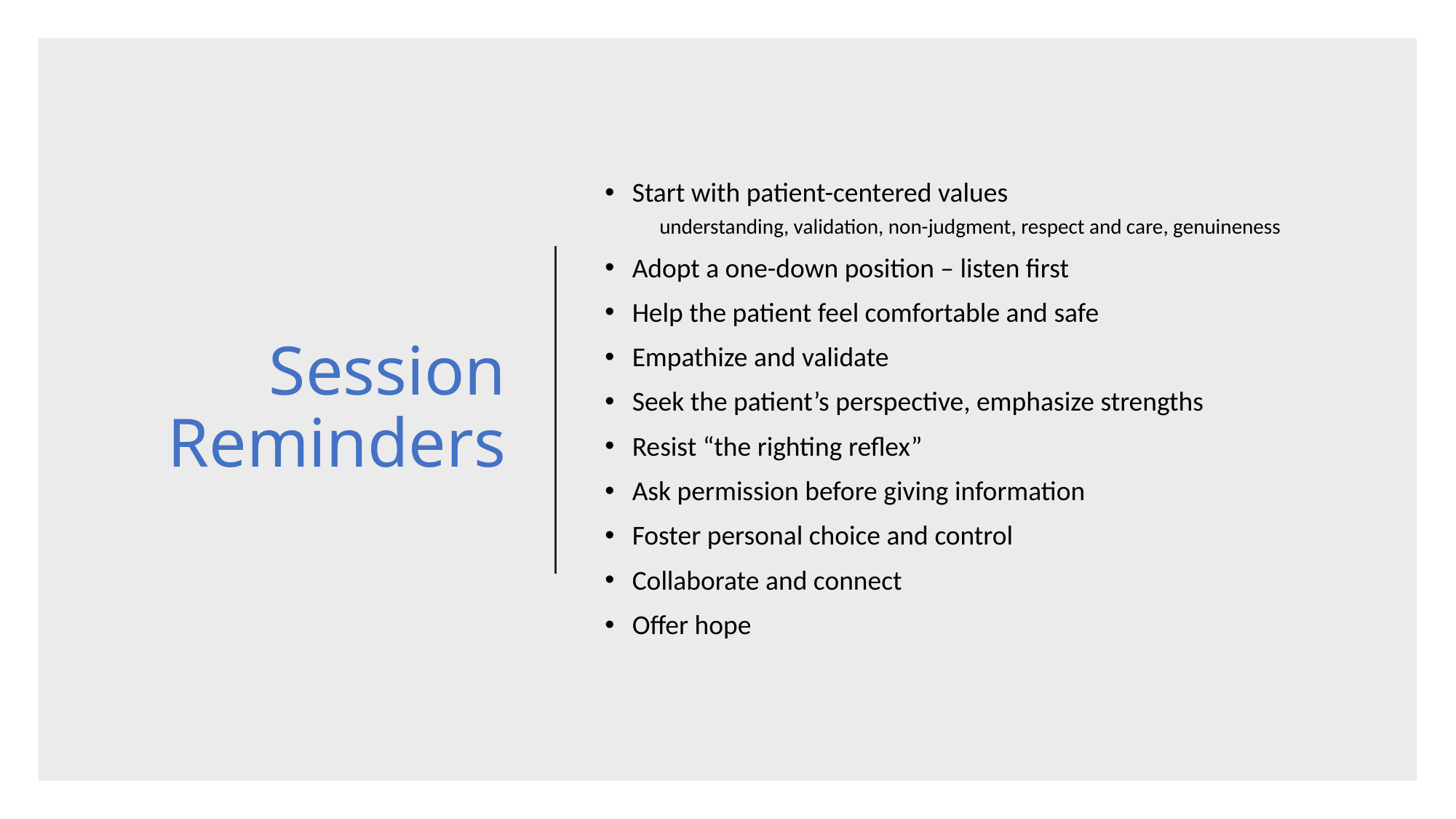

# Session Reminders
Start with patient-centered values
understanding, validation, non-judgment, respect and care, genuineness
Adopt a one-down position – listen first
Help the patient feel comfortable and safe
Empathize and validate
Seek the patient’s perspective, emphasize strengths
Resist “the righting reflex”
Ask permission before giving information
Foster personal choice and control
Collaborate and connect
Offer hope

## Slide 36
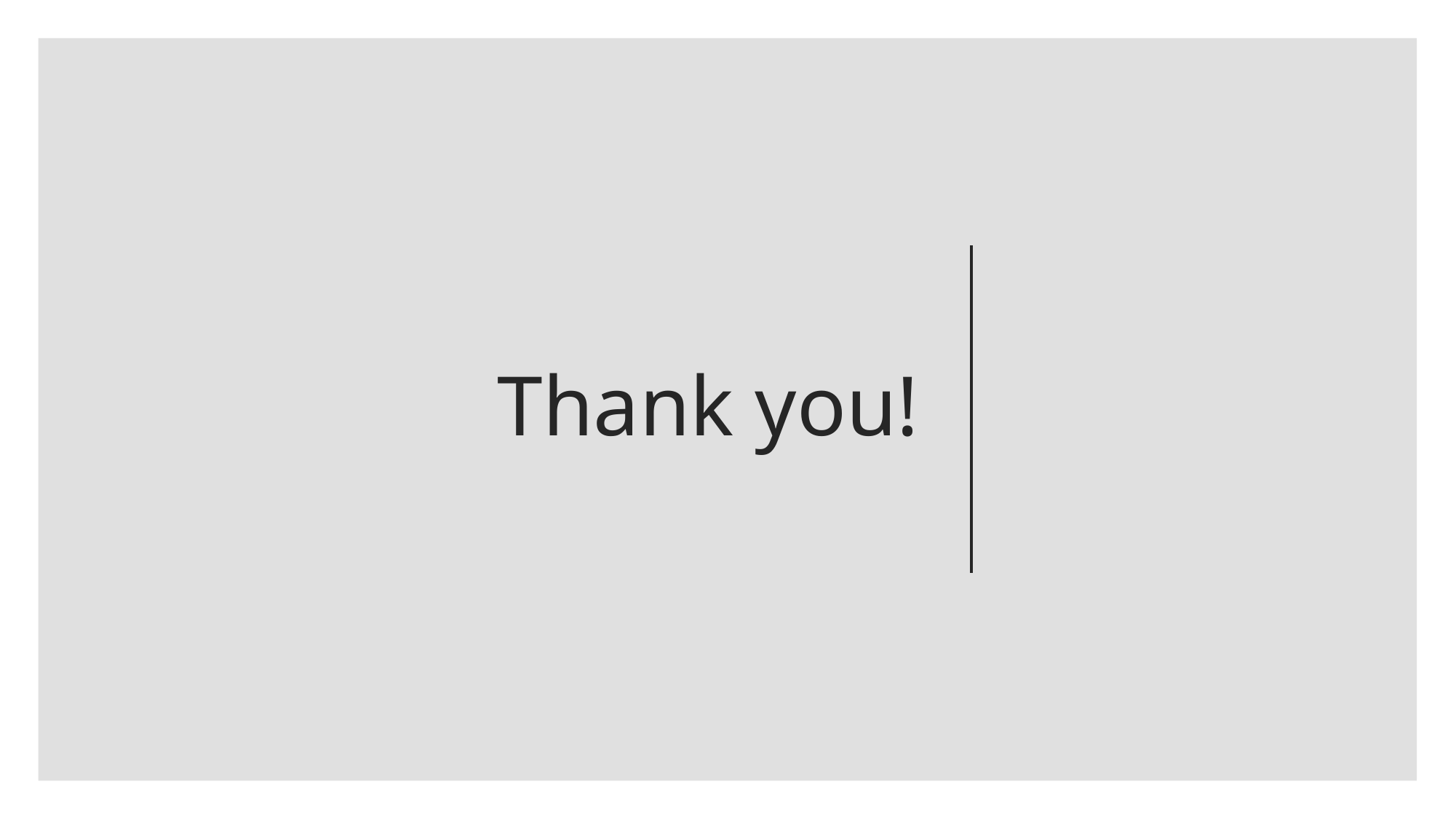

Thank you!
