# Supplementary material for: Feasibility, Acceptability, and Test Performance of Point-of-Care Nucleic Acid Tests for HIV Testing and Viral Load Monitoring in the United States: Prospective Longitudinal Mixed-Methods Study
Source: JMIR Res Protoc. 2026 Jul 23;15:e84625. doi: 10.2196/84625 (PMC13395423; doi:10.2196/84625)
Supplement: Multimedia Appendix 2 [file resprot-v15-e84625-s002.pdf]

"A Taste of MI" Exercise

Invite someone to have a brief conversation (about 10 minutes) with you about a change they might want to make. The speaker's topic is:

> "Something about yourself that you want to change, have been thinking about changing, need to change, or know that you ought to change. It might be something that you would increase (for example, physical activity or exercise, healthy eating, flossing teeth, cleaning up a room or garage, or sleeping). It might be something that you would *decrease* (for example, computer or TV time, drinking coffee, criticizing others, biting fingernails, or eating sweets). It doesn't have to be a 'problem.' It might be an opportunity. The point is that you have been considering making this change, and haven't done it yet."

As the interviewer, listen carefully with a goal of *understanding* the person's dilemma. Don't try to fix it. Give no advice. Instead, just find out what change the person is considering, and then ask these four open questions, listening carefully to the answers:

1. *Why would you want to make this change?*
2. *If you did decide to do it, how might you go about it in order to succeed?*
3. *What would you say are the three best reasons for you to do it?*
4. *On a scale from zero to ten, how important would you say it is for you to make this change, if 0 means "not at all important" and 10 means "the most important thing in my life right now"? What number would you say?*

4a. Then, when you hear the number, ask: *And why are you at ___ and not zero?* If you have reflective listening skills, use them as the person answers each question, reflecting what was said and remembering it.

When you have asked all four questions and listened well to the answers, offer the person a *summary* of what you heard, of the person's own motivations for making this change. Why do they want to make this change? How might they do it? What are their good reasons for doing it? Why is it important?

And then ask one more question:

5. *"So what do you think you'll do?"*

and just listen with interest and curiosity.
