# Supplementary material for: Feasibility, Acceptability, and Test Performance of Point-of-Care Nucleic Acid Tests for HIV Testing and Viral Load Monitoring in the United States: Prospective Longitudinal Mixed-Methods Study
Source: JMIR Res Protoc. 2026 Jul 23;15:e84625. doi: 10.2196/84625 (PMC13395423; doi:10.2196/84625)
Supplement: Multimedia Appendix 3 [file resprot-v15-e84625-s003.docx]

**GAIN Study CASI Patient Acceptability Survey: HIV Testing participants at Seattle’s LGBTQ+ Center (formerly known as Gay City) and Madison Clinic**

We are asking you to complete this survey because you recently participated in the GAIN study. We want to understand your experience with getting the point-of-care nucleic acid test (POC NAT). We will ask you questions about yourself, your participation in the study, and your experience with the POC NAT used at your recent visit.

Considering that some of the questions may be about sensitive topics, we suggest taking this survey in a private location.

This survey is completely voluntary, and you may stop at any time. We expect this survey to take about 20 minutes. After you finish the survey, we will send you a $10 gift card for your time.

Please do not use your browser’s back button. If you do, you might have to restart the survey from the beginning. Instead, please use the “Previous Page” button if you need to go back to an earlier question.

If you have any questions or concerns, please contact Joanne Stekler (206-744-8312) or email our study team at GainStudy@uw.edu.

Thank you for your participation!

**<PAGE BREAK>**

Please let us know your thoughts on the POC NAT that you had at your study visit:

1. Why did you come in for HIV testing? (check all that apply)
   - It was a regularly scheduled testing visit and it was time for me to get tested again
   - It was a visit before starting PrEP
   - It was a post-exposure prophylaxis (PEP) visit to be prescribed medicine after a potential exposure to HIV
   - I had symptoms that could be recent (primary) HIV infection
   - I had symptoms of an STI
   - I think I had an exposure to HIV
   - I had a new partner
   - I stopped having sex with someone
   - My doctor recommended that I get tested
   - Other (Why did you come in for HIV testing?___________ [REQUIRED])
2. When did you get your POC NAT result? [REQUIRED]
   - During my appointment
   - After my appointment
   - I didn’t get my results
3. **[skip question if #2 is “I didn’t get my results” or “During my appointment”]** How did you get your POC NAT result? [REQUIRED]
   - In clinic
   - By phone
   - Via my electronic medical record
   - I got them another way (How did you get your POC NAT result?___________ [REQUIRED])
4. **[skip question if #2 is “I didn’t get my results”]** What was the result of your POC NAT? [REQUIRED]
   - HIV negative
   - HIV positive
   - Invalid
   - I don’t remember
5. **[skip question if #2 is “I didn’t get my results”]** How acceptable was the way you received your POC NAT result? [REQUIRED]
   - Very unacceptable
   - Unacceptable
   - Slightly unacceptable
   - Slightly acceptable
   - Acceptable
   - Very acceptable
6. **[skip question if #2 is “I didn’t get my results” or if “#4 is “I don’t remember” or if #4 is “Invalid”]** I trust the accuracy of the POC NAT result. [REQUIRED]

- Strongly disagree
- Disagree
- Slightly disagree
- Slightly agree
- Agree
- Strongly agree

1. **[skip question if #2 is “I didn’t get my results” or if “#4 is “I don’t remember” or if #4 is “Invalid”]** My understanding of my POC NAT result is that it indicated: [REQUIRED]
   - I definitely do not have HIV
   - I am unlikely to have HIV
   - I might have HIV
   - I definitely have HIV
   - I don’t know
2. **[skip question if #2 is “I didn’t get my results” or if “#4 is “I don’t remember” or if #4 is “Invalid”]** Now that you have received your POC NAT result, what will you do with that information? (check all that apply) [REQUIRED]
   - I plan to start PrEP
   - I will retest later
   - I do not plan to retest
   - I will tell my partner/s my result
   - I will tell my primary care provider my result
   - I don’t plan to change anything
   - Other (Now that you have received your POC NAT result, what will you do with that information?___________ [REQUIRED])

8a. [**if “I will retest later” is marked for item 8**] When do you plan to retest?

- Within the next month
- About 3 months from now
- About 6 months from now
- About 9 months from now
- About 12 months from now
- Other (When do you plan to retest?___________)

**<PAGE BREAK>**

1. Please rate how strongly you disagree or agree with the following statements on the 6-point scale below. [REQUIRED]

|  | Strongly disagree | Disagree | Slightly disagree | Slightly agree | Agree | Strongly agree |
| --- | --- | --- | --- | --- | --- | --- |
| This is an acceptable test for HIV. |  |  |  |  |  |  |
| I think this test is effective in identifying HIV infection. |  |  |  |  |  |  |
| I would be willing to use this test again. |  |  |  |  |  |  |
| I did not like this test. |  |  |  |  |  |  |
| Overall, this test is more helpful than other tests for HIV. |  |  |  |  |  |  |
| I would recommend this test to others. |  |  |  |  |  |  |

1. My experience with the POC NAT was: [REQUIRED]
   - Very negative
   - Negative
   - Slightly negative
   - Slightly positive
   - Positive
   - Very positive

**<PAGE BREAK>**

[Conjoint Analysis Questions: Each participant will see nine combinations and will be asked which one is most preferred and which is least preferred. Cards and card sets will be created using partial factorial design to maximize contrasts in options participants are shown, to create about 36 sets. 100 HIV negative participants in each group (Seattle’s LGBTQ+ Center, Madison Clinic) will be given block sets of the identified options. The bold words below are ‘attributes’ and the list after them are ‘levels’ of each attribute, in the lingo of conjoint analysis. We plan to show each participant 9 questions with 3 cards in each question.]

- **Specimen type:** (oral fluid, fingerstick, blood draw)
- **Window period:** (2 weeks, 4 weeks, 12 weeks)
- **Time to results:** (20 min, 1 hour, 2 hours, 2 days)
- **What is the likelihood I might have a false positive?** (1 in 20 (5%), 1 in 100 (1%), 1 in 1000 (0.1%))

Now we are going to give you 12 sets of choices of HIV tests. Based on the characteristics listed, please tell us which HIV test is the one you would most prefer and which is the HIV test you would prefer least. Some of the options may reflect actual HIV tests that are available, but some are just imaginary for the purposes of this survey. The different factors included to consider are:

**Specimen type:** This is the type of sample used to run the test.

**Window period:** This is the average length of time from HIV exposure to when a test will give a positive result for HIV infection.

**Time to results:** This is how long it takes a test to give a result after you put in the sample.

**False positive:** This is when a test tells you that you are HIV positive when you really do NOT have HIV.

1. [Example 1] HIV Testing Preference – Question 1 of 12

| **Specimen type** | Oral fluid | Fingerstick | Blood draw |
| --- | --- | --- | --- |
| **Window period** | 2 weeks | 12 weeks | 4 weeks |
| **Time to results** | 2 hours | 2 hours | 2 days |
| **Chance that you’ll have a false positive** | 1 in 100 (1%) | 1 in 20 (5%) | 1 in 1000 (0.1%) |
| Most preferred | o | o | o |
| Least preferred | o | o | o |

[**if the same testing option is picked for #11]** You cannot choose the same option for most and least preferred HIV test. Please update your answer and select a different test.

1. [Example 2] HIV Testing Preference – Question 2 of 12

| **Specimen type** | Oral fluid | Blood draw | Blood draw |
| --- | --- | --- | --- |
| **Window period** | 12 weeks | 12 weeks | 4 weeks |
| **Time to results** | 20 minutes | 2 hours | 1 minute |
| **Chance that you’ll have a false positive** | 1 in 1000 (0.1%) | 1 in 20 (5%) | 1 in 100 (1%) |
| Most preferred | o | o | o |
| Least preferred | o | o | o |

[**if the same testing option is picked for #12]** You cannot choose the same option for most and least preferred HIV test. Please update your answer and select a different test.

1. [Example 3] HIV Testing Preference – Question 3 of 12

| **Specimen type** | Fingerstick | Fingerstick | Oral fluid |
| --- | --- | --- | --- |
| **Window period** | 2 weeks | 2 weeks | 4 weeks |
| **Time to results** | 2 hours | 20 minutes | 1 hour |
| **Chance that you’ll have a false positive** | 1 in 20 (5%) | 1 in 100 (1%) | 1 in 20 (5%) |
| Most preferred | o | o | o |
| Least preferred | o | o | o |

[**if the same testing option is picked for #13]** You cannot choose the same option for most and least preferred HIV test. Please update your answer and select a different test.

[There will be -12 such questions in the final survey.]

**<PAGE BREAK>**

Now we are going to ask you some questions about early HIV infection (also called primary HIV infection). As you probably know, HIV is a virus that weakens the immune system, leading to other infections, cancers, and AIDS. In the period of time right after someone gets infected with HIV, there is a special reaction by the immune system to the HIV that differs from when someone has been infected for a long time. We want to know what people know about early HIV infection so we can provide the best possible counseling and care.

1. Someone who was recently infected with HIV may have a negative HIV test. This is called the ‘window period’
   - True
   - False
2. Someone recently infected with HIV can spread HIV even if they have a negative test
   - True
   - False
3. Only one of the following statements about early HIV infection (primary infection) is true. Please choose the circle next to the statement that you think is true
   - Primary HIV infection can be asymptomatic (there can be no symptoms in primary infection).
   - Primary HIV infection is always symptomatic (There are always symptoms in primary infection).
4. Examples of “symptoms” include things like “cough” or “runny nose.” If you know any of the symptoms that can occur during primary HIV infection (symptoms people have when they first become HIV-infected), **please list as many specific symptoms of primary HIV infection as you can.)**

- _________________________

**<PAGE BREAK>**

PrEP, or pre-exposure prophylaxis, is HIV medicine for HIV-negative people to prevent getting HIV. Brand names of PrEP medicines are Truvada or Descovy. People take PrEP every day or right before getting exposed to HIV. The following questions are about your PrEP use. If you are not able to recall an exact number or date, it is okay to give an estimate.

1. Have you ever taken PrEP?
   - Yes, I’m currently on PrEP
   - Yes, but I’m not currently taking PrEP
   - No, I have never taken PrEP
2. [**if “Yes, I’m currently on PrEP” or “Yes, but I’m not currently taking PrEP” is marked for #15**] How long ago did you start PrEP?

- In the past month
- 1-6 months ago
- 6-12 months ago
- More than 1 year ago

1. **[if #18 is marked “Yes, I’m currently on PrEP”]** How many pills have you missed in the last 4 days? Please enter a number: _______
2. **[if #18 is marked “Yes, I’m currently on PrEP”]** How many pills have you missed in the last 30 days? Please enter a number: _________
3. **[if #18 is marked “Yes, I’m currently on PrEP”]** When was the last time you missed a pill of your PrEP medication?

- This week
- In the past month
- 1-3 months ago
- More than 3 months ago
- Never

1. **[skip if #22 is marked “Never]** What was the reason you missed your pills the last time you missed taking them? (check all that apply)
   - Forgot
   - Lost my medication
   - Was having side effects
   - Was feeling depressed
   - Didn’t want to
   - Didn’t want someone to see/know I was on PrEP
   - Ran out of medication
   - No reason
   - Other (What was the reason you missed your pills last time you missed taking them? _______________)
2. **[if #15 is marked “Yes, I’m currently on PrEP”]** Please rate your overall PrEP adherence

- (0-100% slider)

1. **[if “Yes, but I’m not currently taking PrEP” is marked for #17]** When did you stop taking PrEP?

- In the past month
- 1-6 months ago
- 6-12 months ago
- More than 1 year ago

1. **[if “Yes, but I’m not currently taking PrEP” is marked for #17]** What is the main reason you stopped taking PrEP (check one)?
   - I didn’t know enough about PrEP
   - I did not like taking a pill every day
   - I had side effects
   - I did not think I was risky enough to be on PrEP
   - I was not having enough sex/don’t have sex
   - I could not afford PrEP
   - Other (What is the main reason you stopped taking PrEP?___________________)
2. [**if “Yes, I’m currently on PrEP” is marked for #17**] What is the hardest thing about taking PrEP (check one)?
   - It is hard to remember to take every day
   - I am not sure it will prevent me from getting HIV
   - I don’t think my risk for HIV is high enough to take PrEP every day
   - It is not affordable
   - I worry about being judged for using PrEP
   - I had undesirable side effects
   - Seeing a provider every 3 months is a lot of trouble
   - PrEP may not be safe to use with other drugs I am taking
   - I do not have any concerns or challenges with taking PrEP
   - Other (What is the hardest thing about taking PrEP?________________)
3. [**if “Yes, I’m currently on PrEP” is marked for #17**] What is the main reason you are taking PrEP?
   - I have a positive partner
   - It decreases my anxiety about getting HIV
   - My partners prefer that I use PrEP
   - My doctor told me to
   - I feel good that I am taking care of my sexual health
   - Other (What is the main reason you are taking PrEP?____________)
4. [**if “Yes, but I’m not currently taking PrEP” is marked for #17]** What factors contributed to you stopping PrEP? (check all that apply)
   - It is hard to remember to take every day
   - I am not sure it will prevent me from getting HIV
   - I don’t think my risk for HIV is high enough to be on PrEP
   - It is not affordable
   - I worry about being judged for using PrEP
   - I had undesirable side effects
   - Seeing a provider every 3 months is a lot of trouble
   - PrEP may not be safe to use with other drugs I am taking
   - I did not have any concerns or challenges with taking PrEP
   - Other (What factors contributed to you stopping PrEP?________________)

**<PAGE BREAK>**

We will now ask you some questions about sexual experiences you've had in the past 12 months. We only want to know about partners you had oral, anal or vaginal sex with in the past 12 months.

Some questions will ask specifically about your sex partners' HIV status and what you did with partners who were HIV positive, HIV negative, and partners whose HIV status you did not know. We know you can't always be sure of a partner's HIV status. Please try to answer the questions based on what you knew and what your partners told you or did not tell you about their HIV status.

We know that it may be difficult to answer some of these questions if one or more of your sex partners do not identify as male or female, or if they are transgender. Except where we ask specifically about transgender partners, please include these sex partners in your responses to the questions about sex with either men or women, whichever you think is most appropriate.

We also know that how people refer to their genitals can be unique and personal. The following questions may use words that don’t perfectly fit how you or your partners refer to your bodies. Please answer the questions about specific sex acts the best that you can. If you are not able to recall an exact number or date when asked, it is okay to give an estimate.

In the past 12 months, did you have oral, anal, or vaginal sex partners of the gender identities listed below? (Cisgender is when your gender identity aligns with your sex at birth. Transgender is when your gender identity differs from your sex at birth.)

|  | Yes | No |
| --- | --- | --- |
| Cisgender men |  |  |
| Cisgender women |  |  |
| Transgender men |  |  |
| Transgender women |  |  |
| Non-binary or genderqueer people |  |  |
| People of another identity not listed |  |  |
| People whose gender identity you did not know |  |  |

1. In the past 12 months, with how many cisgender men have you had oral, anal, or vaginal sex? (Please enter a number, e.g. 2, do not type ‘one’) ______________________
2. In the past 12 months, with how many cisgender women have you had oral, anal, or vaginal sex? (Please enter a number, e.g. 2, do not type ‘one’) ____________________________________
3. In the past 12 months, with how many transgender men have you had oral, anal, or vaginal sex? (Please enter a number, e.g. 2, do not type ‘one’) ____________________________________
4. In the past 12 months, with how many transgender women have you had oral, anal, or vaginal sex? (Please enter a number, e.g. 2, do not type ‘one’) ____________________________________
5. In the past 12 months, with how many non-binary or genderqueer people have you had oral, anal, or vaginal sex? (Please enter a number, e.g. 2, do not type ‘one’) ____________________________________
6. In the past 12 months, with how many people of another identity not listed have you had oral, anal, or vaginal sex? (Please enter a number, e.g. 2, do not type ‘one’) ____________________________________
7. In the past 12 months, with how many people whose gender identity you did not know have you had oral, anal, or vaginal sex with? (Please enter a number, e.g. 2, do not type ‘one’)
8. [**if n>0 for “people of another identity not listed” for #30**] Please describe the gender identities of your partner(s) who were reported above: _______________________________________
9. [**if n>0 for “Total” for #30**] In the past 12 months, did you have condomless anal or vaginal sex with any partners who were HIV positive or whose HIV status you didn’t know?

- Yes
- No
- I don’t know

1. **[if n>0 for “Total” for #30**] In the past 12 months, how many group sex events (sex with 2 or more partners at the same time) have you participated in? Enter 0 if none.
   - Enter a number: _______
   - I don’t know
2. In the last 12 months has a health care provider told you that you had a sexually transmitted infection (STI) other than HIV?
   - Yes
   - No
3. [**if “Yes” is marked for #30**] Which STI(s)? (check all that apply)

- Chlamydia
- Gonorrhea
- Syphilis
- Other (Please list the other STIs___________________)

Thank you so much for completing this survey! We will send you a gift card via the same email address where we sent this survey. You can expect to receive the gift card in your email within two business days.

If you have any questions or concerns, you can contact Joanne Stekler (206-744-8312) or email our study team at GainStudy@uw.edu.

**<END SURVEY>**
