# Supplementary material for: Feasibility, Acceptability, and Test Performance of Point-of-Care Nucleic Acid Tests for HIV Testing and Viral Load Monitoring in the United States: Prospective Longitudinal Mixed-Methods Study
Source: JMIR Res Protoc. 2026 Jul 23;15:e84625. doi: 10.2196/84625 (PMC13395423; doi:10.2196/84625)
Supplement: Multimedia Appendix 4 [file resprot-v15-e84625-s004.docx]

**GAIN Study CASI Patient Acceptability Survey: Participants with HIV at Seattle’s LGBTQ+ Center** (formerly known as Gay City)

We are asking you to complete this survey because you recently participated in the GAIN study. We want to understand your experience with getting the point-of-care nucleic acid test (POC NAT). We will ask you questions about yourself, your participation in the study, and your experience with the POC NAT used at your recent visit.

Considering that some of the questions may be about sensitive topics, we suggest taking this survey in a private location.

This survey is completely voluntary, and you may stop at any time. We expect this survey to take about 20 minutes. After you finish taking the survey we will send you a $10 gift card for your time.

Please do not use your browser’s back button. If you do, you might have to restart the survey from the beginning. Instead, please use the “Previous Page” button if you need to go back to an earlier question.

If you have any questions or concerns, please contact Joanne Stekler (206-744-8312) or email our study team at GainStudy@uw.edu.

Thank you for your participation! Please click the ‘NEXT’ button to begin the survey.

**<PAGE BREAK>**

Please let us know your thoughts on the POC NAT that you had at your study visit:

1. Which of these describe why you sought a sexually transmitted infection (STI) test at Seattle’s LGBTQ+ Center (formerly known as Gay City)? (check all that apply)
   - It was a regularly scheduled testing visit and it was time for me to get tested again
   - I had symptoms that concerned me
   - I think I had an exposure
   - I had a new partner
   - I stopped having sex with someone
   - My doctor recommended that I get tested
   - My partner asked me to get tested
   - Another reason (Why did you seek an STI test at Seattle’s LGBTQ+ Center (formerly known as Gay City)? _________________)
2. If you could regularly get a POC NAT at Seattle’s LGBTQ+ Center (formerly known as Gay City), in addition to chlamydia, gonorrhea, and syphilis testing, what is the likelihood you would want one? [*REQUIRED*]

- Very unlikely
- Unlikely
- Somewhat unlikely
- Somewhat likely
- Likely
- Very likely

1. What would be the reason you would want a POC NAT at Seattle’s LGBTQ+ Center (formerly known as Gay City)? (check all that apply) [*REQUIRED*]
   - Reassurance that your anti-HIV medications (antiretroviral therapy, or ART) are working
   - Knowledge that you could not transmit to others
   - Costs associated with seeing my primary care provider are too high
   - I don’t feel comfortable seeing my primary care provider for HIV care
   - I feel judged when I go to my primary care provider for my HIV status
   - I feel judged when I go to my primary care provider for my gender identity
   - I feel judged when I go to my primary care provider for my sexual orientation
   - I feel judged when I go to my primary care provider for another reason
   - There are long wait times at my primary care provider
   - It is too hard to get an appointment at my primary care provider during hours that I am free
   - My primary care provider is too far away and Seattle’s LGBTQ+ Center (formerly known as Gay City) is more convenient
   - Another reason (Why would you want a POC NAT at Seattle’s LGBTQ+ Center (formerly known as Gay City)? _________________[*REQUIRED IF “ANOTHER REASON” IS MARKED*])
2. **[if #3 has “I feel judged when I go to my primary care provider for another reason” checked]** What are some of the other reasons why you feel judged when you go to your primary care provider?

- _______________________________________________[*REQUIRED IF “I FEEL JUDGED WHEN I GO TO MY PRIMARY CARE PROVIDER FOR ANOTHER REASON” IS MARKED*]

1. Would being able to get the POC NAT at Seattle’s LGBTQ+ Center (formerly known as Gay City) change how frequently you would go see your primary care provider? [*REQUIRED*]
   - Yes
   - No
   - Maybe
   - I don’t have a primary care provider
2. **[skip if #5 is “I don’t have a primary care provider”]** Please choose the best option from below to complete the sentence: This last visit was a visit…………. visiting my primary care provider. [*REQUIRED*]
   - in addition to
   - in place of

**<PAGE BREAK>**

1. When did you get your POC NAT result? [*REQUIRED*]

- During my appointment
- After my appointment
- I didn’t get my results

1. **[skip question if #7 is “I didn’t get my results” or “During my appointment”]** How did you get your POC NAT result? [*REQUIRED*]
   - In clinic
   - By phone
   - Via my electronic medical record
   - I got them another way (How did you get your POC NAT result?___________ [*REQUIRED IF “I GOT THEM ANOTHER WAY” IS MARKED*])
2. **[skip question if #7 is “I didn’t get my results”]** What was the result of your POC NAT? [*REQUIRED*]
   - >1000 copies RNA
   - <1000 copies RNA
   - Invalid
   - I don’t remember
3. **[skip question if #7 is “I didn’t get my results”]** How acceptable was the way you got your POC NAT result? [*REQUIRED*]
   - Very unacceptable
   - Unacceptable
   - Slightly unacceptable
   - Slightly acceptable
   - Acceptable
   - Very acceptable
4. **[skip question if #7 is “I didn’t get my results” or if #9 is “Invalid” or if #9 is “I don’t remember”]** I trust the accuracy of the POC NAT result. [*REQUIRED*]

- Strongly disagree
- Disagree
- Slightly disagree
- Slightly agree
- Agree
- Strongly agree

1. **[skip question if #7 is “I didn’t get my results” or if #9 is “Invalid” or if #9 is “I don’t remember”]** My understanding of my POC NAT result from my research visit is that it showed (choose the best option): [*REQUIRED*]
   - I am HIV positive
   - I have a high level of HIV in my blood
   - My viral load is below the cutoff for the test
   - I am undetectable
   - I don’t know
   - Other (My understanding of my POC NAT result from my research visit is that it showed:___________ [*REQUIRED IF “OTHER” IS MARKED*])
2. **[skip question if #7 is “I didn’t get my results” or if #9 is “Invalid” or if #9 is “I don’t remember”]** Now that you have received your POC NAT result, what will you do with that information? (check all that apply) [*REQUIRED*]
   - Nothing will change
   - I will start taking anti-HIV medications (antiretroviral therapy, or ART)
   - I will work on taking my pills every day
   - I will see my primary care provider again sooner
   - I will tell my partner/s my result
   - I will tell my primary care provider my result
   - My primary care provider and I plan to change my anti-HIV medications
   - My primary care provider and I already changed my anti-HIV medications
   - Other (Now that you have received your POC NAT result, what will you do with that information? ______[*REQUIRED IF “OTHER” IS MARKED*])

**<PAGE BREAK>**

1. Please rate how strongly you disagree or agree with the following statements on the 6-point scale below. [*REQUIRED*]

|  | Strongly disagree | Disagree | Slightly disagree | Slightly agree | Agree | Strongly agree |
| --- | --- | --- | --- | --- | --- | --- |
| This is an acceptable test for HIV. |  |  |  |  |  |  |
| I think this test is effective in identifying HIV infection. |  |  |  |  |  |  |
| I would be willing to use this test again. |  |  |  |  |  |  |
| I did not like this test. |  |  |  |  |  |  |
| Overall, this test is more helpful than other tests for HIV. |  |  |  |  |  |  |
| I would recommend this test to others. |  |  |  |  |  |  |

1. My experience with the POC NAT was: [*REQUIRED*]
   - Very negative
   - Negative
   - Slightly negative
   - Slightly positive
   - Positive
   - Very positive

**<PAGE BREAK>**

1. **[skip question if #7 is “I didn’t get my results” or if #9 is “Invalid” or if #9 is “I don’t remember”]** How likely are you to share the result of your POC NAT with the people listed in the table below? [*REQUIRED*]

|  | Very unlikely | Unlikely | Somewhat unlikely | Somewhat likely | Likely | Very likely | Not applicable |
| --- | --- | --- | --- | --- | --- | --- | --- |
| a. Your sex partner/s |  |  |  |  |  |  |  |
| b. Your needle-sharing partner/s |  |  |  |  |  |  |  |
| c. Your friends |  |  |  |  |  |  |  |
| d. Your family |  |  |  |  |  |  |  |
| e. Your dating or hookup app profile/s |  |  |  |  |  |  |  |

1. Are you currently on anti-HIV medications (antiretroviral therapy, or ART)? [*REQUIRED*]
   - Yes
   - No
   - I don’t know
2. **[if #17 is “Yes”]** The viral load cutoff level of this test is 1000 copies of viral HIV. This means that this test will be able to tell you if the amount of virus per milliliter of your blood is greater or less than 1000 copies of HIV. The test that you get in your clinic can tell you if your viral load is above or below a much lower level (often about 40-50 copies per milliliter of blood).

If your test said your viral load was below the cutoff level of 1000 copies per milliliter, how confident would you be that your anti-HIV medications are working? [*REQUIRED*]

- - Not confident at all
  - Not very confident
  - Somewhat confident
  - Very confident

1. **[if #17 is “Yes”]** At what viral load cutoff level would you feel confident that your anti-HIV medications are working? [*REQUIRED*]
   - - No level – I will always worry about transmitting HIV
     - Less than 40 copies
     - Less than 200 copies
     - Less than 1000 copies
     - Less than 1500 copies
     - Less than 5000 copies
     - Other (At what viral load cutoff level would you feel confident that your anti-HIV medication____________[*REQUIRED IF “OTHER” IS MARKED*])
     - I don’t know
2. The viral load cutoff level of 1000 copies makes me feel confident that I will not transmit HIV. [*REQUIRED*]
   - Strongly disagree
   - Disagree
   - Slightly disagree
   - Slightly agree
   - Agree
   - Strongly agree
3. At what viral load cutoff level would you feel confident that you will not transmit HIV? [*REQUIRED*]
   - - No level – I will always worry about transmitting HIV
     - Less than 40 copies
     - Less than 200 copies
     - Less than 1000 copies
     - Less than 1500 copies
     - Less than 5000 copies
     - Other (At what viral load cutoff level would you feel confident that you will not transmit HIV?____________ [*REQUIRED IF “OTHER” IS MARKED*])
     - I don’t know
4. Have you heard of HIV undetectable = untransmittable (U=U)?
   - Yes
   - No
   - Not sure

**<PAGE BREAK>**

The next following set of questions will ask you about how you take your anti-HIV medications, called antiretroviral therapy or ART. We want to know a little bit about the anti-HIV medications you may be taking. If you are not able to recall an exact number or date when asked, it is okay to give an estimate.

1. **[if #17 is marked “Yes”]** How many pills have you missed in the last 4 days? Please enter a number: _______
2. **[if #17 is marked “Yes”]** How many pills have you missed in the last 30 days? Please enter a number: _________
3. **[if #17 is marked “Yes”]** When was the last time you missed a pill of your anti-HIV medications (antiretroviral therapy or ART)?

- This week
- In the past month
- 1-3 months ago
- More than 3 months ago
- Never

1. **[skip if #25 is marked “Never]** What was the reason you missed your pills the last time you missed taking them ? (check all that apply)
   - Forgot
   - Lost my medication
   - Was having side effects
   - Was feeling depressed
   - Didn’t want to
   - Didn’t want someone to see/know I was positive
   - Ran out of medication
   - No reason
   - Other (What was the reason you missed your pills the last time you missed taking them? _______________)
2. **[if #17 is marked “Yes”]** Please rate your overall anti-HIV medications (antiretroviral therapy or ART) adherence. 0% would mean that you never take your medicine, 50% means you take them about half the time, and 100% means you never miss a pill.

- (0-100% slider)

1. **[skip if #5 is “I don’t have a primary care provider”]** Did your primary care provider discuss adherence with you at your last visit?
   - Yes
   - No
   - I don’t remember

**<PAGE BREAK>**

Thank you so much for completing this survey! We will send you an gift card via the same email address where we sent this survey. You can expect to receive the gift card in your email within two business days.

If you have any questions or concerns, you can contact Joanne Stekler (206-744-8312) or email our study team at GainStudy@uw.edu.

**<END SURVEY>**
