# Supplementary material for: Feasibility, Acceptability, and Test Performance of Point-of-Care Nucleic Acid Tests for HIV Testing and Viral Load Monitoring in the United States: Prospective Longitudinal Mixed-Methods Study
Source: JMIR Res Protoc. 2026 Jul 23;15:e84625. doi: 10.2196/84625 (PMC13395423; doi:10.2196/84625)
Supplement: Multimedia Appendix 5 [file resprot-v15-e84625-s005.docx]

**GAIN Study CASI Patient Acceptability Survey: Participants with HIV at Madison Clinic**

We are asking you to complete this survey because you recently participated in the GAIN study. We want to understand your experience with getting the point-of-care nucleic acid test (POC NAT). We will ask you questions about yourself, your participation in the study, and your experience with the POC NAT used at your recent visit.

Considering that some of the questions may be about sensitive topics, we suggest taking this survey in a private location.

This survey is completely voluntary, and you may stop at any time. We expect this survey to take about 20 minutes. After you do the survey we will send you a $10 gift card for your time.

Please do not use your browser’s back button. If you do, you might have to restart the survey from the beginning. Instead, please use the “Previous Page” button if you need to go back to an earlier question.

If you have any questions or concerns, please contact Joanne Stekler (206-744-8312) or email our study team at GainStudy@uw.edu.

Thank you for your participation! Please click the ‘NEXT’ button to begin the survey.

**<PAGE BREAK>**

Please let us know your thoughts on the POC NAT that you had at your study visit:

1. When did you get your POC NAT result? [*REQUIRED*]

- During my appointment
- After my appointment
- I didn’t get my results

1. **[skip question if #1 is “I didn’t get my results” or “During my appointment”]** How did you get your POC NAT result? [*REQUIRED*]
   - In clinic by my provider
   - In clinic by study staff
   - By phone by my provider
   - By phone by study staff
   - Via my electronic medical record
   - I got them another way (How did you get your POC NAT result?___________ [*REQUIRED IF “I GOT THEM ANOTHER WAY” IS MARKED*])
2. **[skip question if #1 is “I didn’t get my results”]** What was the result of your POC NAT? [*REQUIRED*]
   - >1000 copies RNA
   - <1000 copies RNA
   - Invalid
   - I don’t remember
3. **[skip question if #1 is “I didn’t get my results”]** How acceptable was the way you received your POC NAT result? [*REQUIRED*]
   - Very unacceptable
   - Unacceptable
   - Slightly unacceptable
   - Slightly acceptable
   - Acceptable
   - Very acceptable
4. **[skip question if #1 is “I didn’t get my results” or if #3 is “Invalid” or if #3 is “I don’t remember]** I trust the accuracy of the POC NAT results. [*REQUIRED*]

- Strongly disagree
- Disagree
- Slightly disagree
- Slightly agree
- Agree
- Strongly agree

1. **[skip question if #1 is “I didn’t get my results” or if #3 is “Invalid” or if #3 is “I don’t remember]** My understanding of my POC NAT result from my research visit is that it showed (choose one): [*REQUIRED*]
   - I am HIV positive
   - I have a high level of HIV in my blood
   - My viral load is below the cutoff for the test
   - I am undetectable
   - I don’t know
   - Other: (My understanding of my POC NAT result from my research visit is that it showed:__________ [*REQUIRED IF “OTHER” IS MARKED*])
2. **[skip question if #1 is “I didn’t get my results” or if #3 is “Invalid” or if #3 is “I don’t remember]** Now that you have received your POC NAT result, what will you do with that information? (check all that apply) [*REQUIRED*]
   - Nothing will change
   - I will start taking anti-HIV medications (antiretroviral therapy, or ART)
   - I will work on taking my pills every day
   - I will see my primary care provider again sooner
   - I will tell my partner/s my result
   - I will talk to my primary care provider about my result
   - My primary care provider and I plan to change my anti-HIV medications
   - My primary care provider and I already changed my anti-HIV medications
   - Other (Now that you have received your POC NAT result, what will you do with that information?___________ [*REQUIRED IF “OTHER” IS MARKED*])

**<PAGE BREAK>**

1. Please rate how strongly you disagree or agree with the following statements on the 6-point scale below. [*REQUIRED*]

|  | Strongly disagree | Disagree | Slightly disagree | Slightly agree | Agree | Strongly agree |
| --- | --- | --- | --- | --- | --- | --- |
| This is an acceptable test for HIV. |  |  |  |  |  |  |
| I think this test is effective in identifying HIV infection. |  |  |  |  |  |  |
| I would be willing to use this test again. |  |  |  |  |  |  |
| I did not like this test. |  |  |  |  |  |  |
| Overall, this test is more helpful than other tests for HIV. |  |  |  |  |  |  |
| I would recommend this test to others. |  |  |  |  |  |  |

1. My experience with the POC NAT was: [*REQUIRED*]
   - Very negative
   - Negative
   - Slightly negative
   - Slightly positive
   - Positive
   - Very positive

**<PAGE BREAK>**

1. **[skip question if #1 is “I didn’t get my results” or if #3 is “Invalid” or if #3 is “I don’t remember]** How likely are you to share the results of your POC NAT with the people listed below? [*REQUIRED*]

|  | Very unlikely | Unlikely | Somewhat unlikely | Somewhat likely | Likely | Very likely | Not applicable |
| --- | --- | --- | --- | --- | --- | --- | --- |
| Your sex partner/s |  |  |  |  |  |  |  |
| Your needle-sharing partner/s |  |  |  |  |  |  |  |
| Your friends |  |  |  |  |  |  |  |
| Your family |  |  |  |  |  |  |  |
| Your dating or hookup app profile/s |  |  |  |  |  |  |  |

1. When do you usually get your viral load results? [*REQUIRED*]
   - At my next visit
   - I call the clinic
   - I look in my chart online
   - I get them another way (When do you usually get your viral load results?__________ [*REQUIRED IF “I GOT THEM ANOTHER WAY” IS MARKED*])
2. Are you currently on anti-HIV medications (antiretroviral therapy or ART)? [*REQUIRED*]
   - Yes
   - No
   - I don’t know
3. **[if #12 is “Yes”]** The viral load cutoff level of this test is 1000 copies of viral HIV. This means that this test will be able to tell you if the amount of virus per milliliter of your blood is greater or less than 1000 copies of HIV. The test that you get in your clinic can tell you if your viral load is above or below a much lower level (often about 40-50 copies per milliliter of blood).

If your test said your viral load was below the cutoff level of 1000 copies per milliliter, how confident would you be that your anti-HIV medications are working? [*REQUIRED*]

- - Not confident at all
  - Not very confident
  - Somewhat confident
  - Very confident

1. At what viral load cutoff level would you feel confident that your anti-HIV medications are working? [*REQUIRED*]
   - No level – I will always worry about transmitting HIV
   - Less than 40 copies
   - Less than 200 copies
   - Less than 1000 copies
   - Less than 1500 copies
   - Less than 5000 copies
   - Other (At what viral load cutoff level would you feel confident that your anti-HIV medications are working?____________ [*REQUIRED IF “OTHER” IS MARKED*])
   - I don’t know
2. The viral load cutoff level of 1000 copies makes me feel confident that I will not transmit HIV. [*REQUIRED*]
   - Strongly disagree
   - Disagree
   - Slightly disagree
   - Slightly agree
   - Agree
   - Strongly agree
3. At what viral load cutoff level would you feel confident that you will not transmit HIV? [*REQUIRED*]
   - No level – I will always worry about transmitting HIV
   - Less than 40 copies
   - Less than 200 copies
   - Less than 1000 copies
   - Less than 1500 copies
   - Less than 5000 copies
   - Other (at what viral load cutoff level would you feel confident that you will not transmit HIV? __________[*REQUIRED IF “OTHER” IS MARKED*])
   - I don’t know
4. Have you heard of HIV undetectable = untransmittable (U=U)?
   - Yes
   - No
   - Not sure

**<PAGE BREAK>**

The next following set of questions will ask you about how you take your anti-HIV medications, called antiretroviral therapy, or ART. We want to know a little bit about the anti-HIV medications you may be taking. If you are not able to recall an exact number or date when asked, it is okay to give an estimate.

1. [**if #12 is marked “Yes”]** How many pills have you missed in the last 4 days? Please enter a number: __________
2. [**if #12 is marked “Yes”]** How many pills have you missed in the last 30 days? Please enter a number: __________
3. **[if #12 is marked “Yes”]** When was the last time you missed a pill of your anti-HIV medications (antiretroviral therapy, or ART)?

- This week
- In the past month
- 1-3 months ago
- More than 3 months ago
- Never

1. **[skip** **if #18 is marked “Never”]** What was the reason you missed your pills the last time you missed them? (check all that apply)
   - Forgot
   - Lost my medication
   - Was having side effects
   - Was feeling depressed
   - Didn’t want to
   - Didn’t want someone to see/know I was positive
   - Ran out of medication
   - No reason
   - Other (What was the reason you missed your pills the last time you were taking them? _______________)
2. **[if #12 is marked “Yes”]** Please rate your overall anti-HIV medication (antiretroviral therapy or ART) adherence. 0% would mean that you never take your medicine, 50% means you take them about half the time, and 100% means you never miss a pill.

- (0-100% slider)

**<PAGE BREAK>**

1. Your provider had a conversation with you about your anti-HIV medications (antiretroviral therapy, or ART) adherence at your visit. We would like to know how you feel about that conversation. Please rate how strongly you disagree or agree with each statement on the 6-point scale below. [*REQUIRED; skipped if participant didn’t talk about POC NAT result with provider*]

|  | Strongly disagree | Disagree | Slightly disagree | Slightly agree | Agree | Strongly Agree |
| --- | --- | --- | --- | --- | --- | --- |
| The conversation was helpful to me. |  |  |  |  |  |  |
| My provider believed what I had to say. |  |  |  |  |  |  |
| I did not receive strategies to help me take my medications. |  |  |  |  |  |  |
| The conversation helped me to make changes to help me take my medications. |  |  |  |  |  |  |
| The conversation did not help me to understand how my medications work. |  |  |  |  |  |  |
| During the conversation, we discussed other options for my medications. |  |  |  |  |  |  |

**<PAGE BREAK>**

1. We would like to know how you feel about your visit with the provider who gave you your POC NAT result. Please rate how strongly you disagree or agree with the following statements on the 5-point scale below. *(skipped if participant didn’t talk about POC NAT result with provider)*

|  | Totally disagree | Disagree | Neutral | Agree | Totally agree |
| --- | --- | --- | --- | --- | --- |
| I doubt that my provider really cares about me as a person. |  |  |  |  |  |
| My provider is usually considerate of my needs and puts them first. |  |  |  |  |  |
| I trust my provider so much I always try to follow his/her advice. |  |  |  |  |  |
| If my provider tells me something is so, then it must be true. |  |  |  |  |  |
| I sometimes distrust my provider’s opinions and would like a second one. |  |  |  |  |  |
| I trust my provider’s judgements about my medical care. |  |  |  |  |  |
| I feel my provider does not do everything he/she should about my medical care. |  |  |  |  |  |
| I trust my provider to put my medical needs above all other considerations when treating my medical problems. |  |  |  |  |  |
| My provider is well qualified to manage (and diagnose and treat or make an appropriate referral) medical problems like mine. |  |  |  |  |  |
| I trust my provider to tell me if a mistake was made about my treatment. |  |  |  |  |  |
| I sometimes worry that my provider may not keep the information we discuss totally private. |  |  |  |  |  |

**<PAGE BREAK>**

Thank you so much for completing this survey! We will send you an gift card via the same email address where we sent this survey. You can expect to receive the gift card in your email within two business days.

If you have any questions or concerns, you can contact Joanne Stekler (206-744-8312) or email our study team at GainStudy@uw.edu.

**<END SURVEY>**
