# Supplementary material for: Feasibility, Acceptability, and Test Performance of Point-of-Care Nucleic Acid Tests for HIV Testing and Viral Load Monitoring in the United States: Prospective Longitudinal Mixed-Methods Study
Source: JMIR Res Protoc. 2026 Jul 23;15:e84625. doi: 10.2196/84625 (PMC13395423; doi:10.2196/84625)
Supplement: Multimedia Appendix 6 [file resprot-v15-e84625-s006.docx]

**Participant Interview Guide**

**Gay City and Madison Clinic HIV Testing Participants**

**Introduction:**

Thank you for participating in this discussion. You have been invited to participate in this conversation because you recently participated in a GAIN study visit. The purpose of the GAIN Study is to better understand patient perspectives on how we can use point-of-care nucleic acid tests or NAT in different types of settings. In this conversation, we hope to learn more from you about your thoughts on the point-of-care NAT.

This [focus group or interview] will last about 45 minutes to 1 hour. We will audio-record the conversation. We are doing this so that we can listen to the recording and create a written transcript of the conversation. This way we can capture all of the details of the conversation. We will give you a $40 gift card for your participation. If you have any questions about the study, please contact Joanne Stekler (206-744-8312).

Do you have any questions or concerns? *[The interviewer will answer any questions that arise.]* Okay, thank you. I am going to start recording our conversation now. *[The interviewer will turn on the audio recorder.]*

**Sample Questions:**

- Based on everything you currently know about HIV testing, what does your ideal HIV test look like?
  - Probe: which assets of an HIV test are most important to you, between the window period, test time, false positive rate, and specimen collection method? Could you rank the factors from most important to least important to you?
- To the best of your knowledge, what is the point-of-care NAT, and what does it test for?
  - Probe: The point-of-care NAT is the test you got at your study visit. What were you told about this test at your study visit?
  - Probe: How is the point-of-care NAT different from other HIV tests?
  - Probe [FG only]: Does anyone know anything else about point-of-care NATs?
  - Probe: What are some reasons or situations where point-of-care NAT may be useful as compared to other HIV testing options?
  - Probe: What do you know about the window period for point-of-care NAT?
  - Probe: A “window period” for an HIV test is the time between when someone gets infected and when a test could turn positive.
  - Probe: Can you tell us if there are any symptoms that would make you concerned that you might have gotten HIV, if you’d had a possible exposure?
  - Probe: some tests look for Antibodies and some test for the rNA of the virus… do you remember what the POC NAT tests for?
- Describe your overall experience of taking the point-of-care NAT test and receiving your results.
  - Probe: Describe how you were tested using point-of-care NAT. i.e. blood draw, wait times
  - Probe: How did you receive your point-of-care NAT result? I.e. in person, via phone, text, or email?
  - Probe: What did you like or not like about that method of testing and/or receiving the test result from the point-of-care NAT?
  - Probe: What would you change about the process of being tested and/or receiving the test result from the point-of-care NAT?
- As you may know, the point-of-care NAT usually takes around 2 hours to return a result. If the point-of-care NAT takes two hours from the time the test starts until you get a result, what would be the best way for you to get your result during a testing visit?
  - Probe: Would you be willing to come in before your appointment?
  - Probe: Would you be willing to return after your appointment?
- Based on your current knowledge, how trustworthy are the results you received from the point-of-care NAT?
- What did you do with the information gained after receiving your point-of-care NAT results?
  - Probe: Who did you, or will you, share this information with, if anyone?
  - Probe: How did you change your behavior, or, what actions did you take, if any, after receiving your results?
  - Probe: What impact did receipt of your point-of-care NAT results have on any decisions regarding pre-exposure prophylaxis (PrEP)?
- Would you recommend the point-of-care NAT to others? Why or why not?

[*Prepare and distribute individual show cards for HIV diagnostic testing options that describe type of test (nucleic acid tests (NAT), antigen/antibody tests, and antibody), type of specimen collection, wait times, quant/qual results, etc.]*

- Here are placards that describe several options for HIV testing that may or may not be familiar to you. (For FG: As a group, please discuss these options aloud based on their appeal and your preferences. Then, as a group, rank order them from most preferred (first) to least preferred (last).)

[*Examples of cards to be shown:*]

|  | Point-of-care NAT (SAMBA) | POC Ab test (INSTI) | POC OF Ab test | Lab Based Ag/Ab test | POC Ab Test (Determine) |
| --- | --- | --- | --- | --- | --- |
| **Specimen type:** | fingerstick | Fingerstick | Oral fluid | Blood draw | fingerstick |
| **Window period:** | 2 weeks | 4 weeks | 12 weeks | 3 weeks | 4 weeks |
| **Time to results:** | 2 hours | 20 minutes | 20 minutes | 2 days | 20 minutes |
| **False positive:** | 1 in 1000 (0.1%) | 1 in 100 (1%) | 1 in 100 (1%) | 1 in 1000 (0.1%) | 1 in 20 (5%) |

- Remember to describe your rationale and logic when making any relevant statements. [*Probes to understand different components of preference if not voiced aloud:*]
- What specimen collection method do you prefer?
- Which test type do you trust the most to give you the correct test result?
- What is the most important factor in your ranking?
- Can you rank the factors from most important to least important to you?
- Which factor is most important to decision making for you?
- How would the importance of the ‘time to results’ factor change if your result were to be positive?
- Ask folks for a final ranking at the end

- Is there anything else you would like to add regarding the study or the point-of-care NAT test?

Thank you very much for sharing your thoughts and opinions! We will provide you with a $40 gift card as a stipend for your participation. If you have any questions about the study, please contact Joanne Stekler (206-744-8312).
