# Supplementary material for: Feasibility, Acceptability, and Test Performance of Point-of-Care Nucleic Acid Tests for HIV Testing and Viral Load Monitoring in the United States: Prospective Longitudinal Mixed-Methods Study
Source: JMIR Res Protoc. 2026 Jul 23;15:e84625. doi: 10.2196/84625 (PMC13395423; doi:10.2196/84625)
Supplement: Multimedia Appendix 7 [file resprot-v15-e84625-s007.docx]

**Participant Interview Guide**

**PWH at the Center**

**Introduction:**

Thank you for participating in this discussion. You have been invited to participate in this conversation because you recently participated in a GAIN study visit. The purpose of the GAIN Study is to better understand patient perspectives on how we can use point-of-care nucleic acid tests or NAT in different types of settings. In this conversation, we hope to learn more from you about your thoughts on the point-of-care NAT.

This interview will last about 45 minutes to 1 hour. We will audio-record the conversation. We are doing this so that we can listen to the recording and create a written transcript of the conversation. This way we can capture all of the details of the conversation. We will provide you with a $40 gift card for your participation. If you have any questions about the study, please contact Joanne Stekler (206-744-8312).

Do you have any questions or concerns? *[The interviewer will answer any questions that arise.]* Okay, thank you. I am going to start recording our conversation now. *[The interviewer will turn on the audio recorder.]*

**Sample Questions:**

- The point-of-care NAT is the test you got at your study visit. What were you told about this test at your study visit?
  - Probe: How is point-of-care NAT different from standard viral load testing?
  - Probe: What are some reasons or situations where point-of-care NAT may be useful as compared to standard viral load testing?
- Describe your overall experience of taking the point-of-care NAT test and receiving your results.
  - Probe: What would you change about the way you were tested and/or how you received your results?
  - Probe: Describe how you were tested using point-of-care NAT. i.e. blood draw, wait times
  - Probe: How did you receive your point-of-care NAT result? I.e. in person, via phone, text, or email?
  - Probe: What did you like or not like about that method of testing and/or receiving the test result from the point-of-care NAT?
  - Probe: What would you change about the process of being tested and/or receiving the test result from the point-of-care NAT?
- As you may know, the point-of-care NAT test usually takes around 2 hours to return a result. Since that is the case, what would be the best way to get your result during your visit?
  - Probe: Would you be willing to come in before your appointment?
  - Probe: Would you be willing to return after your appointment?
- How did getting your point-of-care NAT results compare with how you usually get your viral load results?
- Probe: do you usually get your viral load results in person, over the phone, or some other way?
- Based on your current knowledge, how trustworthy are the results you received from the point-of-care NAT?
- Would you recommend point-of-care NAT to others? Why or why not?
- What did you do with the information gained after receiving your point-of-care NAT results?
  - Probe: Who did you, or will you, share this information with, if anyone?
  - Probe: How did you change your behavior, or, what actions did you take, if any, after receiving your results?
  - More specific probe: Did getting your NAT results change whether or how you took your HIV medicines
  - More specific probe: Did getting your NAT results change anything about your sexual health, for example your number of partners or whether you would use condoms with your sex partners?
- The typical detection limit of laboratory viral load tests is between 40-200 copies in a milliliter of blood (if asked, can say that there are 5 mL in a teaspoon). The detection limit of this point-of-care NAT is about 1000 copies of the virus. How does the limit of detection of the point-of-care NAT affect your opinion of how good it is for monitoring your HIV and medicines?
  - Probe: How does knowing about that limit of detection change what you think about what the test can tell you about your own health and how well your medicines are working?
  - Probe: What limit would you want to see? Why?
- People living with HIV who have an undetectable viral load by taking their anti-HIV medicines have no risk of transmitting the virus to others. The chance of transmission of the virus goes up as the viral load goes up How confident do you feel that you would not transmit the virus if the point-of-care NAT said you had a viral load of <1000 copies?
  - Probe: How confident do you feel if the laboratory viral load test said <200 copies?
- Can you tell me the reasons why you or some other person might seek out STI testing at Gay City rather than at your primary care provider?
- Would you use point-of-care NAT for viral load testing or monitoring at a community-based organization like Gay City? Why or why not?
  - Probe: How would it change how often you see your primary care provider?

- When you get your viral load checked in your doctor's office, those results get reported to Public Health, no matter if your virus is detectable or undetectable. Public Health uses this information to check to see whether you are getting HIV care and to monitor how we are doing in providing care to people living with HIV across the state. If you were to get a point-of-care viral load test at Gay City but not at your doctor's office, it might not get reported to Public Health. What are your thoughts on this?
  - Probe: If your viral load doesn't get reported, public health staff might think that you are not getting HIV care and might call you or send you mail to see how you are doing. How does that change your thinking about reporting of the point-of-care viral load results?

[*Prepare and distribute individual show cards for viral load testing options that describe type of test (PCR, several point-of-care NAT options), wait times, limits of detection, quant/qual results, etc.*]

- Here are placards that describe several options for HIV viral load monitoring that may or may not be familiar to you. As a group, please discuss these options aloud based on their appeal and your preferences. Then, as a group, rank order them from most preferred (first) to least preferred (last).

[*Cards to be shown:*]

|  | PCR | POC NAT 1 (SAMBA) | POC NAT 2 (Cepheid) |
| --- | --- | --- | --- |
| **Specimen type:** | Blood draw | Fingerstick | Blood Draw |
| **Result type:** | Quantitative | Semi Quantitative | Quantitative |
| **Time to results:** | 2 days | 2 hours | 2 hours |
| **Limit of Detection:** | 40 copies | 1000 copies | 40 copies |
| **Performed by:** | Laboratory | Clinic staff | Clinic staff |

- Remember to describe your rationale and logic when making any relevant statements.

Probes to understand different components of preference if not voiced aloud:

- What specimen collection method do you prefer?
- Which test type do you trust the most to give you the correct test result?
- What is the most important factor in your ranking?
- Is there anything else you would like to add regarding the study or the point-of-care NAT test?

Thank you very much for sharing your thoughts and opinions! We will follow up with a $40 gift card as a stipend for your participation. If you have any questions about the study, please contact Joanne Stekler (206-744-8312).
